# miR-10b-5p regulates adipocyte lineage commitment and adipogenesis via targeting of Gata6 and Tubby

**DOI:** 10.1186/s12964-026-02834-y

**Published:** 2026-03-28

**Authors:** Nikoletta Kalenderoglou, Federica Dimitri, Carmen Navarro González, Antonio Vidal-Puig, Jacob Hobbs, Awais Younis, Constantinos Christodoulides, Stefania Carobbio, Mark Christian

**Affiliations:** 1 Department of Biosciences, School of Science and Technology, Nottingham Trent University, Nottingham, NG11 8NS UK; 2 Centro de Investigación Principe Felipe, CIBERDEM, Valencia, Spain; 3 Metabolic Research Laboratories, Wellcome Trust-MRC Institute of Metabolic Science, University of Cambridge, Cambridge, UK; 4 Oxford Centre for Diabetes, Endocrinology and Metabolism, Radcliffe Department of Medicine, University of Oxford, Oxford, UK; 5 NIHR Oxford Biomedical Research Centre, John Radcliffe Hospital, Oxford, UK

**Keywords:** miR-10b-5p, Tub, Gata6, Adipocyte browning, Embryonic stem cell, Adipogenesis, Obesity

## Abstract

**Background:**

Adipogenesis is a highly organised series of events that facilitates the healthy expansion of adipose tissue, beginning during embryogenesis and continuing throughout life. White adipogenesis protects against lipotoxicity, influencing insulin resistance and obesity-related comorbidities. Brown adipogenesis enhances energy expenditure, thereby counteracting weight gain, lipotoxicity and insulin resistance. Recently, there has been a significant increase in interest regarding adipocyte differentiation, mainly focusing on the interplay between microRNAs (miRNAs) and the transcriptional cascade that governs adipogenesis and metabolic dysfunction. This study aimed to identify miRNAs regulating white and brown adipocyte differentiation and define miRNA action in a stem cell model of adipogenesis.

**Methods:**

Small RNAseq analysis of primary mouse brown and white adipocytes (WAs) identified miR-10b-5p to be upregulated in mature brown adipocytes (BAs). We generated two model systems: (1) immortalized brown preadipocytes treated with miRNA inhibitors and (2) CRISPR/Cas9 KO of miR-10b in E14 mouse embryonic stem cells (mESCs). Both cell models were differentiated into mature adipocytes. To unravel the pathways that are affected by miR-10b-5p depletion, a transcriptomic analysis was performed at key time points.

**Results:**

Both cell models showed that miR-10b-5p depletion severely impaired differentiation into mature adipocytes, as indicated by a lack of lipid droplet formation and reduced adipogenic gene expression. Gene expression analysis supports that miR-10b-5p directs embryonic stem (ES) cells towards the mesoderm lineage, promoting commitment to preadipocytes by downregulating Gata6 and its downstream target Bmp2. Our study further demonstrated that miR-10b-5p regulates the later stages of adipogenesis, at least in part, by downregulating Tub, a direct target of miR-10b-5p. We also confirmed that miR-10b-5p alleviated the halted differentiation phenotypes of adipocytes by suppressing the G Protein signaling pathway mediated by Tubby.

**Conclusions:**

These results evidence that miR-10b-5p inhibition plays a dynamic role in adipocyte biology, as its inhibitory effects manifest differently during the stem cell preadipocyte proliferation state and during the maturation phase of adipocytes. Collectively, our study demonstrated that miR-10b-5p may represent a new potential therapeutic target for lipodystrophy and obesity.

**Supplementary Information:**

The online version contains supplementary material available at 10.1186/s12964-026-02834-y.

## Background

Adipogenesis is a coordinated process regulated by multiple autocrine, paracrine and endocrine signals. It facilitates the healthy expansion of adipose tissue, beginning during embryogenesis and continuing throughout life [1]. It is generally regarded as a two-step process: a commitment step, wherein a fibroblast-like mesenchymal stem cell (MSC) commits to the adipocyte lineage without any morphological alterations, developing into a preadipocyte. This commitment is then followed by differentiation, wherein committed preadipocytes expand, undergo growth arrest following by contact inhibition, and take on the characteristics of functional mature adipocytes as they acquire the machinery that is essential for lipid transport and synthesis, insulin sensitivity and the secretion of adipocyte-specific proteins [2].

In mammals, two major types of adipose tissue exist, white (WAT) and brown (BAT). WAT plays a critical role in the regulation of energy homeostasis by storing excess energy in the form of triglycerides (TGs), secreting adipokines that regulate lipid metabolism, secreting inflammatory cytokines, and controlling insulin sensitivity [3, 4]. The lipids in adipose tissue are produced by *de novo* lipogenesis, and the hydrolysis and intracellular re-esterification of TGs from circulating lipoproteins. They serve not only as a caloric reservoir for future need but also as a protective agency in cells to sequester lipids away from peripheral tissues that are susceptible to lipotoxicity, which disrupts insulin action on metabolism [5]. In contrast to WAT, BAT is composed of multiple smaller (multilocular) lipid droplets. Classical BAT is characterised as a temperature mediator organ specialised for oxidizing fatty acids and glucose to produce heat through non-shivering thermogenesis [6, 7].

Adipocytes, like other mesenchymal cells, emerge from the mesodermal layer of the embryo. Specifically, white adipocytes (WAs) are believed to derive from the lateral plate mesoderm, whereas brown adipocytes (BAs) are developed from paraxial mesoderm [8]. Furthermore, MSCs can be committed to either an adipogenic lineage of myogenic factor 5 (Myf5)-negative cells or a myogenic lineage of Myf5-positive cells [9]. Previous studies using lineage tracing experiments in mouse models have demonstrated that WAs emerge from the adipogenic lineage, whereas cells expressing Myf5 give rise to skeletal myocytes and BAs in interscapular and perirenal depots [10]. Improved knowledge of adipogenesis is necessary to gain insight into brown and white fat physiology. Interest in adipocyte differentiation has increased markedly over the past few years, with emphasis on the intersection between miRNAs and the transcriptional cascade that controls adipogenesis and metabolic dysfunction.

miRNAs are endogenous, noncoding small RNAs that play a key role in regulating various developmental and cellular functions. They control gene expression at the posttranscriptional level by binding their seed sequence to complementary regions in the 3’ untranslated region (UTR) of target mRNAs, leading to either the destabilization of the mRNA or the suppression of its translation [11, 12]. miRNA targets are highly tissue- or cell type-dependent, and a single miRNA can perform diverse functions by controlling different mRNAs in different tissues [11, 13].

In search of miRNAs crucial for adipogenesis, we identified miR-10b-5p as a novel regulator through small RNA sequencing of mouse BAs and WAs. miR-10b-5p was upregulated in BAs, and its depletion in immortalized brown pre-adipocyte and stem cell models impaired differentiation into mature adipocytes. Our findings strongly suggest that miR-10b-5p depletion impairs the early stages of adipogenesis, specifically the commitment of ESCs to preadipocytes. This effect may be driven by directing cells toward the mesoderm lineage through the upregulation of Gata6 and its downstream target, Bmp2. RNA sequencing further revealed that ablation of miR-10b-5p led to a significant increase in genes related to G Protein signaling associated with elevated Tubby (Tub) which was identified as direct target of miR-10b-5p. The suppression in adipocyte differentiation after miR-10b-5p inhibition was partially reversed by Tub silencing. Our research highlights the pleiotropic regulatory role of miR-10b-5p during adipogenesis.

## Methods

### Cell culture

Primary cultures of subcutaneous white adipose tissue (scWAT) and interscapular brown adipose tissue (iBAT) were collected from 7-day-old C57BL/6 mice. scWAT was digested in DMEM-OK, which consists of DMEM/F12 (Sigma) supplemented with 2% BSA, 16 µM biotin (B4501, Sigma), 1.8 µM pantothenic acid (24330100, Acros Organics), and 100 µM ascorbic acid (A8960, Sigma), along with 2 mg/ml collagenase A (Sigma). The iBAT was digested using a digestion buffer composed of 100 mM HEPES (pH 7.4), 123 mM NaCl, 5 mM KCl, 3 mM CaCl₂, 5 mM glucose, 1.5% BSA, and 1 mg/ml collagenase A in DMEM-OK. The mixture was incubated with agitation at 37°C for 30 minutes. The digested tissue was then filtered and centrifuged to isolate the stromal vascular fraction (SVF). The SVF pellet from scWAT was resuspended and treated with a lysis buffer containing 100 ml of Solution A (2.08 g TRIS, pH 7.65, in 100 ml distilled H2O) and 900 ml of Solution B (8.3 g NH4Cl in 1000 ml distilled H2O), before centrifugation again. The scWAT cell pellet was resuspended in DMEM-OK supplemented with 10% NCS. Cells from scWAT were then plated in a 6-well plate, maintained till confluency and differentiated in DMEM-OK with 10% NCS, supplemented with 5 μg/ml insulin, 33.33 nM dexamethasone (D2915, Sigma), 1 μg/ml transferrin (03-0124SA, Gibco), and 2 ng/ml T3 (T6397, Sigma). Media was changed every 2–3 days, with differentiation occurring within a week. The SVF pellet from iBAT was washed once more in DMEM-OK, centrifuged for 5 min at 2300 rpm and plated in 6 well plates. Details of the induction and maintenance media for brown adipocytes are presented in Supplementary Table 1. Small RNA-Seq was performed with RNA from primary white and brown preadipocytes before and after differentiation.

Primary adipocytes and SVF were isolated from three different adipose tissues: gonadal WAT, subcutaneous WAT and Classical BAT. After collection and digestion, cells were filtered through a 250 μm mesh and left to stand on ice for 30 minutes. Primary adipocytes were collected (floating fraction), and the rest was further centrifuged (10 min, 700g). Remaining mature adipocytes were collected (floating fraction), and the SVF (pelleted fraction) was further processed by washing with DMEM media and centrifuging (10 min, 700g). Last, the supernatant was removed, and the pellet was processed for RNA analysis. 

Immortalised brown and white adipocyte cell lines were generated by culturing the cells from the SVF of iBAT and scWAT of 10-week-old female 129 Sv mice with retroviral-mediated expression of temperature-sensitive simian virus 40 (SV40) large T-antigen (H-2Kb-tsA58) [14].

Human adipose tissue samples were obtained from patients undergoing elective surgical neck dissections after obtaining informed consent. Human brown preadipocytes were isolated using the methodology described by Collins et al. [15] and subsequently immortalized using the pLenti6.3/V5-DEST lentiviral expression system to co-express human telomerase reverse transcriptase (hTERT) and human papillomavirus type-16 E7 oncoprotein (HPV16-E7), as previously described by Pinnick et al. [16]. The study was approved by the South Central Research Ethics Committee (REC reference: 12/SC/0446; IRAS project number: 101619).

HEK293, primary cultures and immortalised cell lines were cultured in DMEM/F12 medium (Sigma) supplemented with 10% FBS Serum 10500 (Gibco), 1% L-Glutamine and 1% Penicillin-Streptomycin antibiotics (Gibco). 

Mouse embryonic stem cells (mESCs) were cultured in Glasgow's MEM (GMEM) (Invitrogen) supplemented with 10% FBS, 2 mM L-Glutamine (Sigma), 0.5% Penicillin-Streptomycin (Gibco), 0.1 mM MEM Non-Essential Amino Acids Solution (Gibco), 0.25% Sodium Bicarbonate solution (Gibco, 7.5% stock), 1 mM Sodium Pyruvate (Gibco), 0.1 mM 2-Mercaptoethanol (Gibco) and 1000 units/ml Leukemia Inhibitory Factor (LIF) (Merck Millipore) on 0.1% gelatin (Millipore)-coated flasks. 

Immortalised BAT and WAT were cultured at 33°C and 5% CO2, whereas HEK293, primary cultures and mESCs were cultured at 37°C in a humidified incubator at 5% CO2. Cells were passaged when they reached 50 – 70% confluence.

### Adipocyte differentiation

Mouse brown and white adipocytes were initially seeded in plates, pre-coated with 0.1% gelatin, at 90% confluency one day prior to differentiation. When cells reached confluency, plates were transferred to a 37°C incubator and differentiation was initiated. Two distinct protocols were employed to induce the differentiation of brown and white adipocytes. The components and their concentrations in the induction and maintenance media for white and brown adipocytes are detailed in Supplementary Tables 1 and 2, respectively. Human brown preadipocytes were seeded onto gelatin-coated 12-well plates and cultured at 37°C in 5% CO₂ in DMEM containing 10% FBS. Upon reaching ~80% confluency, differentiation was initiated. Induction and maintenance media were prepared in Advanced DMEM/F12 supplemented with 3% FBS, 1% penicillin/streptomycin, and 1% L-glutamine (A-DMEM3), as detailed in Supplementary Table 3. Induction medium was refreshed every 3 days. From day 12 onward, the concentration of all compounds was reduced by two-fold every 2 days. Prior to experimentation, the brown adipocytes were maintained in maintenance medium for 48 hours.

Human pluripotent stem cells were induced to differentiate into mature BAs, as previously described [17]. mESCs were induced to differentiate into mature adipocytes following the established protocol described earlier [18]. This adipogenic protocol includes components (T3 and Rosiglitazone) known to promote and maintain the thermogenic program.

### RNA extraction and RT-qPCR

For total RNA isolation, cells were collected in Trizol reagent (Invitrogen) and isolated as described in the manufacturer’s protocol. In brief, cells were lysed in 1 ml TRIzol reagent. Chloroform (TRI reagent®: Chloroform 5:1 v/v) was added. Cell mixtures were vigorously shaken for 15 seconds, incubated at RT for 3 minutes and centrifuged at 12,000 x g for 15 minutes at 4 °C. Next, the upper aqueous phase was transferred to a new tube and twice the volume of isopropanol (Sigma-Aldrich) was added for precipitation. After washing with 75% ethanol, the pellets were air dried and dissolved in nuclease-free water. RNA quantity and quality were analysed by a NanoDrop spectrophotometer (Thermo Scientific).

The extracted RNA was reverse transcribed into cDNA. Initially, the RNA samples were incubated with Amplification Grade DNase I (Sigma-Aldrich) for 15 minutes. To neutralize DNase activity, EDTA stop solution was added to each sample and incubated at 65°C for 10 minutes. For reverse transcription, M-MLV Reverse Transcriptase (Sigma-Aldrich) was utilised to generate cDNA strands. Each sample received the following components: 1 µl Random Hexamer Primer (Thermo Fisher), 1 µl dNTP Mix (Promega), and 4.5 µl water. After incubating for 10 minutes at 70 °C, the remaining components were added: 2 µl of 10x M-MLV Reverse Transcriptase Buffer, 1 µl M-MLV Reverse Transcriptase, 4.5 µl water, and 0.5 µl RNase OUT™ (Invitrogen). The reaction underwent incubation at 25°C for 10 minutes to facilitate the annealing of random primers. Subsequently, RNA was reverse transcribed at 37°C for 50 minutes, followed by denaturation at 80 °C for 10 minutes.

For relative quantification of miRNAs, the miRCURY LNA RT Kit (Qiagen) was used for reverse transcription into cDNA. The concentration of DNase-treated cellular RNA was adjusted to 5ng/µl in RNase-free water and reverse transcribed according to the manufacturer’s protocol. For quantitative real-time PCR (RT-qPCR), the Applied Biosystems QuantStudio 7 Pro real-time PCR system (Thermo Scientific) was used. RT-qPCR for genomic or protein-coding genes was performed by utilizing SYBR® Green JumpStart™ Taq ReadyMix™ (Sigma-Aldrich) according to the manufacturer's guidelines. The list of primer sequences is provided in Supplementary Table 4 and 5. 

The RT-qPCR analysis of miRNA was carried out by using the miRCURY SYBR Green PCR Kit in combination with miRCURY LNA miRNA PCR primers (339306, QIAGEN) for mmu-miR-10b-5p (YP00205637), U6 snRNA (YP02119464), mmu-miR-709 (YP00205463), mmu-miR-706 (YP00205976), hsa-miR-138-5p (YP00206078), hsa-miR-196a-5p (YP00204386), hsa-miR-365a-3p (YP00204622), mmu-miR-455-3p (YP00205432), mmu-miR-222-3p (YP02119325) and mmu-miR-378a-3p (YP00204179) following the instructions of the manufacturer. The relative fold change was calculated using the formula 2-ΔΔCt.

### Oil red O staining

On the final day of adipocyte differentiation, cell monolayers were fixed with 4% paraformaldehyde (PFA) in PBS for 15 minutes at room temperature. A 0.25% (w/v) Oil Red O stock solution was prepared in 60% isopropanol, then diluted 6:4 with water to create the working solution. The cells were stained with this solution for 1 hour. Excess stain was removed by briefly washing with 60% isopropanol, followed by PBS.

### Luciferase assay and mutagenesis assay

miR-10b targets were predicted with TargetScan (targetscan.org) and MirWalk (mirwalk.umm.uni-heidelberg.de). Mouse Gata6 (1045 bp) and Tubby (2128bp) 3′-UTRs were amplified by PCR (primers Supplementary Table 6) and cloned into the pRL-TK vector (Promega) using the XbaI and EagI restriction sites downstream of the Renilla reporter gene. Mutagenesis was performed using the QuickChange Site-directed Mutagenesis Kit (Agilent) according to the manufacturer's protocol. The sequence accuracy of all clones was verified by sequencing.

For luciferase activity assays, HEK293 cells in 48-well plates were co-transfected with locked nucleic acid (LNA) miR-10b mimic (5 nM) or control oligos (Exiqon) with the luciferase reporters using Lipofectamine™ RNAiMAX (Invitrogen). Luciferase activity was measured 48 h after transfection. The pGL3-Control vector (Promega) containing the firefly reporter gene was co-transfected into all cells to normalize the results. Firefly and Renilla luciferase activities were analysed using the Dual-Luciferase reporter assay system (Promega) according to the manufacturer’s instructions. Luciferase activity was quantified using a microplate reader, Infinite 200 Pro (Tecan). Renilla luciferase activity was normalized to Firefly luciferase activity. 

### sgRNA plasmid construction and introduction into mESCs

CRISPR sgRNAs were designed using the Optimized CRISPR Design tool CHOPCHOP [19–21]. Considering the significance of Drosha and Dicer processing sites during the process of miRNA biogenesis, the sgRNA sequence for miR-10b was designed within/adjacent to these sites. The sgRNA sequence for miR-10b was 5’- CCTGTAGAACCGAATTTGTGtgg-3’ (protospacer adjacent motif (PAM) sequences depicted in lower case letters). The sgRNA design was also evaluated for off-target effects using the web tool, CCTop - CRISPR/Cas9 target online predictor [22].

The chosen single-stranded oligonucleotides containing the guide sequence of the gRNA were annealed and cloned into the BbsI linearised px330-U6-Chimeric_BB_CBh-huSpCas9 vector (Addgene plasmid # 42230); [23]. The non-targeting sgRNA (Addgene plasmid # 42230) was used as a negative control. The generated CRISPR-Cas9 vectors harbouring either the gRNA targeting miR-10b or a non-targeted control (NTC) were transfected into the cells (Supplementary Table 7). To generate clonal miR-10b KO cell lines, fluorescence-activated cell sorting (FACS) was utilized 48 h post-transfection. Individual cells exhibiting GFP fluorescence were isolated through sorting using BD FACSMelody™ Cell Sorter (BD Biosciences, UK) and expanded. Cells were centrifuged to remove culture media, washed in DPBS with Ca^+ 2^ and Mg^+ 2^, and then suspended in DPBS prior to analysing and sorting them by FACS. Cells were initially gated for the intact cell population using forward scatter versus side scatter plots and then gated for transfected cells based on the presence of the transfected control. Transfected cells were gated for GFP-positive cells on the side scatter versus GFP plots. Following cell clone isolation and expansion, the clones were screened and identified by PCR, followed by High-Resolution Melt Analysis (HRMA) [24]. Sanger sequencing analysis of the miR-10b locus was used to confirm the indels created by CRISPR/Cas9. In addition, we generated three clonal non-targeted cell lines as a negative control by transfecting ESCs with the CRISPR/Cas9 plasmid harbouring NTC sgRNA.

### Extraction of genomic DNA and High-Resolution Melt Analysis (HRMA) 

DNA extraction: Half of the cells from the clonal cell colony were transferred to a new well for expansion, while the remaining cells were briefly centrifuged. To the pellet, 50 µl of QuickExtract™ DNA Extraction Solution (Cambio) was added and mixed thoroughly. The mixture was then subjected to a heat treatment at 65°C for 6 minutes, followed by 98°C for 2 minutes. Subsequently, the sample was centrifuged again to pellet the cell debris.

To screen CRISPR/Cas9-induced mutations by HRMA [25], a 124 bp fragment that included the entire genomic target site was PCR amplified. Primers flanking the target site were used to amplify the amplicon (Supplementary Table 7). PCR reactions contained 5 µl of SYBR^®^ Green JumpStart™ Taq ReadyMix™ master mix (Sigma), 1 µl of each primer (10 µM), 2 µl of DNA and water up to 10 µl (nuclease-free water; Qiagen). HRMA data was collected on the Applied Biosystems QuantStudio 7 Pro real-time PCR system (Thermo Scientific) and analysed using Excel (Microsoft Office).

### Transfection (miRNA inhibitors, miRNA mimics and siRNA)

All miRNA mimics and miRNA inhibitors were purchased from QIAGEN. All oligonucleotide sequences and catalogue numbers are shown in Supplementary Table 8. RNAiMAX (Invitrogen) was used to deliver miRNA inhibitors and mimics into cells as per the manufacturer’s instructions. Silencer^®^ Select siRNA against Tub (assay ID: s75577, cat. 4390771), Silencer^®^ Select siRNA against Gata6 (assay ID: s66490, cat. 4390771) and Silencer™ Select Negative Control No. 1 siRNA (cat. 4390843) were purchased from Thermo Fisher Scientific. Briefly, ESCs, BAs or WAs were plated in twelve-well dishes at 2-2.5 × 10^5^ cells/well in media without antibiotics and transfected with either 50 nM miRNA inhibitors or 5 nM miRNA mimics for adipocytes and 250 nM miRNA mimics for mESCs. For Tub and Gata6 knockdown (KD) assays, cells were treated with 5 nM of siRNA. Forty-eight hours later, cells were treated with induction medium. Cells were also treated with a negative control that did not exhibit any homology to the mouse sequence in the NCBI and miRBase databases.

### Measurement of oxygen consumption rates

Oxygen consumption rates (OCR) were measured using the Seahorse XFe24 Analyzer (Seahorse Bioscience). IBAT and scWAT cells were seeded into 0.1% gelatin-coated 24-well Seahorse Microplates (Seahorse Bioscience) at a density of 10,000 cells/well and treated with miR-10b inhibitor or mimic. The following day, cells were induced to differentiate using the standard protocols outlined in Supplementary Tables 1 and 2. Each experimental group included at least five replicates. The assay was conducted in sterile, unbuffered Assay Media prepared with Seahorse base media (Seahorse Bioscience) at 37 °C (pH 7.4), and supplemented with Sodium Pyruvate (1mM, Sigma), Glucose (10 mM, Sigma). Mature adipocytes were washed twice in this supplemented Seahorse media to fully remove the maintenance media and were then incubated for 1 h at 37 °C with no CO_2_. Mitochondrial function and potential were analysed by sequential injection of 1 µM oligomycin (ATP synthase inhibitor), 1 µM Carbonyl cyanide-4 (trifluoromethoxy) phenylhydrazone (FCCP, mitochondrial uncoupler) together with 2 mM pyruvate to achieve maximal respiration, and 2 µM rotenone and antimycin A (mitochondrial complex I and III inhibitors, respectively). Seahorse measurements were normalized to cell number using Hoechst 33,258 fluorescence quantified immediately after the assay [26]. Respiration, acidification, and ATP production rates were calculated following the Agilent Seahorse XF Technology instructions.

### Western blotting

Whole-cell lysates for Western blotting were extracted with RIPA lysis buffer containing Halt™ Protease Inhibitor Cocktail (ThermoFisher Scientific). Equal amounts of protein were resolved on 10% SDS-PAGE gels and transferred onto nitrocellulose membrane by using a Trans-Blot Turbo System (BioRad) set at 1.3 A/25 V/10 min. Membranes were blocked with 3% non-fat milk for 1 h at 20 °C and incubated overnight at 4 °C with the following primary antibodies (1:1000): GATA6 (84987-5-RR, Proteintech), UCP1 (ab10983, Abcam), TUB (17928-1-AP, Proteintech) and GAPDH (MAB374, Sigma Aldrich). Membranes were then incubated for 1 h at room temperature with the appropriate secondary antibody Goat Anti-Rabbit IgG (H + L) or Goat Anti-Mouse IgG (H + L), conjugated to IRDye® 800CW or IRDye® 680RD (926-32211, 926-68071, 926-68070, 926-32210, Licor Biosciences) at a 1:15,000 dilution in TBS/T containing 0.1% Tween. Washing steps were repeated after the incubation. Membranes were visualized with Odyssey CLx Imager (Licor Biosciences). Densitometry was performed via EmpiriaStudio (Licor Biosciences) software to measure the expression of the protein of interest.

### RNA-sequencing analysis

For small RNA sequencing, a total amount of 3 µg total RNA per sample was used as input material for library preparation. Sequencing libraries were generated using NEBNext^®^ Multiplex Small RNA Library Prep Set for Illumina^®^ (NEB, USA). The clustering of the index-coded samples was performed on a cBot Cluster Generation System using TruSeq SR Cluster Kit v3-cBot-HS (Illumina) according to the manufacturer’s instructions. After cluster generation, the library preparations were sequenced on an Illumina platform and 50 bp single-end reads were generated. Small RNA analysis was performed by Novogene Ltd (Cambridge, UK). Briefly, raw FASTQ reads were processed using custom Perl and Python scripts to remove reads containing poly-N, with 5’ adapter contaminants, without 3’ adapter or the insert tag, containing poly A/T/G/C and low-quality reads from raw data. All the downstream analyses were based on the clean data with high quality. The small RNA tags were mapped to reference sequence using Bowtie [27] allowing no mismatches, to analyse their expression and distribution. Mapped small RNA tags were used to identify known miRNAs. miRBase20.0 was used as reference. miRNA expression levels were quantified using TPM (transcripts per million) based on the criteria outlined by Zhou et al. [28]. The normalization formula used was: Normalized expression = (mapped read count / total reads) × 1,000,000. The miRNA expression dataset can be accessed through ArrayExpress under accession number E-MTAB-15,230.

For bulk RNA sequencing, 400 ng of total RNA per sample was used as input for library preparation. RNA library construction and transcriptome sequencing were performed by Novogene Ltd (Cambridge, UK). Regarding the transcriptomic analysis performed on mESCs differentiated to mature adipocytes, independent biological sample replicates from three different NTC clones and KO clones (Supplementary Table 10) were used for RNA sequencing. For the transcriptomic analysis performed on mouse BAT treated with miR-10b inhibitor or negative control, 4 independent biological sample replicates were used for RNA sequencing. RNA sequencing data of miR-10b +/+ and miR-10b –/– mESCs during adipocyte differentiation (Days 0, 12, and 27) are available under ArrayExpress accession E-MTAB-15,151. Data from the RNA sequencing analysis of miR-10b-5p knockdown (KD) during brown adipogenesis is available under E-MTAB-15,152.

Bioinformatic analysis of the RNA sequencing samples was performed in the R statistical computing environment version 4.3.2 and RStudio version 2024.12.0 using a published pipeline adopted from an open-source toolkit [29].

Kallisto [30] was used to generate read counts from a transcriptome reference. Kallisto transcript abundance measurements were imported into RStudio using the R package tximport (version 1.28.0) [31]. The Bioconductor/R package edgeR *cpm* function was used to create a list of counts per million (cpm) per transcript [32]. Genes included in the analysis had > 1 cpm in at least 3 samples. The edgeR (version 4.0.4) function *calcNormFactors* was used to normalize the filtered data using the TMM method (Trimmed Mean of M-values) [33]. The voom function from the Bioconductor/R package *limma* (version 3.58.1) [34] was used to establish a mean-variance relationship and model its trend. The lmFit function was then applied to fit a linear model to the data, while makeContrasts was utilized to define contrasts in the design matrix. Bayesian statistics were applied to the linear model fit using the eBayes function. To identify differentially expressed genes (DEGs), the decideTests function was used, applying multiple testing correction via the Benjamini-Hochberg method and strict cutoff criteria (corrected p-value ≤ 0.05 and log₂ fold change ≥ 1) to generate a list of significantly regulated DEGs [35]. Principal Component Analysis (PCA) plots were generated by using prcomp(). The Bioconductor/R package *ggplot2* (version 3.4.4) was used to create volcano plots. Heatmaps were generated using the heatmap.2 function of the R package *gplots*. The Bioconductor/R packages *msigdbr* (version 7.5.1), *clusterProfiler* (version 4.10.0) and *enrichplot* (version 1.22.0) were utilized to conduct gene set enrichment analyses using the C2 curated database from the Molecular Signatures Database (MSigDB). GO pathway analysis was performed using the Bioconductor/R package gprofiler2 (version 0.2.2) [34], and additional pathway analysis was conducted using QIAGEN Ingenuity Pathway Analysis (IPA) software.

### Statistical analysis

Experimental data are presented as mean ± standard error of the mean (SEM) and were analysed with GraphPad Prism (GraphPad Software, Inc.). The normal distribution of the data was assessed using the Shapiro-Wilk normality test. For normally distributed data, group comparisons were performed using two-tailed t-tests, one-way ANOVA, or two-way ANOVA, followed by Sidak’s multiple comparisons test where appropriate. For non-normally distributed data involving more than two groups, the Kruskal–Wallis test was used, followed by Dunn's multiple comparison test. For non-normally distributed data involving two groups, the Mann–Whitney U test was used. Details of the specific tests used in each experiment are provided in the corresponding figure legends. A significance level of p < 0.05 was deemed significant.

### Cellular senescence 

Cell senescence was visualized via a β-galactosidase staining protocol [36]. For positive control, cells were treated with 500 µM H_2_O_2_ prior to β-galactosidase staining. Stained cells, which appear as light blue cells, were observed via light microscopy at ×20 magnification using a DMi1 Inverted Microscope, (Leica Microsystems).

### miRNA PCR array

Differential secretion of miRNAs in conditioned media was assessed from undifferentiated and differentiated brown and white adipocytes conditionally immortalised cell lines using miRCURY LNATM Universal RT microRNA PCR Mouse & Rat panel I+II comprehensive for 752 miRNA assays. Cells were initially seeded in a 6 well plate and left in serum-free media for 24 hours to allow miRNA accumulation. Media samples were collected and processed through serial centrifugations, filtered, and then stored at -80 °C. Subsequent steps were carried out at Exiqon Service, where RNA was isolated and reverse transcribed. The resulting cDNA was analysed using the miRCURY LNA™ Universal RT microRNA PCR Mouse & Rat panel I+II. The scanning was performed using the Agilent G2565BA Microarray Scanner System (Agilent Technologies, Inc., USA) and the image analysis was carried out through the ImaGene 9 (miRCURY LNA™ microRNA Array Analysis Software, Exiqon, Denmark).

### Immunofluorescence

Cells were plated on 0.1% gelatin-coated coverslips, washed in PBS, fixed with 4% PFA for 10 min and permeabilized for 5 min with 0.1% Triton X-100 in PBS. Cells were washed with PBS and blocked with 5% goat serum in PBS for 1 h at RT, followed by overnight incubation with primary antibodies TUB (1:400; 17928-1-AP, Proteintech), PGC-1 (1:500; AB3242, Merck Millipore) and UCP1 (1:250; sc-6528, Santa Cruz Biotechnology) diluted in 1% BSA in PBS. Cells were washed with PBS and incubated for 1 h at RT with either goat anti-rabbit IgG Alexa Fluor 488 (1:1000; A-11008, Invitrogen) or donkey anti-goat IgG Alexa Fluor 555 (1:1000; A-21432, Invitrogen). Cells were rinsed with PBS and incubated in LipidTOX-Deep Red (1:2000, Thermo-Fisher) or AUTODOT™ Visualization Dye (1:2000, SM1000b, abcepta) for 1 h at RT. Following incubation, cells were stained with 300 nM DAPI for 40 min and coverslips were mounted on glass slides with ProLong antifade reagent. Images were obtained with either a Leica SP5 confocal microscope or Nikon AX R confocal microscope. Images were processed using FIJI/ImageJ software.

### Cell proliferation assay

Cells were seeded at 1 million per well in a 6-well plate, incubated for 48 hours, then counted and passaged. This process was repeated every 48 hours for 8 passages, with reseeding at 1 million cells per well each time. Cell counts were recorded at each passage to monitor proliferation trends.

## Results

### Identification of differentially expressed miRNAs between WAT and BAT

**Fig. 1:**
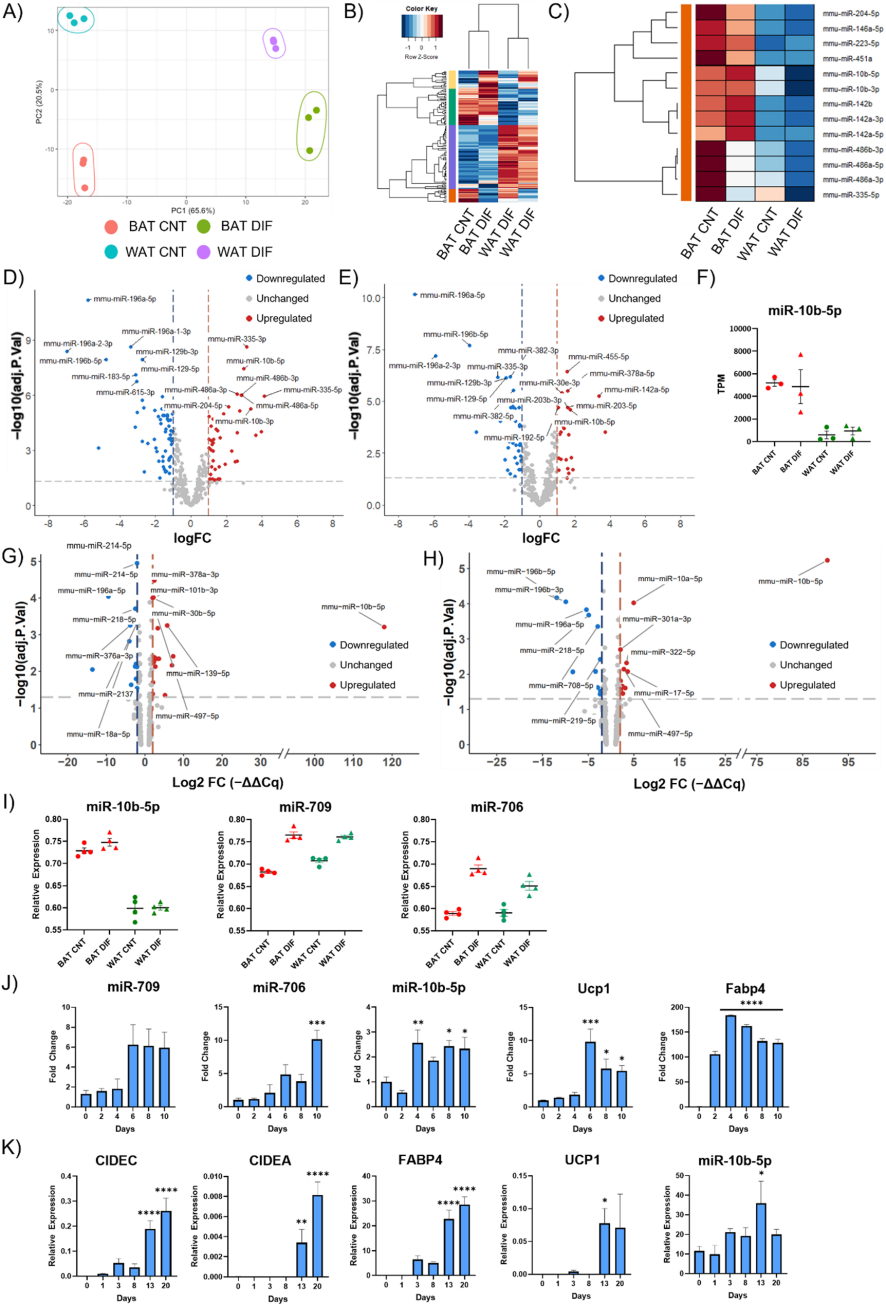
Comparative Analysis of miRNA Expression and Secretion During Brown and White Adipocyte Differentiation. **A** PCA of Small RNA sequencing data from brown and white adipocyte differentiation. **B** The most dynamically expressed miRNAs are shown in the heatmap. **C** The right heatmap includes a number of selected miRNAs that are highly expressed in BAT compared to WAT (orange module). **D** and (**E)** Volcano plots for the comparison between undifferentiated and differentiated BAT and WAT primary cultures. **F** Scatterplot represents the expression levels of miR-10b-5p as measure in TPMs. Solid horizontal black line represents the mean ± SEM. **G** and (**H**) Volcano plots were constructed by plotting the p-value on the y-axis and the log_2_ fold change (−ΔΔCq) between undifferentiated BAT vs. WAT and differentiated BAT vs. WAT on the x-axis. Highlighted spots are microRNAs exhibiting a log_2_ fold change of 2 or above and with p-values below 0.05 after Benjamini-Hochberg correction for multiple testing. The top 6 miRNAs are labelled for undifferentiated BAT vs. WAT and differentiated BAT vs. WAT. **I** Scatterplots represent the relative secretion levels of miR-10b-5p, miR-706 and miR-709. Data normalized with UniSp3. **J** Expression profiles of miR-10b-5p, miR-706, and miR-709 during mouse BAT differentiation, together with the expression levels of the adipogenic and thermogenic markers Fabp4 and Ucp1. **K** Time-course analysis of CIDEC, CIDEA, FABP4, UCP1, and miR-10b-5p expression during human brown adipocyte differentiation. Data were analysed using one-way ANOVA. Each day’s mean is compared to the mean of day 0, with significance assessed for each comparison. Data represent the mean ± SEM (*p < 0.05, **p < 0.005, ***p < 0.0005, ****p < 0.0001)

To identify miRNAs differentially expressed among undifferentiated and differentiated brown and white adipocytes, we profiled miRNA expression of preadipocytes isolated from the SVF of scWAT and iBAT, undifferentiated or differentiated into mature brown and white adipocytes by Small RNA Sequencing. Principal component analysis (PCA) of cellular miRNAs showed that samples clustered into 4 distinct groups, indicating that each group had a distinct miRNA profile (Fig. 1A). Small RNAseq analysis identified 126 differentially expressed miRNAs in mature adipocytes and preadipocytes based on log_2_ fold change of 2 and adjusted p-values < 0.05 with Benjamin-Hochberg correction (Fig. 1B). We focused on brown fat-enriched miRNAs to identify the miRNAs that might play a critical role in the brown phenotype of adipocytes. In BAT versus WAT preadipocytes, there were 61 upregulated and 45 downregulated miRNAs, whereas in differentiated BAT versus WAT, there were 28 upregulated and 42 downregulated miRNAs based on log_2_ fold change of 1 and presented a p value ≤ 0.05 (Fig. 1D and E). Among significantly regulated miRNAs, miR-10b-5p was identified as consistently enriched in BAs (Fig. 1C and F).

### Profile of differentially secreted miRNAs among undifferentiated and differentiated WAT and BAT

miRNAs have also been found extracellularly in biological fluids. Circulating miRNAs may facilitate paracrine and endocrine crosstalk between tissues and cells, thereby influencing gene expression and cellular activities. Adipose tissue is a major contributor to the pool of secreted miRNAs. Consequently, changes in adipose tissue mass or function, common in various metabolic conditions, can alter circulating miRNAs, which then exert systemic effects [37]. We analysed the differentially secreted miRNAs in conditioned media from undifferentiated and differentiated brown and white adipocytes. We identified 143 miRNAs common to all samples, with an average of 225 miRNAs detectable per sample. Variation among the assays was analysed by PCA, unsupervised two-way hierarchical clustering and pairwise volcano plots (Fig. 1G, H and S1).

The PCA plot shows clearly that the four sample groups have very distinct miRNA profiles. The most significant contributor to the observed variance was the differentiation status of the cells (Supplementary Figure S1A). Further analysis with heatmap and two-way hierarchical clustering showed two distinct signatures, one for the tissue type (BAT versus WAT) and one for the differentiation state between BAT and WAT (Supplementary Figure S1B). Overall, several secreted miRNAs were found to be upregulated during brown differentiation when compared to WAT differentiation (Fig. 1G and H). The most prominent secreted miRNA that exhibited the highest log fold change during the pairwise comparison of undifferentiated BAT vs. WAT and differentiated BAT vs. WAT was miR-10b-5p.

Furthermore, miR-706 and miR-709 were also identified amongst the significantly differentially secreted miRNAs during differentiation of BAT and WAT (Fig. 1I). Thus, we identified three miRNAs (miR-10b-5p, miR-706 and miR-709) as potentially involved in brown and white adipogenesis. Validation of these miRNAs identified from miRNA profiling arrays and RNA sequencing was performed by quantitative real-time PCR using locked nucleic acid (LNA) detection primers. An upward trend in miR-709 expression was observed from day 6, whereas miR-10b-5p and miR-706 were significantly upregulated during a BAT differentiation time course (Fig. 1J).

As an assessment of BAT differentiation, we also analysed the expression of Fabp4 and Ucp1 (Fig. 1J). As expected, we observed significantly higher expression of key thermogenesis marker Ucp1 and adipogenesis marker Fabp4 during the later stages of BAT differentiation. To further extend these findings to human adipose biology, a time-course analysis of human brown adipocyte differentiation was performed. Adipogenic and thermogenic markers, including CIDEC, CIDEA, FABP4, and UCP1, were upregulated during differentiation (Fig. 1K). Notably, miR-10b-5p expression increased in parallel with these markers, displaying dynamic regulation throughout the differentiation process, suggesting a potential role in human brown adipogenesis.

### miR-10b is upregulated during ESCs differentiation to mature adipocytes

**Fig. 2:**
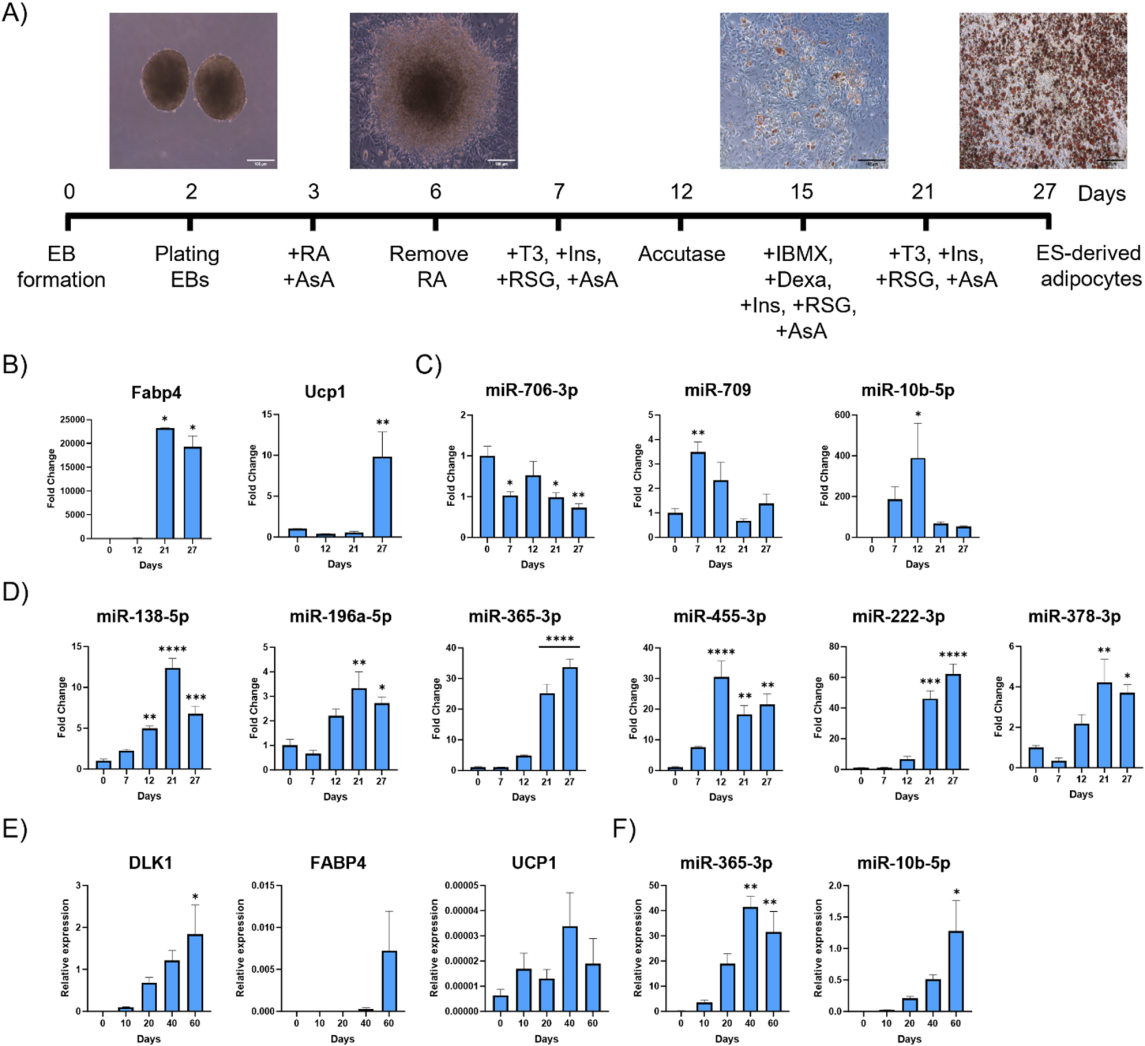
Adipogenesis markers and miRNA expression during ESCs differentiation to mature adipocytes. **A** Stepwise adipocyte differentiation protocol from mouse embryonic stem cells. Protocol adapted from [18]. **B** Fabp4 and Ucp1 expression was elevated during ESCs differentiation. **C** RT-qPCR analysis of miRNAs previously shown to regulate adipogenesis. **D** The expression of miR-706, miR-709 and miR-10b-5p was determined by RT-qPCR. **E** Expression levels of DLK1, FABP4, and UCP1, and (**F**) miR-365 and miR10b-5p, monitored during the differentiation process of hPSCs into BAs. mRNA and miRNA expression was normalized to the housekeeping gene L19 and U6 respectively. Data from Fabp4 (BA), miR-222 (BA), miR-709 (BA) and DLK1 (hESCs) analyses were evaluated using the Kruskal-Wallis test, while the remaining gene datasets were analysed using one-way ANOVA. Each day’s mean is compared to the mean of day 0, with significance assessed for each comparison. Data represent the mean ± SEM (*p < 0.05, **p < 0.005, ***p < 0.0005, ****p < 0.0001)

A large part of our knowledge of the molecular pathways governing adipogenesis has been acquired using cellular model systems such as immortalized murine cell lines (3T3-L1, 3T3-F442A, ob1771 and OP9), multipotent murine embryonic cell line C3H10T1/2, and primary culture models (SVF of fat depots) [38]. These models primarily focus on the late stages of the differentiation program. Thus, the sequence of events involved in the commitment of adipose tissue stem cells/precursors to preadipocytes remains largely unexplored. To study the involvement of the chosen miRNAs during the early stages of preadipocyte commitment, we exposed mESCs to an optimised protocol [18] to induce their differentiation to terminally differentiated adipocytes (Fig. 2A). The treatment included T3 and Rosi which, in addition to promoting adipogenesis, can contribute to the expression of brown fat genes. RT-qPCR analysis at various time points during differentiation confirmed a significant upregulation of adipocyte markers Fabp4 and Ucp1 (Fig. 2B). miR-10b-5p showed a dynamic expression profile during the time course (Fig. 2C). A nearly 389-fold increase of miR-10b-5p was observed during pre-adipocyte development on day 12 compared to day 0.

The expression of miR-706 and miR-709 did not appear to alter significantly during ES differentiation (Fig. 2C). miRNAs previously shown to regulate adipogenesis, including miR-138-5p [39, 40], miR-196a-5p [41], miR-365-3p [42, 43], miR-455-3p [44], miR-222-3p [45] and miR-378a-3p [46–48] were markedly elevated with differentiation (Fig. 2D). We hypothesized that induction of miR-10b-5p is a critical event for adipocyte development.

The stepwise differentiation and functional characterization of paraxial mesoderm cultures towards an adipogenic fate has recently been modelled in vitro using human embryonic stem cells (hESCs) [49]. To study the expression of miR-10b-5p in human adipocyte differentiation, human pluripotent stem cells (hPSCs) were differentiated into BAs. RNA samples were collected at different time points for RT-qPCR analysis. Induced pluripotent stem cell (iPSC) differentiation to fully mature BAs was confirmed by the upregulation of preadipocyte and brown adipogenic markers, including DLK1, FABP4 and UCP1 (Fig. 2E). BA development was also corroborated by the upregulation of a known adipogenic miRNA, miR-365-3p [42, 43] (Fig. 2F). In addition, miR-10b-5p expression steadily increased during iPSC adipocyte differentiation (Fig. 2F), supporting that miR-10b-5p might regulate human BA differentiation.

Taken together, miR-10b-5p is expressed and regulated across various cellular models of brown adipogenesis in mice and humans. These findings suggest that miR-10b-5p may be essential not only to the formation and differentiation of brown adipose tissue but also to the overall regulation of metabolic processes.

### CRISPR/CAS9 knockout of miR-10b

**Fig. 3:**
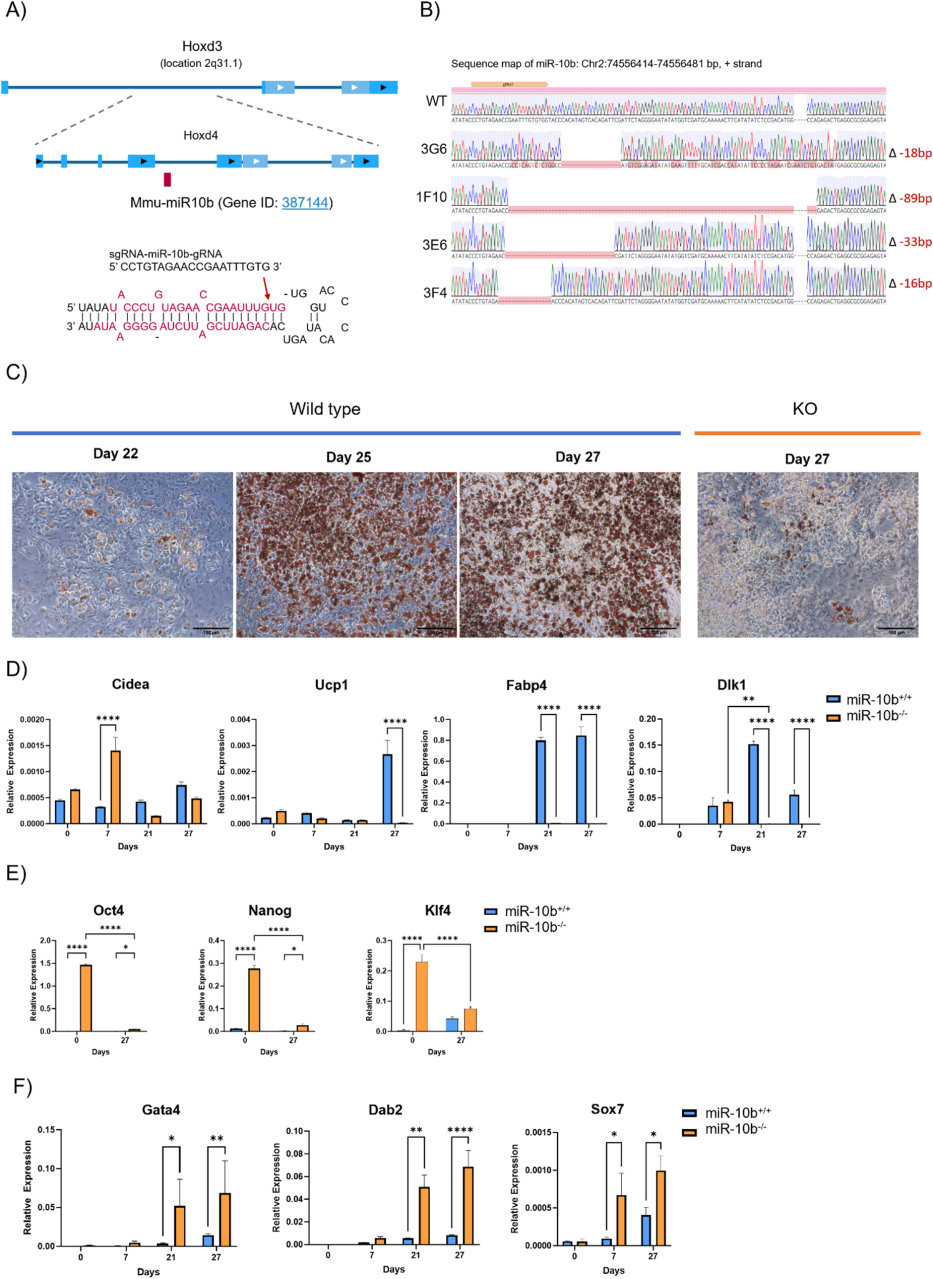
The loss of miR-10b significantly compromised adipogenesis. **A** Design of sgRNAs flanking the miR-10b precursor sequence located in the intronic region of Hoxd3 and Hoxd4. **B** Detection and confirmation of indels generated by CRISPR/cas9 editing. DNA sequencing confirms the deletions (Red) by CRISPR/cas9. **C** The degree of differentiation was evaluated by visualizing the amount of lipid accumulation on day 22, 25 and 27 of differentiation. A representative bright-field image from wild type and miR-10b KO cultures depicting the progress of differentiation. Lipid droplets were stained with Oil-Red-O staining. Scale bar represents 100 μm. **D** mRNA expression of the adipose-specific markers Cidea, Ucp1, Fabp4 and preadipocyte marker Dlk1 was measured by RT-qPCR. **E** Stem cell markers Oct4, Nanog and Klf4 (**F**) specific endoderm markers, Gata4, Dab2 and Sox7 were upregulated in KO clones. Data (*n* ≥ 3) are shown as means + SEM (two-way ANOVA test, *p < 0.05, **p < 0.005, ***p < 0.0005, ****p < 0.0001).

Because miR-10b is potentially relevant to the development of mature adipocytes, we sought to determine its specific role during adipogenesis by generating its functional knockout (KO) in E14 mESCs using CRISPR/Cas9 engineering technology. In the mouse genome, miR-10b resides in the intronic region of two overlapping genes, Hoxd3 and Hoxd4 (Fig. 3A). Given the significance of Drosha and Dicer processing sites during the canonical pathway of miRNA biogenesis [50], we constructed the CRISPR/Cas9 vector to contain a single guide RNA (sgRNA) designed to target within/adjacent to these sites in miR-10b. We identified KO events in 15% of colonies (6 out of 40). Sanger sequencing confirmed CRISPR/Cas9-induced deletions and indels in four clones (Fig. 3B). Sequence alignment showed complete disruption of the terminal loop, Drosha, and Dicer sites in three KO clones (3G6, 1F10, 3E6), while the fourth clone (3F4) had only the Drosha site affected.

Using CCTop - CRISPR/Cas9 target online predictor [22, 51], we predicted the off-targets of miR-10b sgRNA (Supplementary Table 9). Most targets had four base mismatches in the seed region and were in exonic, intronic, and intergenic regions. We did not analyse gene expression because the targets were primarily intronic or intergenic, or due to previous research findings that the presence of four or more base mismatches eliminates the detectable SpCas9 cleavage in most loci [52, 53].

We compared doubling times of wild type (WT) and miR-10b KO cells to assess the impact on proliferation rates and found no significant difference (Supplementary Figure S2A). Alkaline Phosphatase (AP) activity was used to evaluate self-renewal capacity in the presence and absence of LIF. Both KO and WT cells formed more undifferentiated colonies with LIF, but KO cells, derived from a clonal population, showed a higher number of undifferentiated colonies than WT (Supplementary Figure S2B). Hoxd3 and Hoxd4 mRNA levels were measured to determine whether the CRISPR-mediated miR-10b loss-of-function mutation affected their expression. Hoxd4 expression was undetectable by RT-qPCR (data not shown), and Hoxd3 levels did not differ significantly between KO and NTC cells Supplementary Figure S2D).

### Knockout of miR-10b severely compromises adipocyte differentiation

Loss of miR-10b in all four KO clones significantly impaired the differentiation process into mature adipocytes. In depth analysis was performed on KO clone 1F10. There was a clear reduction of lipid accumulation as judged by Oil Red O staining in KO compared to WT cells on day 27 of the differentiation treatment (Fig. 3C). Expression of Fabp4 and Ucp1 was profoundly reduced in KO compared to WT cells on day 27, further supporting that adipogenesis was impaired in the absence of miR-10b (Fig. 3D). It is noteworthy that in the pluripotent state, there was elevated Ucp1 expression in miR-10b KO compared to WT cells that was lost during the commitment and differentiation process (Fig. 3D).

Remarkably, the transcript abundance of Preadipocyte factor 1 (Pref-1/Dlk1), a molecular gatekeeper of adipogenesis [54], was consistently higher in WT compared to KO at 21 and 27 days of differentiation (Fig. 3D). Furthermore, Dlk1 expression was elevated only on day 7 in the KO and then diminished, suggesting that the absence of miR-10b expression hindered the commitment stages and prevented the cells from differentiating into mature adipocytes. The levels of Cidea remained largely unchanged throughout the differentiation process, apart from day 7, where KO cells exhibited significantly elevated expression levels compared to WT cells (Fig. 3D).

Cellular senescence is a key contributor to impaired adipogenesis, leading to dysfunctional adipose tissue formation and function [55, 56]. To investigate whether cells entered senescence due to depleted miR-10b, we stained mature adipocytes for β-galactosidase and observed them at ×20 magnification. Microscopic examination revealed that KO cells did not display signs of senescence, as indicated by the absence of cells staining positive for β-galactosidase activity (Supplementary Figure S2C).

In agreement with the commitment steps being hindered in the absence of miR-10b, the expression levels of three major pluripotent genes, Nanog, Oct4, and Klf4, were highly elevated in KO cells on Day 0 and Day 27 compared to WT cells (Fig. 3E). Moreover, expression of endoderm markers (Gata4, Dab2, and Sox7) was enriched in KO cells compared to WT cells (Fig. 3F).

### Impact of miR-10b depletion on stem cell commitment and lineage determination

#### Transcriptomic profiling identifies candidate downstream targets of miR-10b

**Fig. 4:**
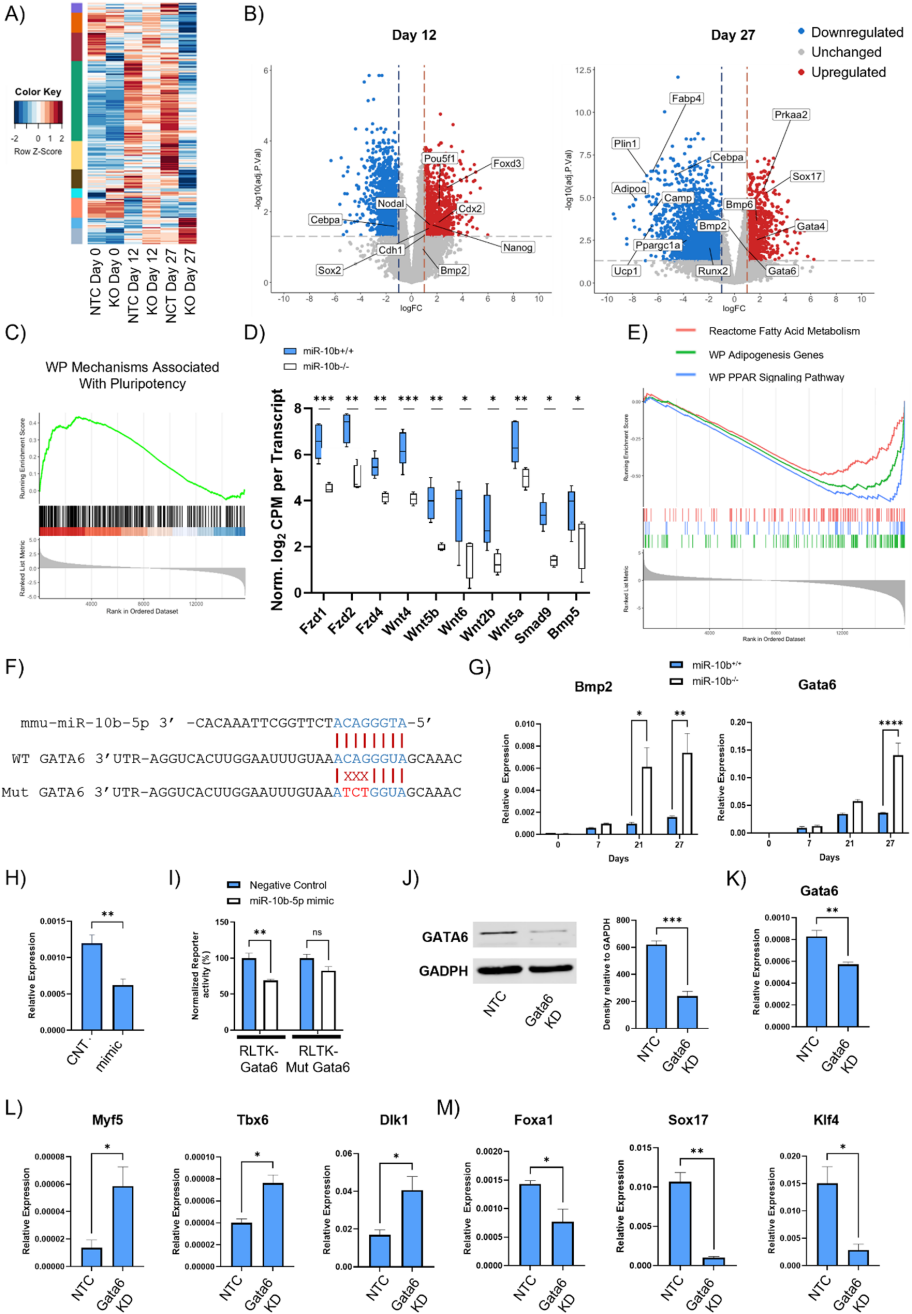
Transcriptomic and functional analyses of mESCs with or without miR-10b depletion during adipogenic differentiation, identifying Gata6 as a downstream target involved in lineage commitment. **A** Heatmap displays the results of hierarchical cluster analysis applied to 2304 commonly DEGs observed between the different cell phenotypes during the time points of day 0, 12 and 27. These DEGs were identified based on a log_2_ fold change greater than 2 and adjusted p-values < 0.05. **B** Volcano plots depicting the expression profile of genes in mESCs treated with gRNA targeting miR-10b or NTC vector and differentiated on Day 12 and 27 (log_2_FC > 1 and adjusted p-value < 0.05). Positive log_2_FC (red) = upregulated in KO; negative log_2_FC (blue) = downregulated in mESCs treated with NTC vector. **C** GSEA on day 12 revealed significant enrichment of gene sets involved in the mechanisms associated with pluripotency following miR-10b depletion. **D** Normalized and log₂-transformed CPM for each transcript of Frizzled receptor family members and Wnt family genes, illustrating transcriptional activity in response to miR-10b depletion. Data were normalized using the TMM method via the calcNormFactors function in edgeR (v4.0.4). Adjusted p-values were calculated using the Benjamini–Hochberg method (*p < 0.05, **p < 0.005, ***p < 0.0005). Centrelines represent the medians; whiskers extend to the minimum and maximum values. **E** GSEA results from day 27 dataset revealed reduced enrichment of gene sets associated with Fatty Acid Metabolism, Adipogenesis Genes and PPAR Signaling Pathway in the KO group. **F** Binding seed sequence of miR-10b-5p with the 3’UTR of Gata6. The mutated seed sequence of Gata6-UTR was denoted in red. **G** The relative expression of Bmp2 and Gata6 mRNA during mESC differentiation to adipocytes. **H** The expression of Gata6 was analysed by RT-qPCR in KO cells in response to miR-10b mimic. **I** Luciferase reporter assay demonstrating the target relationship between miR-10b-5p and Gata6. The WT or mutant-type (MUT) constructs were inserted into the pRL-TK vector and firefly and Renilla luciferase activities were determined (*n* = 3, mean ± SEM. ANOVA, **p < 0.01, ns: not significant). **J** and K) siRNA-mediated depletion of Gata6 in miR-10b-/- mESCs reduced GATA6 protein and Gata6 mRNA levels. **L** and (**M**) miR-10b⁻/⁻ mESCs treated with Gata6 siRNA for 48 h and subjected to adipogenic differentiation exhibited increased expression of mesodermal markers (Myf5, Tbx6, and Dlk1) and decreased expression of endodermal lineage markers (Foxa1, Sox17, and Klf4) on day 11. Data were analysed with Student’s t-test and are presented as mean ± SEM (*n* ≥ 3), *p < 0.05, **p < 0.005, ***p < 0.0005)

To uncover the pathways influenced by miR-10b depletion, a transcriptomic analysis was conducted on samples collected on days 0, 12, and 27 (Supplementary Table 10). The PCA plot shows no clear segregation between the NTC and KO clones on day 0 (Supplementary Figure S3A). On Day 12, there is a clear separation of NTC and miR-10b KO samples, with further separation occurring by day 27 of differentiation, suggesting that the gene expression profiles of the two groups are distinctly different (Supplementary Figure S3A). The main factor affecting the variance we observe appears to be the differentiation status of the cells. Transcriptomic analysis revealed 2304 genes with significant differential expression during key stages of mESC differentiation to mature adipocytes across both phenotypes (log_2_ fold change > 2, adjusted p-values ≤ 0.05; Fig. 4A).

To assess the differentially expressed genes (DEGs) between the two groups through days 0, 12 and 27 of adipogenesis, we utilized criteria where the adjusted p-value was ≤ 0.05 and the absolute value of log_2_ fold change ≥ 1. In total, 499 genes showed increased expression, while 679 genes exhibited decreased expression on day 0 (Supplementary Figure S3B). On day 12, during the commitment stage, 898 genes were upregulated, and 1147 genes were downregulated. On day 27, during the late phase of differentiation, 820 genes were upregulated, and 1981 genes were downregulated (Fig. 4B). Importantly, RNA-seq analysis showed that Hoxd3 and Hoxd4 expression was unaffected by the CRISPR-mediated miR-10b loss-of-function mutation, with both genes exhibiting very low transcript counts consistent with the RT-qPCR results on day 0 (Supplementary Figure S13E, Figure S2D).

Gene set enrichment analysis (GSEA) of the day 12 dataset demonstrated a strong enrichment of gene sets associated with mechanisms related to pluripotency (Fig. 4C). Notably, Nanog, a homeodomain protein that is transcribed exclusively in pluripotent cells within ESCs, along with its transcription activators Pou5f1 (Oct4), Sox2 and Foxd3 [57] were upregulated in the miR-10b KO cells compared to control samples (Fig. 4B). Moreover, various genes encoding members of the Frizzled receptor family (Fzd2, Fzd1, and Fzd4), which are involved in the WNT signaling pathway as well as members of the Wnt family (Wnt4, Wnt5b, Wnt6, Wnt2b, and Wnt5a) were shown to be downregulated in the KO group (Fig. 4D).

Suppression of these genes may decrease the activation of the WNT pathway [58, 59], leading to a possible alteration in cell fate decisions and favouring the endodermal lineage.

Furthermore, genes involved in bone morphogenetic protein (BMP) signaling such as Smad9 and Bmp5 [60, 61], were also downregulated in the KO group, which could lead to reduction of BMP signaling (Fig. 4D), which is crucial for mesodermal commitment [62]. These observations are consistent with the results indicating an elevation in the expression of specific markers associated with the endodermal lineage during adipogenesis treatment of KO mESCs (Fig. 3F).

At the later stages of differentiation (day 27), transcriptomic analysis revealed an increase in the expression of adipogenic markers, including Fabp4, Plin1, Adipoq, Cebpa, Ucp1, Ppargc1a, and Runx2 in the NTC clones (Fig. 4B). This collective upregulation supports various aspects of adipocyte differentiation, lipid metabolism, and adipose tissue function, thereby promoting the adipogenic process. Consistent with the finding of suppressed terminal stem cell differentiation in miR-10b KO cells, GSEA on day 27 revealed that key signaling pathways associated with fatty acid metabolism, adipogenesis genes and PPAR signaling pathway were suppressed in the KO group (Fig. 4E). Interestingly, the preference for the endodermal lineage observed in the KO group on day 12 persisted until day 27, as evidenced by the increased expression levels of Sox17, Bmp2, and Gata6 (Fig. 4B). Taken together, these findings demonstrate that the loss of miR-10b appears to play a role in cell fate determination during embryonic development, leading to reduced mesodermal commitment and a bias toward endodermal lineage specification.

#### Gata6 is a direct target of miR-10b-5p during lineage commitment

TargetScan was utilized to predict potential targets of miR-10b-5p to investigate the molecular process through which miR-10b-5p governs adipogenesis. Among the genes suppressed by miR-10b-5p, Gata6, an endoderm related gene [63], was predicted to be a direct target through its 3′ UTR (Fig. 4F). We initially assessed the mRNA expression levels of Gata6 and its downstream target Bmp2 during adipogenesis [63]. We observed that the expression pattern of both these genes was upregulated in response to miR-10b deletion (Fig. 4G). To investigate if miR-10b-5p directly targets Gata6, we treated miR-10b KO cells with a miR-10b-5p mimic or a NTC miRNA mimic. Analysis by RT-qPCR showed that overexpression of miR-10b-5p downregulated the expression of Gata6 (Fig. 4H).

We generated luciferase reporters that had either a wild-type (WT) Gata6 3’UTR or a 3’UTR-containing mutated (MUT) miR-10b-5p binding site (Fig. 4F). Dual luciferase reporter analysis in HEK293 cells demonstrated that co-transfection of miR-10b-5p significantly suppressed reporter activity of the WT Gata6 3’UTR, but not with the mutated binding site (Fig. 4I), further supporting that Gata6 is a direct target of miR-10b-5p. To investigate whether miR-10b-5p–mediated regulation of Gata6 influences lineage commitment between endodermal and mesodermal fates, miR-10b KO cells were transfected with Gata6 siRNA and differentiated them to day 11. This time point was selected to capture early commitment events coinciding with the induction of the preadipocyte marker Dlk1, which has been found to be upregulated from day 7 onwards (Fig. 3D). Efficient siRNA-mediated KD of Gata6 in miR-10b KO cells was confirmed by a significant reduction in both Gata6 mRNA and GATA6 protein levels (Fig. 4J and K). KD of Gata6 at the onset of differentiation markedly increased the expression of Myf5, Tbx6, and Dlk1, indicating enhanced paraxial mesoderm specification and commitment (Fig. 4L). Consistent with this shift in lineage bias, day-11 cultures exhibited reduced expression of endodermal-associated markers, including Foxa1, Sox17, and Klf4 (Fig. 4M). These findings support a model in which miR-10b-5p plays a central role in regulating stem cell fate decisions, with Gata6 acting as a potential downstream effector that may contribute to restraint of paraxial mesoderm specification and limitation of brown adipogenic commitment.

### Ablation of miR-10b-5p in brown adipocytes severely impairs differentiation

**Fig. 5:**
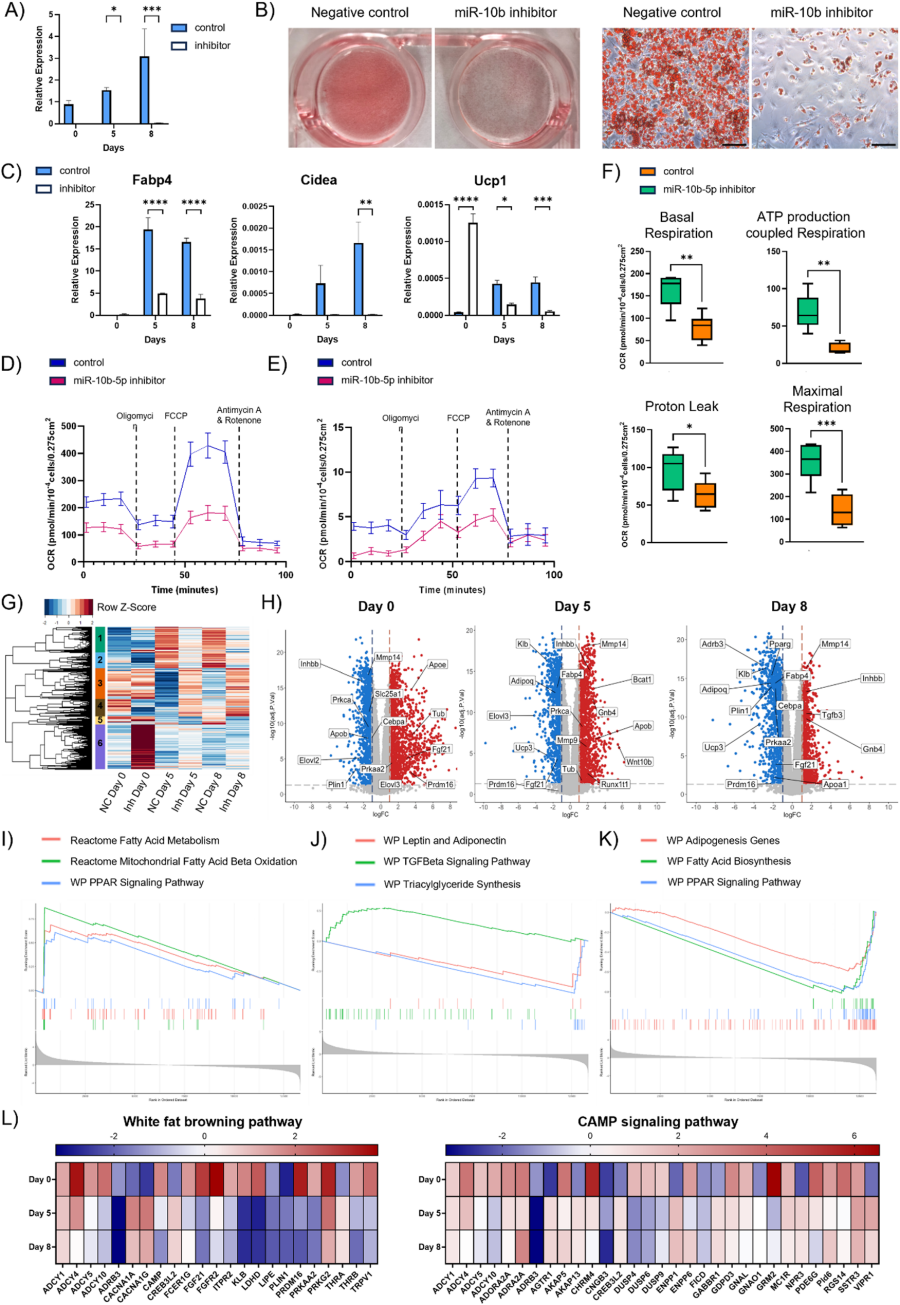
Downregulation of miR-10b severely compromised brown adipogenesis. **A** Detectable miR-10b-5p signal is markedly reduced following miR-10b-5p inhibitor treatment. **B** Representative Oil-Red-O staining of differentiated brown adipocyte cultures treated with miR-10b-5p inhibitor or negative control (NC). Scale bar: 50 μm. **C** RT-qPCR analysis of gene expression of adipogenic and brown fat-selective genes in brown adipocytes. **D** and (E) Impact of miR-10b’s downregulation on cellular bioenergetics. ECAR and OCR were measured eight days after transfection (n ≥ 5). Blue line: NC, pink line: miR-10b-5p inhibitor. **F** Box and whisker plots showing the components of mitochondrial respiration changes in differentiated BA, ATP production coupled respiration, proton leak and maximal respiration capacity. Centrelines indicate the medians and whiskers extend to the minimum and maximum values. (Student’s t-test, n ≥ 5, *p < 0.05, **p < 0.005, ***p < 0.0005, ****p < 0.0001). **G** Transcriptomic analysis of brown adipocytes treated with miR-10b inhibitor or NC during differentiation. Heatmap showing the hierarchical cluster analysis of 1601 common DEGs between the treatment groups across the different time points of BA differentiation (log2FC > 2 and adjusted p-value < 0.05). **H** Volcano plots illustrating the expression profile of genes in BAs treated with miR-10b inhibitor (Inh) or NC on Day 0, 5 and 8 (log2FC > 1 and p value < 0.05). Positive logFC (red) = upregulated in BAs treated with miR-10b inhibitor; negative logFC (blue) = downregulated in BAs treated with NC. **I** GSEA revealed a significant enrichment of genes associated with the Mitochondrial Fatty Acid Beta Oxidation, Fatty acid Metabolism and PPAR signaling among genes upregulated following miR-10b-5p inhibition on day 0. **J** GSEA on day 5 revealed reduced enrichment of gene sets related to Triacylglyceride Synthesis as well as Leptin and Adiponectin signaling following miR-10b-5p inhibition. **K** GSEA results from the day 8 dataset also revealed downregulation of gene sets associated with adipogenesis, PPAR signaling, and fatty acid biosynthesis. **L** Heatmaps illustrating gene expression patterns associated with the White Fat Browning Pathway and cAMP Signaling Pathway identified through GSEA on day 0,5 and 8.

To further delineate the potential functions of miR-10b-5p during adipogenesis, we investigated if loss of function of this miRNA also influenced the later stages of differentiation. To this end, immortalized brown preadipocytes were treated with miRCURY miRNA Inhibitor that silenced miR-10b-5p and subjected cells to standard brown adipogenic treatment. Cells were also treated with a negative control that did not exhibit homology to any mouse sequence in the NCBI and miRBase databases. Efficient delivery of the miR-10b-5p inhibitor into brown preadipocytes was initially assessed by RT-qPCR, which revealed a marked reduction in detectable miR-10b-5p signal (> 100-fold) compared with control cells. This reduction was maintained throughout differentiation until day 8 (Fig. 5A). miR-10b-5p KD in brown preadipocytes significantly suppressed brown adipogenesis as judged by reduction of Oil Red O Staining compared to cells treated with negative control (Fig. 5B). This was also evidenced by the decreased expression of adipogenic and brown fat-selective genes (Fig. 5C). Interestingly, Ucp1 expression was transiently upregulated prior to the treatment of standard adipogenic induction on day 0, reproducing the observation in miR10b-5p knockout ESCs.

To examine the consequence of downregulation of miR-10b-5p on bioenergetics, OCR and extracellular acidification rate (ECAR) in differentiated adipocytes was measured by Seahorse assay on day 8 (Fig. 5D-F). miR-10b-5p loss of function decreased basal respiration, respiration used for ATP production, mitochondrial respiration, proton leak and maximal respiratory capacity. Basal ECAR was also reduced by inhibiting miR-10b-5p both before and after oligomycin treatment, and when analysed against OCR. The inhibition of miR-10b-5p reduced glycolysis and oxidative phosphorylation, which is representative of fewer metabolically active cells. Collectively, these data suggest that reducing miR-10b-5p levels alters the mitochondrial metabolic profile.

### Dynamic impact of miR-10b inhibition on molecular processes that govern different stages of brown adipogenic differentiation

To unravel the pathways that are affected by miR-10b-5p depletion, a transcriptomic analysis was performed on days 0, 5, and 8 of BA differentiation in the absence and presence of miR-10b-5p inhibitor. The PCA plot clearly indicated that the transcriptomic profiles of the six sample groups were distinctly different (Supplementary Figure S4). The primary factor influencing the observed variance was the differentiation status of the cells. Furthermore, the plot also illustrated that the largest changes occur between days 0 and 5 or 8 in the cells subjected to the negative control.

Transcriptomic analysis identified 1601 differentially expressed genes at key time points of brown adipogenesis in the two treatment groups based on log_2_ fold change of ≥ 2 and adjusted p-values ≤ 0.05 (Fig. 5G).

To compare the DEGs between the miR-10b-5p inhibitor and control groups across the selected time points of differentiation, the adjusted p value ≤ 0.05 and the absolute value of log_2_ (fold change) ≥ 1 were employed as criteria. In total, 1730 genes were upregulated, and 1303 genes were downregulated on day 0, 1384 genes were upregulated, and 1143 genes were downregulated on day 5, whereas 956 genes were upregulated and 1318 downregulated in the late phase of BA differentiation (day 8) (Fig. 5H). Specific genes associated with adipogenic differentiation (Apoe); thermogenic program (Fgf21, Prdm16) and lipid metabolism (Elovl3) were increased on day 0.

In contrast, genes related to the same pathways were downregulated on day 0, including Cebpa, Prkca and Inhbb (Fig. 5H). Additionally, a Gene Ontology (GO) analysis was conducted on cluster module 6 of the heatmap (Fig. 5G), identified as the most densely populated module with the highest upregulated genes in cells treated with the miR-10b-5p inhibitor compared to the negative control. Enrichment analysis showed that several enriched GO terms were related to cell development and differentiation, as well as genes associated with lipid metabolism. Additionally, GO analysis revealed enriched transcription factors, including Pparg, Smad3, and Srebf1 (Supplementary Figure S5).

GSEA of the day 0 dataset revealed a striking enrichment of gene sets involved in energy metabolism, lipid metabolism, and mitochondrial function, specifically within the BAs treated with miR-10b-5p inhibitor (Supplementary Figure S6). The heightened expression of gene sets associated with Mitochondrial Fatty Acid Beta Oxidation, Fatty acid Metabolism and PPAR signaling suggests a role for miR-10b-5p-dependent inhibition of metabolic processes critical for thermogenesis and fat metabolism in the undifferentiated state (Fig. 5I, Supplementary Figure S6). We also observed a substantial enrichment of genes associated with the white fat browning pathway and the cAMP signaling pathway in BAs with miR-10b-5p inhibition on day 0 compared to the other days (Fig. 5L). These results demonstrate that key genes related to the brown adipogenic program are activated upon miR-10b-5p inhibition on day 0. However, upon induction of the adipogenic differentiation program and during the late phase of differentiation, the majority of those key genes display markedly reduced expression with miR-10b-5p inhibition. Several of these biological pathways overlap between the two datasets on day 5 and day 8 (Supplementary Figures S7 and S8). Upon miR-10b-5p KD, biological pathways associated with lipid droplet formation, including those involved in triacylglycerol synthesis, fatty acid metabolism, and lipid metabolism, were suppressed during brown adipogenic differentiation. Notably, key signaling pathways related to the function and development of BAs were also repressed, such as oxidative phosphorylation, PPAR Signaling Pathway, and leptin and adiponectin pathways (Fig. 5J, K). Consistent with the finding of perturbed differentiation, GSEA also revealed activation of the TGF-beta signaling pathway (Fig. 5J), a pathway previously reported to impede preadipocyte differentiation [64, 65].

Gata6 levels were upregulated during mouse and human BA differentiation (Supplementary Figure S13A, B). TPM-based analysis showed largely comparable Gata6 transcript levels between control and miR-10b-5p KD cells during differentiation, although a significant increase was observed in KD cells on day 8 (Supplementary Figure S13D). Given the minimal and late-stage difference in Gata6 expression, these data suggest that Gata6 is unlikely to be the primary mediator of miR-10b-5p–dependent effects during the later stages of brown adipocyte differentiation, raising the possibility that other miR-10b-5p targets contribute to this process.

### Tub abundance is regulated by miR-10b-5p

**Fig. 6:**
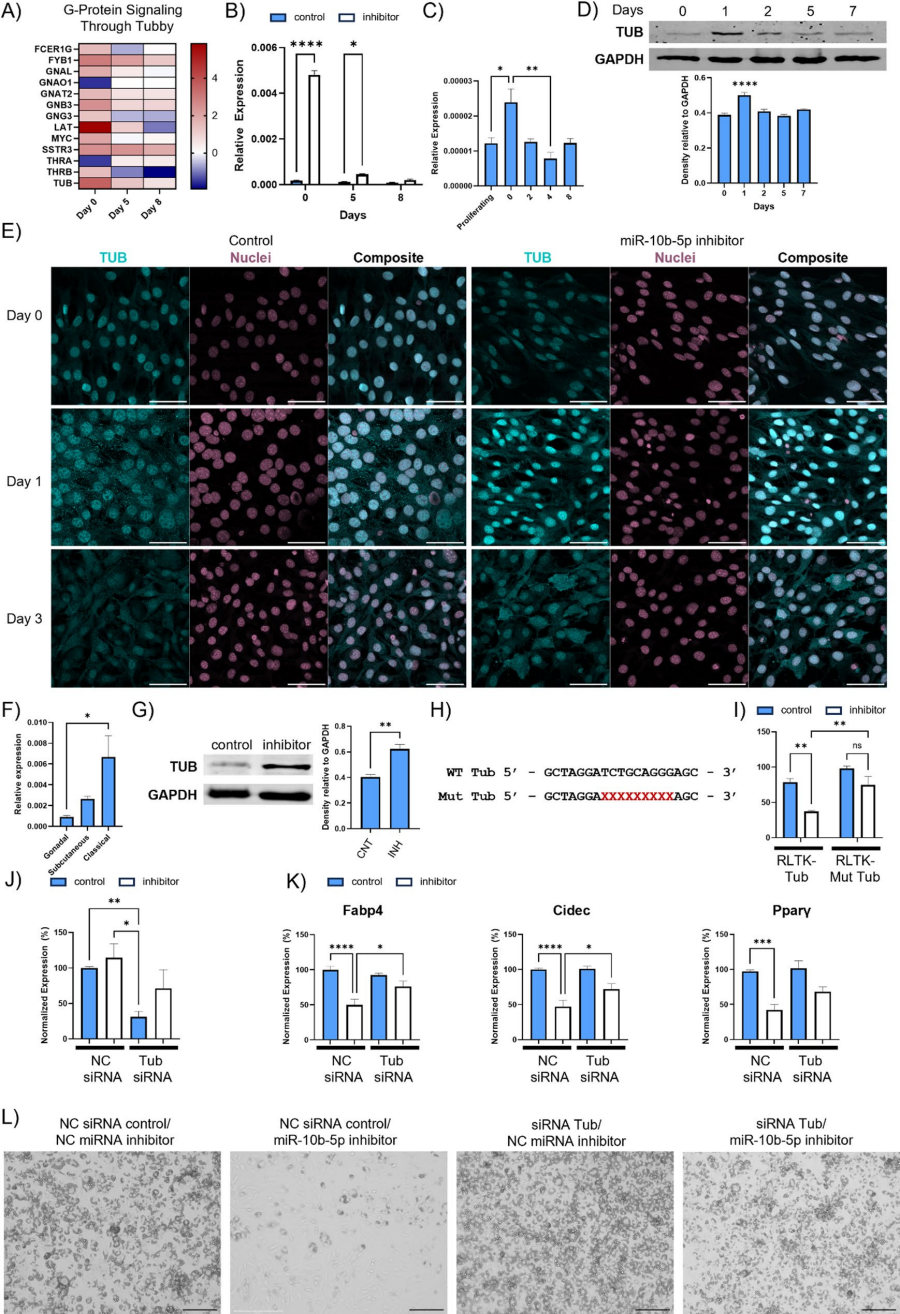
The expression level of Tub is negatively correlated with miR-10b-5p abundance during BA differentiation. **A** Heatmap depicting gene expression patterns linked to G-protein Signaling through Tubby identified through GSEA on day 0,5 and 8 of BA differentiation. **B** RT-qPCR analysis of Tub expression in response to miR-10b-5p inhibitor treatment at specific time points of BA differentiation. **C** Time-course RT–qPCR analysis of Tub mRNA levels during BA differentiation (two-way ANOVA, *n* ≥ 4; *p < 0.05, **p < 0.005). **D** Time-course analysis of TUB abundance during brown adipogenesis shows dynamic regulation of TUB levels across differentiation relative to day 0 (2-way ANOVA, *n* = 4, ****p < 0.0001). **E** Tub immunostaining during BAT differentiation. Scale bar: 50 μm. Images taken at 40x magnification. (NC: Negative control, 10b inh: miR-10b-5p inhibitor). **F** Measurement of TUB expression in three different adipose tissue depots: adipocyte-fraction from gonadal WAT, subcutaneous WAT and classical BAT. mRNA levels were standardised against those for the L19. (2-way ANOVA, *n* = 3, *p < 0.05). **G** Western blotting analysis of brown preadipocytes treated with a miR-10b inhibitor or negative control and differentiated for 1 day, at which time TUB protein levels were assessed. (Student’s t-test, *n* = 4, **p < 0.005). CNT: Negative Control, INH: miR-10b-5p inhibitor. **H** Binding seed sequence of miR-10b with the 3’UTR of Tub. The mutated seed sequence of Tub-UTR was denoted in red. **I** Luciferase reporter assay demonstrating the target relationship between miR-10b-5p and Tub (*n* ≥ 3, mean ± SEM. 2-way ANOVA, *P < 0.05, **P < 0.005). **J** Tub expression levels in mature brown adipocytes following transfection with negative control or Tub-targeting siRNA in combination with miR-10b-5p inhibitor or corresponding control inhibitor prior to differentiation (Kruskal–Wallis test, *n* ≥ 8; *p < 0.05, ***p < 0.0005). **K** RT–qPCR analysis of adipogenic marker genes (Fabp4, Cidec, and Pparγ) in differentiated brown adipocytes treated as in (**J**). Fabp4 and Cidec were analysed by two-way ANOVA (*n* ≥ 8; *p < 0.05, ****p < 0.0001), and Pparγ by Kruskal–Wallis test (*n* ≥ 12; ***p < 0.0005). **L** Representative bright-field images showing lipid droplet accumulation in brown adipocytes on day 6 of differentiation. Scale bar, 100 μm; magnification, 10×. NC, negative control

In silico prediction [66] identified multiple hits for miR-10b-5p targeting of Tub, a transcription regulator that transmits signals from G protein-coupled receptors (GPCRs) and G proteins [67]. In our analysis of whole-transcriptome RNA sequencing during BA differentiation time course, we noted a significant increase in genes related to G Protein signaling through Tubby (Fig. 6A). In agreement with the transcriptomic analysis, we found that Tub mRNA (Fig. 6B) increased in response to miR-10b-5p inhibitor transfection in BAs. To define the temporal and spatial expression of Tub during adipocyte differentiation, Tub mRNA and protein levels were assessed in BA cells at key time points. Tub mRNA levels were highest in confluent preadipocytes and at the onset of differentiation (day 0), whereas Tub protein levels peaked on day 1 and then gradually declined as differentiation progressed (Fig. 6C and D). Notably, this dynamic expression pattern of Tub was inversely correlated with miR-10b-5p expression during BA differentiation.

Consistent with these temporal changes, Tub protein, determined through immunocytochemistry, exhibited a distinct spatiotemporal localization pattern during brown adipogenesis. On Day 0, Tub protein was primarily concentrated in the nuclei. By day 1, BAs displayed a uniform increase in Tub protein throughout the cell, with a fine punctate pattern distributed intracellularly. By Day 3 of differentiation, this punctate pattern was markedly reduced (Fig. 6E). To assess Tub expression across adipose depots and cellular fractions, Tub expression was quantified in different adipose depots and cellular fractions. Tub mRNA expression was significantly higher in classical BAT compared to gonadal and subcutaneous WAT within the mature adipocyte fraction (Fig. 6F). Across depots, Tub mRNA levels were consistently higher in the stromal vascular fraction than in the adipocyte fraction, while preserving the tissue-specific expression pattern (Supplementary Figure S13C). Together, these data indicate that Tub exhibits a distinct spatiotemporal expression pattern during brown adipogenesis.

### 10 Tub is a direct target of miR-10b-5p

To investigate a potential direct interaction between miR-10b-5p and Tub, brown preadipocytes were treated with a miR-10b inhibitor or negative control and differentiated for 1 day, leading to a significant increase in TUB protein levels in the presence of the inhibitor (Fig. 6G). To investigate whether miR-10b-5p can directly target Tub mRNA we cloned a section of the 3′UTR segment of Tub containing the predicted miR-10 b-5p target site or with the seed site mutated (Fig. 6H) into the RLTK luciferase reporter construct. The reporter constructs were co-transfected into HEK293 cells with either miR-10b-5p or negative control mimics. Luciferase reporter assays showed that, unlike the negative control, miR-10b-5p mimics reduced the activity of the reporter construct harbouring a WT 3′UTR (Fig. 6I). In contrast, mutations in the seed sequence prevented miR-10b-5p-dependent suppression of reporter gene activity. This shows that miR-10b-5p directly interacts with the predicted target sites in the Tub transcript.

To further investigate this finding, BAs were transfected with miR-10b-5p inhibitor in the presence or absence of Tub siRNA and then subjected to the standard differentiation protocol. Tub siRNA decreased Tub protein and mRNA expression, although levels were higher in cells co-transfected with miR-10b-5p inhibitor (Fig. 6J). This indicates that inhibiting miR-10b-5p can counter the action of siRNA on Tub expression at the later stages of differentiation. To investigate the suppression of BA differentiation, we monitored expression of key genes associated with adipogenesis and examined morphological changes in cells exposed to combinations of miR-10 b-5p inhibitor and Tub siRNA. Tub KD partially reversed the miR-10b-mediated decrease in Fabp4, Cidec and Pparg upon differentiation (Fig. 6K), indicating that the increase in Tub expression due to miR-10b-5p inhibition appears to be essential for the observed effects. This was further evidenced by the decreased lipid droplet formation in cells treated with the miR-10b-5p inhibitor on day 6, being mitigated by co-transfection with Tub siRNA Tub (Fig. 6L, Supplementary Figure S9).

### 11 Increased abundance of miR-10b-5p promotes the thermogenic program in white adipose tissue

**Fig. 7:**
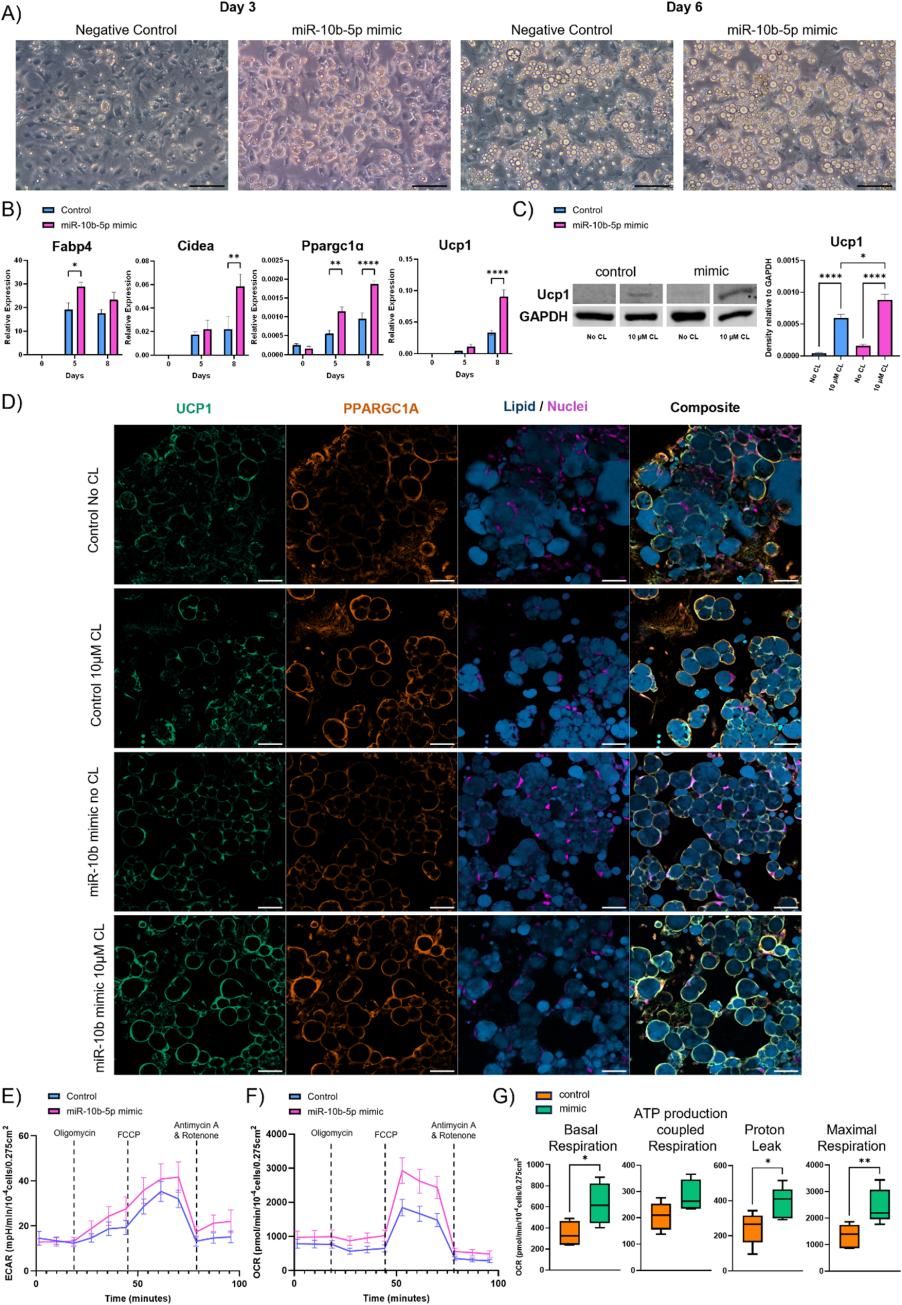
Elevated miR-10b-5p levels in white adipocytes promote adipogenesis. **A** Differentiation efficiency was assessed by observing the level of lipid accumulation on days 3 and 6. A representative bright-field image was captured from cultures treated with either negative control or mimic, depicting the progress of differentiation. **B** RT-qPCR analysis of adipogenic and brown fat-selective genes during WA differentiation following miR-10b-5p mimic treatment (2-way ANOVA, *n* = 4, *p < 0.05, **p < 0.005, ***p < 0.0005, ****p < 0.0001). **C** and (**D**) On day 8, cells were treated with 10 µM CL 316,243 (CL) or DMSO (control) for 24 h. UCP1 expression was assessed by Western blotting and immunocytochemistry, while PGC1α was analysed by immunocytochemistry. Upon activation of β-adrenergic signaling, WAs with elevated miR-10b-5p levels exhibited a significant upregulation of UCP1 (2-way ANOVA, *n* ≥ 3, *p < 0.05, ****p < 0.0001) and Ppargc1ɑ. **E** Effects on Bioenergetics Triggered by overexpression of miR-10b-5p. Extracellular acidification rate (ECAR) and **F** oxygen consumption rate (OCR) were measured on day 8 (*n* ≥ 4). **G** Box and whisker plots depicting the components of mitochondrial respiration changes in differentiated WA, ATP production coupled respiration, proton leak and maximal respiration capacity. Centrelines indicate the medians and whiskers extend to the minimum and maximum values (Student’s t-test, *n* ≥ 5, *p < 0.05, **p < 0.01)

As miR-10b-5p was found to be significantly enriched in BAs in comparison to WAs, we investigated the effects of miR-10b-5p upregulation on WA differentiation. Immortalized white preadipocytes were transfected with hsa-miR-10b-5p miRCURY LNA miRNA mimic or negative control mimic and subjected to standard white adipogenic differentiation. miR-10b-5p mimic significantly enhanced white adipogenesis as judged by the increased production of lipid droplets in cells on day 3 and 6 compared to negative control (Fig. 7A). Interestingly, by day 8, the negative control group appeared to have reached similar levels of lipid droplet synthesis as the mimic group, as shown by staining for lipid droplets (Supplementary Figure S10). However, the mimic group appears to have larger lipid droplets compared to the negative control (Fig. 7A). The elevated expression of adipogenic marker Fabp4 further strengthened this observation. We also observed increased Ucp1, Cidea and Ppargc1a levels during white adipogenesis, indicating a possible increase in the expression of a thermogenic gene program (Fig. 7B). While increased levels of miR-10b-5p in WAs elevated their rate to undergo differentiation, we tested whether the cells also gained propensity for β‐adrenergic stimulation. Activation of the β3AR with CL led to a significant upregulation of UCP1 and PPARGC1A protein levels in cells with elevated miR-10b-5p levels (Fig. 7C and D). To determine whether functional changes in bioenergetics accompanied the observed thermogenic-induced effects of miR-10b-5p mimic, differentiated WA cultures were analysed using the Seahorse assay (Fig. 7E-G). The miR-10b-5p mimic during WA differentiation instigated a substantial increase in maximal respiration, signifying an enhanced capacity for oxygen consumption under conditions of elevated energy demand. While not statistically significant, the treatment appeared to enhance ATP production-coupled respiration, suggesting a potential improvement in the efficiency of ATP synthesis. Moreover, there was also a rise in proton leak, indicating a higher mitochondrial uncoupling, which could potentially contribute to thermogenesis. Collectively, these results highlight that the miR-10b-5p mimic improved various parameters of mitochondrial bioenergetics, potentially contributing to increased cellular energy metabolism and function. 

To gain more insight into Tub’s mechanisms during the process of white adipocyte differentiation, we stained white adipocytes at various stages of differentiation (Supplementary Figure S11). Tub expression was elevated during the early stages of differentiation, contrasting with reduced expression in later stages. Notably, on day 0, Tub exhibited strong nuclear localization, whereas on day 3 and 7, it was reduced in expression and more uniformly distributed throughout the cytoplasm.

Additionally, Tub protein displayed a punctate pattern on day 3, although to a lesser extent compared to BAs (Fig. 6E). We also studied Tub protein behaviour in response to miR-10b-5p mimic. The negative control exhibited uniform Tub staining throughout the cell, whereas miR-10b-5p overexpression led to a punctate pattern, hinting at a distinct cellular response. Fluorescence intensity remained consistent across treatments, yet variations in lipid droplet sizes were evident (Supplementary Figure S12). The elevated lipid droplet staining in mimic treated WAs on day 3 further confirm the promotion of adipogenesis by miR-10b-5p. Together, these findings emphasise Tub’s dynamic involvement in adipocyte differentiation, indicating intricate regulatory processes that warrant further investigation.

## Discussion

**Fig. 8:**
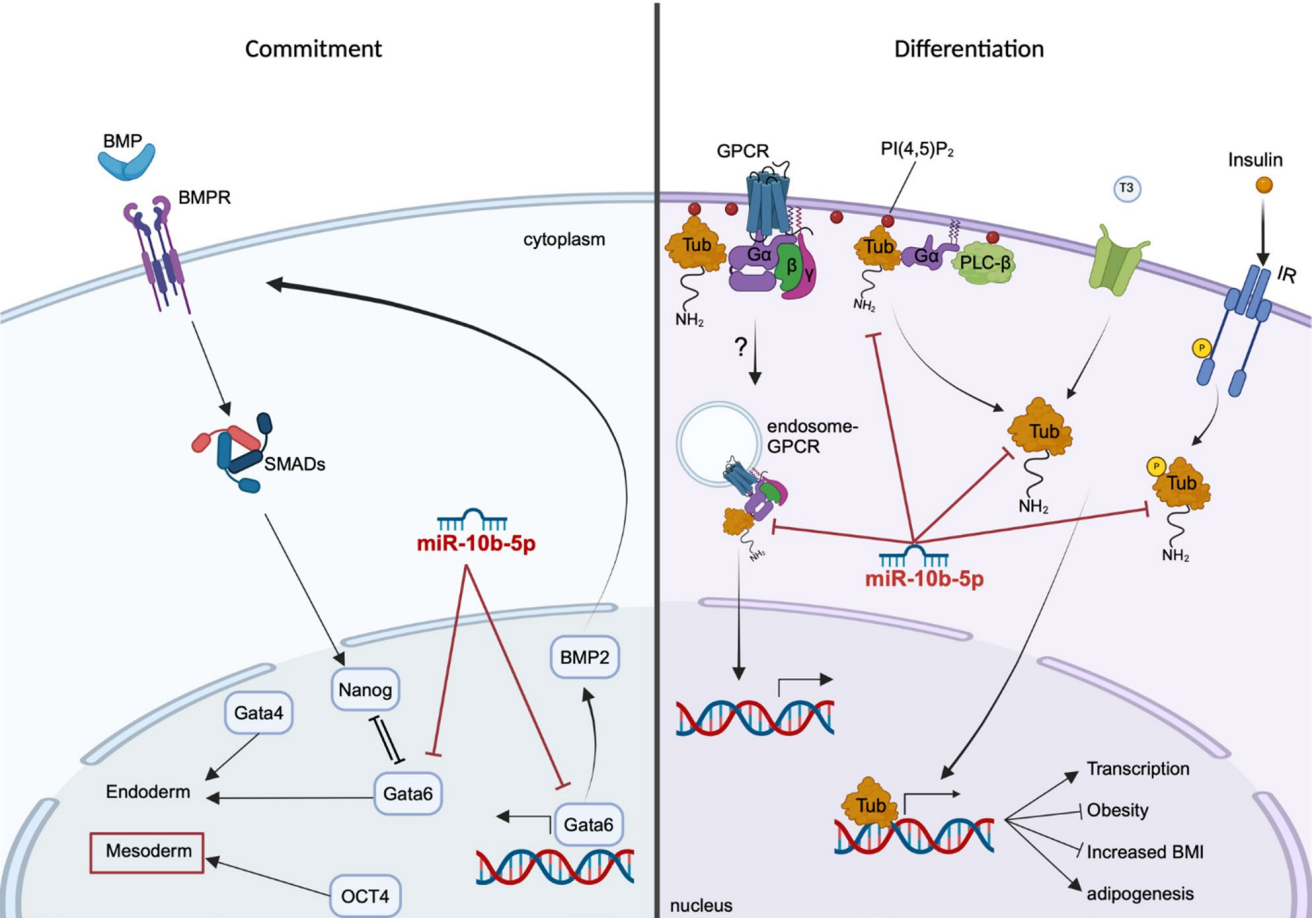
A Model of miR-10b-5p-mediated signaling influencing lineage commitment and adipogenesis. During stem cell differentiation, BMPs initiate signaling through binding to BMP receptors (BMPRs), leading to phosphorylation and activation of Smad1, Smad5, and Smad8. These activated SMADs translocate into the nucleus to regulate gene expression that promotes mesoderm and endoderm differentiation. In mESCs, Nanog binds Smad1 and blocks coactivator recruitment, limiting BMP signaling and mesodermal progression. Nanog also directly represses Gata6, which in turn downregulates Gata4, blocking transcriptional networks associated with primitive endoderm specification. Gata6 is required for cells to respond to lineage-inductive cues, which leads to downregulation of Nanog and progression toward a primitive endoderm fate. The balance between Nanog and Gata6 expression levels within individual cells serves as a key determinant in guiding the choice between embryonic and extraembryonic lineages. Additionally, Gata6 is a direct target of miR-10b-5p, and this post-transcriptional regulation may further influence the shift toward endodermal commitment rather than towards adipogenic lineage. Furthermore, miR-10b-5p was also identified as a positive regulator in the late stages of adipogenesis by directly regulating Tub expression. Tub expression is regulated by T3, and it functions as a membrane-bound transcription regulator that translocates to the nucleus upon phosphoinositide hydrolysis, thereby linking GPCR signaling to gene expression. Insulin induces its tyrosine phosphorylation and promotes its association with the insulin receptor

Adipogenesis is a highly complex process and is dynamic in nature. It involves the transition of preadipocytes into nondividing adipocytes and the key mechanisms that promote preadipocytes commitment and maturation includes multiple epigenetic factors, miRNAs as well as protein regulators. However, it is still unknown how and when an irreversible conversion occurs into a definitive state capable of accumulating lipids [68]. With the ongoing obesity epidemic and the strong associations of obesity with diabetes, cardiovascular disease and carcinogenesis [69, 70], understanding the molecular processes underlying adipogenesis, or the transition of dividing preadipocytes into nondividing, lipid-accumulating fat cells, is of pivotal importance and therapeutic interest. Substantial evidence has demonstrated that miRNAs play a crucial role in mouse early embryonic development and may direct germ layer specification [71]. In the current study, we found that miR-10b-5p is a positive regulator in adipocyte lineage commitment and early adipogenesis. Our findings strongly suggest that its depletion hinders the commitment steps of the adipogenesis process, which involves the conversion of ESCs into preadipocytes. Transcript analysis with qRT-PCR and RNA sequencing conducted at specific time points during adipogenesis demonstrated elevated expression of pluripotency markers (Nanog, Oct4 and Sox2) and endoderm markers (Sox7, Gata4) in miR-10b KO cells. In agreement, GSEA revealed a significant enrichment of gene sets linked to pluripotent mechanisms, alongside a potential readiness towards committing to the endodermal lineage. Furthermore, genes involved in BMP signaling, such as Smad9 and Bmp5 [60, 61], were also downregulated in the KO group, which could lead to a reduction of BMP signaling, which is crucial for mesodermal commitment [62]. The GATA family of transcription factors are considered critical for embryonic development, playing fundamental complex roles in cell fate decisions and tissue morphogenesis. Among the GATA factors, Gata4 and Gata6 are recognized for their significance in cell types derived from the endoderm [72]. Gata6 is essential for primitive endoderm specification, as embryos lacking Gata6 default all inner cell mass cells to the epiblast fate and fail to survive implantation. Furthermore, Gata6 heterozygous embryos have delayed primitive endoderm development due to reduced Gata6 levels [73]. Previous research also demonstrated that Nanog and Gata6 co-bind to the same regulatory elements, and their relative levels play a critical role in determining cell fate during early embryonic development [74]. In the present study, we found that Gata6 is a direct target of miR-10b-5p through binding-site prediction and dual-luciferase assay validation. Gata6 was down-regulated by treating KO cells with miR-10b-5p mimic and had an inverse correlation with miR-10b-5p expression during adipogenesis. In miR-10b-5p-KO cells, Gata6 knockdown at differentiation onset increased Myf5, Tbx6, and Dlk1 expression, consistent with enhanced paraxial mesoderm specification, while day-11 cultures showed reduced expression of endoderm-associated markers (Foxa1, Sox17, and Klf4). Previous research has shown that ectopic overexpression of Gata6 in mESCs induced the development of visceral endoderm, while also upregulating the expression of Bmp2. Conversely, deletion of Gata6 prevents embryonic stem cells from differentiating into endoderm [63]. This highlights the critical regulatory role of Gata6 in directing cell fate decisions during early embryonic development [75]. Its expression during brown fat development has not been documented thus far [76]. Nevertheless, in silico analysis identified Gata6 as a potential transcription factor that may modulate the expression of brown fat-specific genes such as Zic1 and enzymes involved in the tricarboxylic acid (TCA) cycle, including citrate synthase (Cs), as documented in the ProFat database [77]. Protein-level analysis of lineage markers (Foxa1, Sox17, Myf5, Tbx6, etc.) was not performed and represents a limitation of this study. However, mRNA quantification is an appropriate readout at early differentiation stages, as these genes encode transcription factors that initiate lineage-defining transcriptional programs and often change prior stable changes in protein levels can be detected at the cellular level. The coordinated upregulation of mesodermal transcripts alongside downregulation of endodermal markers therefore provides a robust molecular signature of lineage redirection. In this study, miR-10b-5p potentially directs cells towards the paraxial mesoderm lineage and facilitates their commitment to preadipocytes by controlling the expression of Gata6 and its downstream target Bmp2. Considered together, these findings offer the first line of evidence that miR-10b-5p might modulate stem cell fate decision, thereby establishing a foundation for deeper investigation into the molecular mechanisms underlying miRNA influence on lineage commitment (Fig. 8). Future studies are warranted to fully define the role of miR-10b-5p as a stem cell fate switch and provide fundamental insights into early mammalian cell fate specification.

In the present study, miR-10b-5p was also identified as a positive regulator in the late stages of adipogenesis, evidenced by a reduction of lipid droplet accumulation and decreased adipogenic gene expression in the presence of miR-10b-5p inhibitor. This halt in adipocyte differentiation into mature adipocytes was accompanied by alterations in the mitochondrial metabolic profile and the presence of reduced metabolically active cells. RNA sequencing identified a significant upregulation of genes linked to G protein signaling associated with increased Tub expression. In line with our transcriptomic results, Tub mRNA and protein levels increased with miR-10b-5p inhibition in BA and declined with miR-10b-5p overexpression in WA. Reporter gene assay indicated that miR-10b-5p directly regulated Tub expression. Moreover, silencing Tub partially reversed the inhibitory effect of miR-10b-5p in the differentiation of brown adipocytes. Mutations in the Tub gene are known to lead to late-onset obesity and insulin resistance in mice [78–81] as well as syndromic obesity in humans [82]. Gene knockout studies of Tub in mice or *C. elegans* produce a similar phenotype as the Tubby mouse seen at the Jackson Laboratories, indicating that the phenotype is due to a loss-of-function mutation [80, 83]. Furthermore, adipogenesis plays a critical role in regulating healthy adipose tissue distribution and remodelling in obesity [84]. If adipogenesis is impaired during excessive energy intake, then existing adipocytes continue to undergo hypertrophy to store excessive energy, leading to metabolic dysfunction [85]. Taking into account our observations on miR-10b-5p and Tub expression during adipocyte differentiation and previously reported association between the Tub and increased risk of obesity [86, 87], we hypothesise that the required transient increase in Tub expression during the early stages of adipogenesis must be regulated by miR-10b-5p expression for adipogenesis to proceed appropriately (Fig. 8).

Despite Tub’s clear physiological relevance to obesity, its precise molecular function remains unclear. Protein structure analysis of Tub revealed a potential DNA-binding groove in its COOH-terminal DNA-binding domain, along with possible transcriptional activation domains in its N-terminal region, strongly indicating that Tub functions as a transcription factor [87]. Santagata et al. [67] demonstrated that Tub protein likely acts as a membrane-bound transcription regulator that translocates to the nucleus in response to phosphoinositide hydrolysis, establishing a direct link between heterotrimeric GTP-binding protein (G protein)-coupled receptors and gene expression regulation. Specifically, Tub localizes to the plasma membrane by binding phosphatidylinositol 4,5-bisphosphate through its carboxyl terminal. Receptor-mediated activation of G protein α_q_ (Gα_q_) induces PLC-β activity, causing Tub to detach from the plasma membrane and translocate to the nucleus [67]. Notably, transfecting Neuro-2 A or HEK293 cells with GFP-Tubby fusion proteins revealed that, after initially localizing to the plasma membrane, fluorescence began accumulating in the nucleus after 36 h. This nuclear accumulation was primarily observed in cells with high expression levels. These findings suggest that Tub may translocate to the nucleus when its membrane binding sites become saturated [67].

Further studies in neuronal cells reinforce this hypothesis, showing that activation of the 5HT-2 C serotonin receptor induces Tub nuclear translocation [67]. In line with these findings, our immunostaining results demonstrated that Tub localisation differs throughout adipocyte differentiation as well as between different adipocyte depots. In brown preadipocytes, Tub is predominantly localized in the nucleus. Upon adipogenic induction, its distribution becomes more uniform throughout the cell. White preadipocytes also show nuclear localisation, although this diminishes at later stages of differentiation. Notably, Tub remained detectable in the cytoplasm throughout WA differentiation. On Day 3, a punctate distribution was still present, albeit to a lesser extent than in BAs. Two transcript variants encoding distinct Tub isoforms have been identified, both sharing the C-terminal domain but differing in their alternatively spliced NH2-terminal regions [67, 80]. The COOH-terminal domain of Tubby directs its localization to the plasma membrane, while the NH2-terminal domain directs it exclusively to the nucleus. The functional nuclear localization signal (NLS) is mapped to the sequence K_39_KKR within the NH2-terminal domain [67, 88]. Therefore, the observed variation in Tub localization might also result from differential splicing. It remains to be determined whether the different isoforms contribute to cell-specific expression of the Tub protein, which can be tested using epitope-specific antibodies. Interestingly, BAs exhibited a distinct fine punctate pattern intracellularly, while WAs showed this to a lesser extent. These structures may represent endosomes, supporting the emerging concept of alternative GPCR signaling via G proteins in intracellular compartments, such as early endosomes, rather than being confined to the plasma membrane [89]. However, further research through endosome staining and co-localization with Tub is needed to confirm this. Furthermore, it would be of value to explore which GPCRs Tub interacts with and whether these interactions vary depending on different stimuli or stages of adipocyte differentiation. Remarkably, previous research has also implicated Tub as being integral to the trafficking of proteins to the primary cilium, a 1- to 10-µm-long antenna-like projection that extends from the plasma membrane. Primary cilia sense extracellular cues via receptors and relay signals from pathways like GPCRs, RTKs, Hedgehog, and Wnt, regulating an array of cellular processes including cell proliferation, migration, and differentiation [90–92]. Recent studies have shown that primary cilium dysfunction influences adipogenesis [93, 94]. During adipocyte differentiation, the cilium appears in confluent cells, elongates early on, and disappears as lipid accumulation increases [95], mirroring the Tubby expression pattern we observed in brown adipocytes. Given that GPCR trafficking in and out of the cilium is tightly regulated by the Tubby protein family, these findings highlight a potential role for Tub in coordinating ciliary signaling during adipocyte differentiation.

Previous research in rodent adipocytes and neuronal cells has shown that Tub expression is regulated by insulin and T3, two key hormones involved in the regulation of metabolism [96, 97]. Insulin treatment of CHO-IR and PC12 cells induces tyrosine phosphorylation of Tub and facilitates its interaction with SH2 domain-containing proteins [98]. Furthermore, cell sensitivity to insulin appears to regulate Tub expression in adipocytes [96]. Stretton et al. [96] demonstrated that 3T3-L1 adipocytes exposed to chronic high insulin develop insulin resistance and show increased Tub levels, which are reduced by the insulin-sensitizing drug rosiglitazone. Consistently, we observed higher Tub expression in early differentiation, followed by a decline throughout the process, even when the miR-10b-5p inhibitor was present. The observed reduction in Tub during adipogenesis is likely, at least in part, due to insulin-induced repression of the Tub gene. Insulin suppresses the expression of several genes encoding proteins with key metabolic functions [99] and Tub may be part of the group of repressed genes.

Notably, miR-10b KO ESCs, as well as BAs treated with miR-10b-5p inhibitor, exhibited increased Ucp1 on day 0, resulting in elevated cellular respiration. This was also evident from the increased expression of transcripts associated with Mitochondrial Fatty Acid Beta Oxidation, Fatty acid Metabolism and PPAR signaling on day 0 in BAs [100] A transient increase in PPARG and UCP1 expression in human subcutaneous SVF cells was previously observed during differentiation. Similarly, another study found that UCP1 and PPARG peaked on day 14 and declined by day 28 post-differentiation in SGBS adipocytes [101], suggesting that cells may transiently adopt a brown adipocyte-like phenotype while differentiating to white adipocytes. Our results show that miR-10b depletion in cells may temporarily activate this process that would otherwise suppress Ucp1 and Pparg, as observed in brown preadipocytes and fully differentiated BAs treated with the inhibitor.

We also examined whether increased miR-10b-5p could induce a browning phenotype in white adipocytes. Mimic treatment enhanced adipogenesis and thermogenic effects as seen by increased Ucp1 and Ppargc1a, alongside bioenergetic changes. Collectively, our findings suggest a role for miR-10b-5p in metabolic processes critical for lipid and energy metabolism.

Pathogenesis of obesity has been linked to reduced levels of miR-10b-5p in mouse and rat [102]. Furthermore, Herrera et al. [103] found decreased miR-10b-5p expression in response to hyperglycemia, suggesting its role in type 2 diabetes pathophysiology in the Goto–Kakizaki rat model. This pattern was consistent with the miR-10b-5p expression profile observed in children with type 1 diabetes (T1D) [104], twins with type 2 diabetes (T2D) [105] and T2D patients [106, 107], strengthening the critical role of miR-10b-5p during metabolic dysfunction. Singh et al. [108] further showed that miR-10b loss in β-cells and interstitial cells of Cajal (ICC) triggered diabetes and GI dysmotility in male mice via the miR-10b-KLF11-KIT pathway, which regulates glucose and gut motility. This effect was absent in female mice, highlighting hormonal differences in T2D susceptibility. They later found that in female T2D mice, characterized by insulin resistance, the pathogenesis was driven by the miR-10a/b-5p-NCOR2-INSR axis, and miR-10a/b-5p treatment improved glycaemic control. The in vivo relevance of miR-10b-5p was further corroborated by a global mouse knockout model. Specifically, congenital loss of miR-10b-5p in mice resulted in body weight gain, increased susceptibility to diet-induced obesity, hyperglycaemia, impaired glucose tolerance and insulin resistance [106]. Interestingly, miR-10b-5p mimic treatment reversed GI dysmotility, restored glucose tolerance and insulin sensitivity in transgenic mice where miR-10b-5p was knocked out, highlighting its superior long-term efficacy in treating both diabetes and GI dysmotility compared to commonly used antidiabetic and prokinetic medications [106, 108]. In addition to its role in controlling metabolism, its relevance in the regulation of cell differentiation was previously described in vitro. In agreement with our study, the expression of miR-10b-5p was previously found to be significantly upregulated during adipogenesis in 3T3-L1 cells. At the molecular level, the activity of apolipoprotein L6 (Apol6), a lipid-binding protein that plays a key role in adipogenesis, was shown to be regulated by miR-10b-5p [102]. On the contrary, conflicting results by Li et al. 2018 [109] showed that miR-10b increased during osteogenic differentiation but declined during adipogenic differentiation in human adipose-derived mesenchymal stem cells (hADSCs). Furthermore, overexpression of miR-10b was found to enhance osteogenesis and inhibit adipogenesis, with downregulation reversing these effects. ADSCs are commonly used to study adipogenesis, but their use is limited by the inherent heterogeneity of the population, which can vary based on the source and isolation method [110]. This heterogeneity can lead to inconsistent results in adipogenesis studies, as it fails to account for differences between white and brown adipose tissue depots, potentially masking depot-specific variations in cell phenotype. Moreover, variations in culture techniques, media formulations, and adipogenic differentiation protocols can impact the consistency and reproducibility of results in adipogenesis studies. Further conflicting findings by Tan and colleagues [102] demonstrated that the downregulation of miR-10b-5p promoted the differentiation of 3T3-L1 cells and adipogenesis by upregulating the Apol6 expression. Their study achieved miR-10b-5p KD or upregulation by treating cells every two days, raising the possibility that repeated exposure to transfection reagents influenced the differentiation process. This potential impact is reflected in their Oil Red O staining, where differentiation appears relatively low even on day 8. Importantly, Fabp4, a robust marker of adipogenesis [111], seems to be upregulated in response to both miR-10b-5p mimic and inhibitor when compared to control. In contrast, our approach minimizes these variables, providing a clearer assessment of miR-10b-5p role during adipogenesis.

There are still several limitations in this study. For instance, we only investigated the effect of miR-10b-5p in in vitro experiments and did not further verify the proposed mechanism in vivo. Secondly, the mature miR-10a-5p and miR-10b-5p are part of the miR-10 family, differing by just a single nucleotide in the middle of their sequences [112]. These two miRNAs play a crucial role in diabetes regulation [108]. miR-10a-5p was highly expressed in brown preadipocytes but downregulated upon differentiation, reaching levels similar to white mature adipocytes. Its expression in white adipocytes remained unchanged between preadipocytes and mature cells. This remains a limitation of the study, as it has not been specifically investigated. Nevertheless, Zogg et al. [106] showed that global knockout of miR-10b in a mouse model still resulted in metabolic syndrome symptoms, with only a slight increase in miR-10a-5p, suggesting that miR-10b-5p alone plays a critical role in the development of metabolic dysfunction.

## Conclusion

This study shows that miR-10b-5p inhibition plays a dynamic role in adipocyte biology, as its inhibitory effects manifest differently during the stem cell commitment state and the maturation phase of adipocytes. miR-10b-5p regulates early adipogenesis by influencing cell fate bias toward the paraxial mesoderm lineage through modulation of the Gata6/Bmp2 signaling axis, and later stages by downregulating Tub. Understanding the miR-10b-5p-mediated regulatory mechanism during adipocyte commitment and differentiation may help to generate adipose tissue-engineering strategies for cellular therapies for lipodystrophy and obesity.

## Supplementary Information

Supplementary Material 1.

Supplementary Material 2.

## Data Availability

The datasets used and/or analysed during the current study are available from the corresponding author on reasonable request. RNA sequencing datasets, including those from miR-10b +/+ and miR-10b −/− mESCs during adipocyte differentiation on days 0, 12, and 27 (E-MTAB-15151); RNA sequencing analysis of miR-10b-5p KD during brown adipogenesis(E-MTAB-15152); and the identification of differentially expressed miRNAs between mouse scWAT and iBAT (E-MTAB-15230) are available at ArrayExpress.

## Abbreviations

ADSCs: Adipose derived mesenchymal stem cells
AP: Alkaline Phosphatase
BA: brown adipocyte
BMPR: BMP receptor
DEGs: differentially expressed genes
ECAR: extracellular acidification rate
ES: embryonic stem
FACS: fluorescence-activated cell sorting
GO: Gene ontology
GPCRs: G protein-coupled receptors
GSEA: Gene set enrichment analysis
HRMA: high-resolution melt analysis
iBAT: interscapular brown adipose tissue
IPSC: Induced pluripotent stem cell
KO: knockout
LIF: Lukemia Inhibitory Factor
LNA: locked nucleic acid
mESCs: mouse embryonic stem cells
miRNA: microRNA
MSC: mesenchymal stem cell
NC: negative control
NLS: nuclear localization signal
NTC: non-targeted control
OCR: oxygen consumption rate
PCA: principal component analysis
scWAT: subcutaneous white adipose tissue
sgRNA: single guide RNA
SVF: stromal vascular fraction
TGs: triglycerides
TPM: transcripts per million
T1D: Type 1 Diabetes
T2D: Type 2 Diabetes
UTR: untranslated region
WA: white adipocyte
β3AR: beta 3 adrenergic receptor
