# Supplementary material for: miR-10b-5p regulates adipocyte lineage commitment and adipogenesis via targeting of Gata6 and Tubby
Source: Cell Commun Signal. 2026 Mar 28;24:276. doi: 10.1186/s12964-026-02834-y (PMC13151333; doi:10.1186/s12964-026-02834-y)
Supplement: Supplementary file 1 — Supplementary Material 1. [file 12964_2026_2834_MOESM1_ESM.pdf]

Supplementary materials

Supplementary Tables

Supplementary Table 1. ***Brown Fat Differentiation Protocol***

Induction media was administered for 2 days. Following induction, cells were treated with maintenance medium for 6 to 8 days with medium being replenished every 2 days. Components were added in complete medium that included DMEM/F12, 10%FBS, 1% L-Glutamine and 1% Penicillin-Streptomycin antibiotics.

Compound	Induction Medium	Maintenance Medium
Insulin	1 µg/ml	1 µg/ml
3,3′ ,5-Triiodo-L-thyronine sodium salt (T3)	1 nM	1 nM
IBMX	0.5 mM	
Dexamethasone	250 nM	
Indomethacin	125 µM	

Supplementary Table 2. ***White Fat Differentiation Protocol***

Induction medium 1 was administered for 3 to4 days. Following induction medium 1, cells were treated with induction medium 2 for 2 to 3 days and then with maintenance medium for 2 to 3 days. Components were added in complete medium that included DMEM/F12, 10%FBS, 1% L-Glutamine and 1% Penicillin-Streptomycin antibiotics.

Compound	Induction Medium 1	Induction Medium 2	Maintenance Medium
Insulin	5 mg/ml	5 mg/ml	5 mg/ml
IBMX	0.5 mM		
Dexamethasone	250 nM		
3,3′,5-Triiodo-L-thyronine sodium salt (T3)	0.1 nM	0.1 nM	0.1 nM
Cortisol	100 nM		
Transferrin	1 mg/ml	1 mg/ml	1 mg/ml
Rosiglitazone	5 µM	1 µM	

Biotin	16 μM	16 μM	16 μM
Panthothenic acid	1.8 μM	1.8 μM	1.8 μM
Ascorbic acid	100 μM	100 μM	

Supplementary Table 3. ***Human brown adipocyte differentiation protocol***

Compound	Induction Medium	Maintenance medium
Insulin	1 μg/ml	1 μg/ml
3,3′,5-Triiodo-L-thyronine sodium salt (T3)	10 nM	0.1 nM
IBMX	500 μM	
Dexamethasone	250 nM	
Indomethacin	125 μM	
Rosiglitazone	5 μM	

Supplementary Table 4. ***Primer sequences for human genes.***

Gene name	Forward primer (5' to 3')	Reverse primer (5' to 3')
CIDEA	CATGTATGAGATGTACTCCGTGTC	GAGTAGGACAGGAACCGCAG
CIDEC	TAAGCACGGCGGATCGAA	CAGAGTGTCCCGGACCTTGA
GATA6	CACACCACAACTACCACCTTAT	TCCTGGTTTGAATTCCCTCTTT
FABP4	AACTGGTGGTGGAATGCGT	AACTGGTGGTGGAATGCGT
LN19	GCGGAAGGGTACAGCCAA	GCAGCCGGCGCAAAA
DLK1	CACGGACTCTGTGGAGAACC	GCAGGCCCGAACATCTCTAT
UCP1	GTGTGCCCAACTGTGCAATG	CCAGGATCCAAGTCGCAAGA

Supplementary Table 5. ***Primer sequences for mouse genes.***

Gene name	Forward primer (5' to 3')	Reverse primer (5' to 3')
BMP2	GAACACAAGTCAGTGGGAGAG	CACCTGGGTTCTCCTCTAAATG
CIDEA	CACGCATTTCATGATCTTGGA	GTTGCTTGCAGACTGGGACAT
DAB2	GACGCTTTCACTGGCTTAGA	CCTTCCTTGAGGGAACAAGAG
DLK1	CGGGAAATTCTGCGAAATAG	TGTGCAGGAGCATTCGTACT
FABP4	ACACCAGATTTCCTTAAACTG	CCATCTAGGGTTATGATGCTCTTCA
FOXA1	TGGCTCCAGGATGTTAGGGA	GTGTCCGCGTAGTAGCTGTT
GATA4	GGAAGCCCAAGAACCTGAATA	CTAGTGGCATTGCTGGAGTTA
GATA6	GCCTTGTCTGCTAAGGAAGAT	GGATGAATGGGTTCTGGGATAA
HOXD3	GGCAGCGCCGGATGGAT	CTGCTGAATCTTGAGAGAGCTGG
KLF4	GTGCCCCGACTAACCGTTG	GTCGTTGAACTCCTCGGTCT
LN19	GAGCACATCCACAAGCTGAA	TTTCGTGCTTCCTTGGTCTT
MYF5	TCTGACGGCATGCCTGAAT	TGCATTTGATACATCAGGACAGT
NANOG	GCCTCCAGCAGATGCAAG	GGTTTTGAAACCAGGTCTTAACC
OCT4	CGTGGAGACTTTGCAGCCTG	GCTTGGCAAACTGTTCTAGCTCCT
SOX7	TCACCTCCCCCATCTACCAG	GGCCAAGGGCTAAAGAACCT
SOX17	GATGCGGGATACGCCAGTG	CCACCACCTCGCCTTTCAC

TBX6	ATGTACCATCCACGAGAGTTGT	GGTAGCGGTAACCCTCTGTC
TUB	ACAATGGCGTCAACCCTCAG	CTGGGACGATCACACTCATCTTC
UCP1	TACCCAAGCGTACCAAGCTG	ACCCGAGTCGCAGAAAAGAA

Supplementary Table 6. ***Primer sequences for Luciferase and mutagenesis assays.***

Gene name	Forward primer (5' to 3')	Reverse primer (5' to 3')
Gata6 3'UTR	GCTGGTGCTACCAAGAGGC	GGTTGGTCACGTGGTACAGG
Tub 3'UTR	GTTTCTAGAGGGCAGTAGGAC	GCCTGTCCTCACCAAGCTG
Gata6 3'UTR mutagenesis	AGAAAAATATCTTGTTTGCTACCAGATTTACAAATTCCAAGTGACCTCAGATCAGCC	GGCTGATCTGAGGTCACTTGGAATTTGTAAATCTGGTAGCAAACAAGATATTTTTCT
Tub 3'UTR mutagenesis	TAGAGATGACTGCTTAGCTAGGAAGCTCTGCTCTG	CAGAGCAGAGCTTCCTAGCTAAGCAGTCATCTCTA

Supplementary Table 7. ***CRISPR/Cas9 sgRNA sequences without the NGG end and primers used for HRMA.***

Sequence name	Forward sequence (5' to 3')	Reverse sequence (5' to 3')
miR-10b-5p sgRNA	CCTGTAGAACCGAATTTGTG	CACAAATTCGGTTCTACAGG
Non-Targeted Control sgRNA	GGGTCTTCGAGAAGACCT	AGGTCTTCTCGAAGACCC
HRMA primers	CCGAGGTTGTAACGTTGTC	CCATGTCGGAGATATATGAAG

Supplementary Table 8. ***Oligonucleotide sequences of miRNA inhibitors and mimics.***

name	Catalogue number	Inhibitor sequence
mmu-miR-10b-5p miRCURY LNA miRNA Inhibitor	YI04100556-DDA	ACAAATTCGGTTCTACAGGGT
miRCURY LNA miRNA Inhibitor Control	YI00199006	TAACACGTCTATACGCCCA
**name**	**Catalogue number**	**Mature miRNA sequence**
hsa-miR-10b-5p miRCURY LNA miRNA Mimic	YM00472145	UACCCUGUAGAACCGAAUUUGUG
Negative Control miRCURY LNA miRNA Mimic	YM00479902	UCACCGGGUGUAAAUCAGCUUG

Supplementary Table 9. **On/off target sites of sgRNA miR-10b generated by CCTop - CRISPR/Cas9 target online predictor.** (web tool accessed at 17:32pm, 07/12/23)

Coordinates	MM	target_seq	PAM	position	gene name	gene id
chr2:74726077-74726099	0	CCTGTAGA[ACCGAATTTGTG]	TGG	Exonic	Mir10b	ENSMUSG00000065500
chr2:105460157-105460179	4	TATGTGGA[AACGAATTTGTG]	AGG	Intergenic	4930527A07Rik	ENSMUSG00000086764
chr15:59583720-59583742	4	ATTGGAGA[ACTGAATTTGTG]	TGG	Intronic	Nsmce2	ENSMUSG00000059586
chr5:109894569-109894591	4	TCTTTAAA[ACGGAATTTGTG]	GGG	Intergenic	Gm26779	ENSMUSG00000097140
chr9:41431921-41431943	4	GCTGAACA[ACTGAATTTGTG]	GGG	Exonic	2610203C20Rik	ENSMUSG00000074415
chr1:58718061-58718083	4	GCTGCAGT[ACTGAATTTGTG]	GGG	Intronic	Cflar	ENSMUSG00000026031
chr8:106690664-106690686	4	TCTGGAGC[ACAGAATTTGTG]	TGG	Intronic	Tango6	ENSMUSG00000041949
chr4:40339598-40339620	4	TCTATAGA[GCTGAATTTGTG]	AGG	Intergenic	4930509K18Rik	ENSMUSG00000087137
chr15:72912507-72912529	4	AGTGTAGA[AGCTAATTTGTG]	AGG	Intronic	Gm3150	ENSMUSG00000096173
chr16:84708197-84708219	3	CTTGTAGA[ACTTAATTTGTG]	TGG	Intronic	Mir155hg	ENSMUSG00000097418
chr18:40483559-40483581	4	ACTGTAGT[CCTGAATTTGTG]	GGG	Intronic	Kctd16	ENSMUSG00000051401
chr5:16220572-16220594	4	CCTTCAGA[AATGAATTTGTG]	TGG	Intronic	Cacna2d1	ENSMUSG00000040118
chr18:29833061-29833083	4	TCTTTAGA[AACCAATTTGTG]	CGG	Intergenic	NA	NA
chr19:42216214-42216236	4	CCTTTAGT[ATCTAATTTGTG]	TGG	Intergenic	Sfrp5	ENSMUSG00000018822
chr17:3969757-3969779	4	CATTTACA[ACCGAAATTGTG]	AGG	Intergenic	NA	NA

chr6:36427248-36427270	4	ACAGTAGA[ACAGATTTTGTG]	TGG	Intronic	Mir490	ENSMUSG00000070075
chr5:124266586-124266608	4	CTTTTAGA[ACCTAGTTTGTG]	TGG	Intronic	Mphosph9	ENSMUSG00000038126
chr11:107860036-107860058	4	TCTGCAGA[AGCGAATGTGTG]	TGG	Intergenic	Gm27595	ENSMUSG00000098991
chr12:112107864-112107886	3	CCTGTTGA[ACCTAATTGGTG]	TGG	Intronic	Aspg	ENSMUSG00000037686
chr15:3327419-3327441	4	CCTGAACA[ACAGAATTTCTG]	GGG	Intronic	Ghr	ENSMUSG00000055737

Supplementary Table 10. ***Study design of the transcriptomic analysis performed on mESCs differentiation to mature adipocytes.***

**Day 0**		**Day 12**		**Day 27**	
**Clone**	**Number of samples**	**Clone**	**Number of samples**	**Clone**	**Number of samples**
NTC Clone 1	3	NTC Clone 1	1	NTC Clone 1	3
NTC Clone 2	1	NTC Clone 2	2	NTC Clone 2	
NTC Clone 3	1	NTC Clone 3	2	NTC Clone 3	1
1F10	3	1F10	3	1F10	2
3G6	1	3G6	1	3G6	
3F2	1	3F2		3F2	1

Supplementary Figures

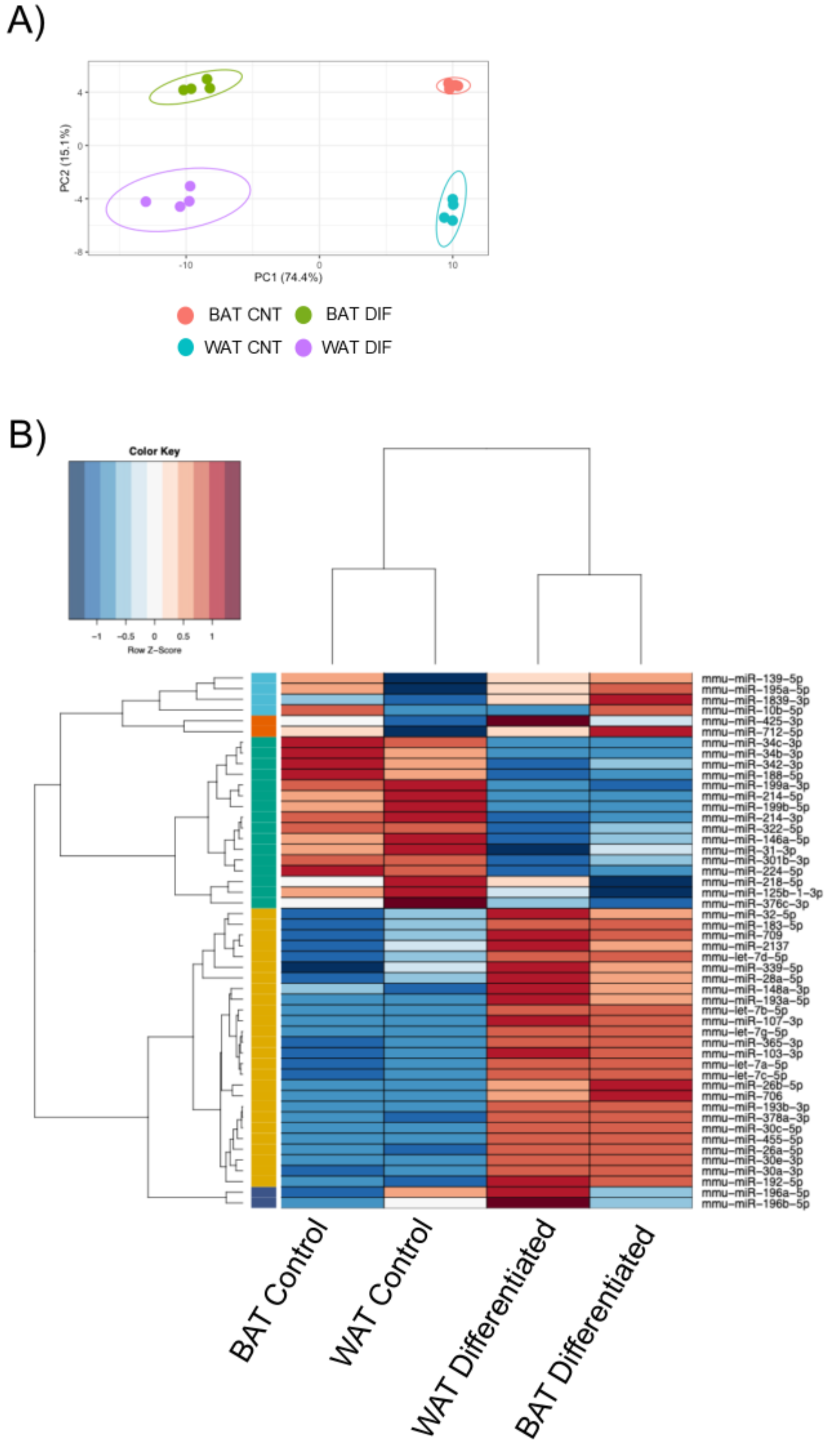

Figure S1. PCA and Hierarchical Clustering highlight distinct secreted miRNA signatures in brown and white adipocyte differentiation.

A) PCA plot reveals distinct secreted miRNA profiles during brown and white adipocyte differentiation. B) Heat Map and Unsupervised Hierarchical Clustering were conducted on the 50 miRNAs with the highest coefficient of variation based on normalized (dCq) values across all samples.

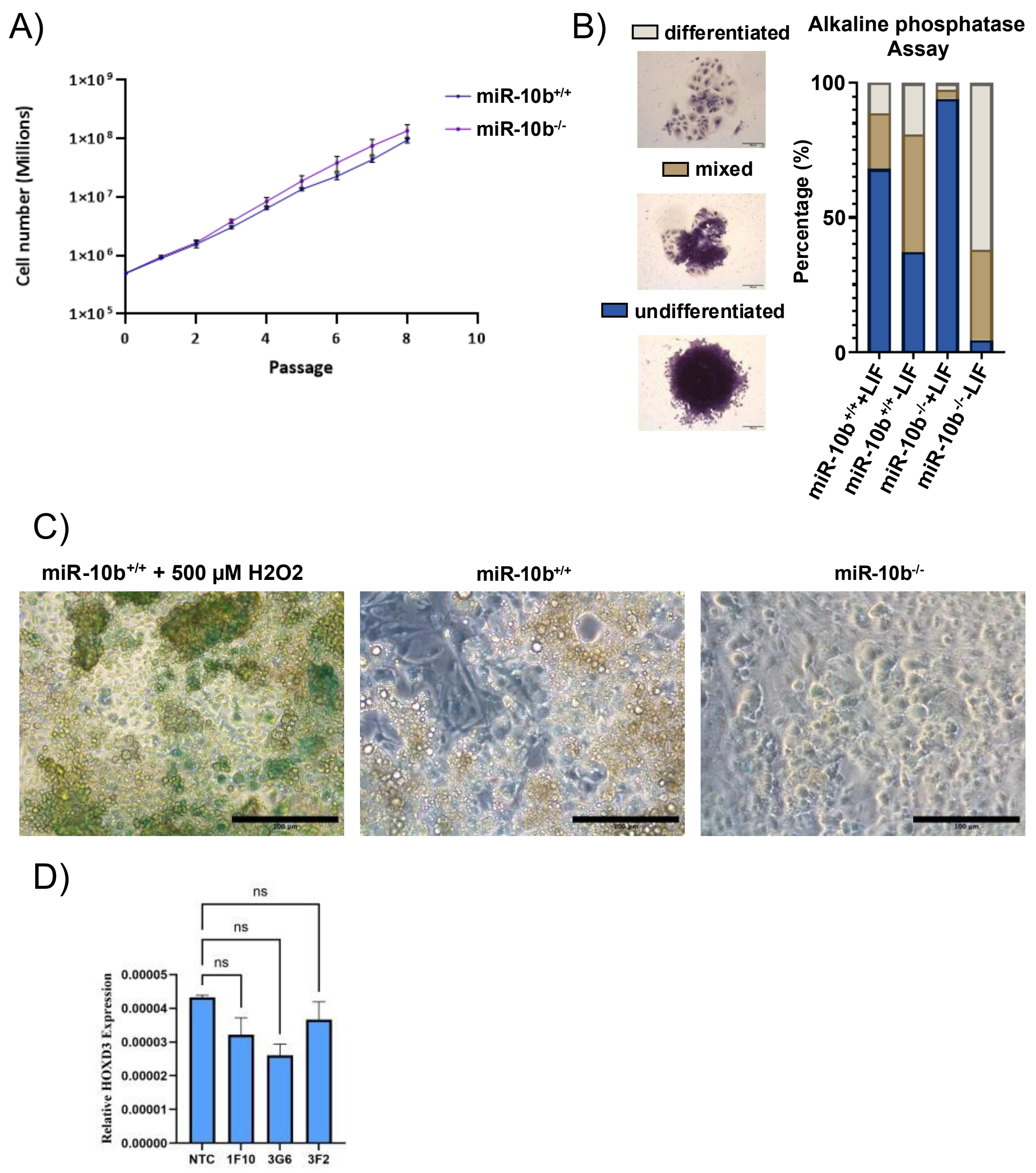

Figure S2. Assessment of Cellular Dynamics in mESCs with or without excised miR-10b: Proliferation, Senescence, and Alkaline Phosphatase Activity.

A) Cell proliferation assay. Data are representative of three independent experiments and values are expressed in mean ± SEM. B) Alkaline Phosphatase (AP) activity was used to assess the self-renewal capacity of mESCs. Scale bars: 100 µm. C) Cell senescence was analysed in cells with or without depleted miR-10b. miR-10b+/+ and

miR-10b-/- stem cells were differentiated to mature adipocytes. At day 27, cells were stained for β-galactosidase and observed at ×20 magnification. For positive control, miR-10b-/- were treated with 500 µM H2O2 for 1 hour. Scale bar represents 100 µM. β-galactosidase-positive cells appear light blue. D) HOXD3 mRNA levels were quantified to assess how they changed following CRISPR-mediated knockout of the miR-10b locus. Data were analysed with ANOVA test and are presented as mean ± SEM (n ≥ 3).

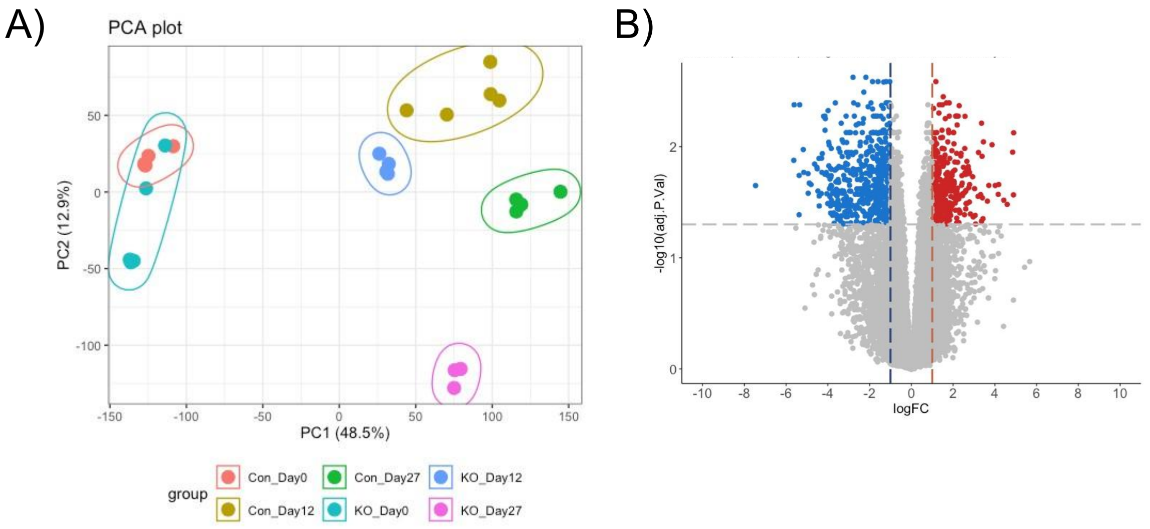

Figure S3. Transcriptomic analysis was conducted on mESCs differentiating to mature adipocytes at day 0, 12, and 27.

A) PCA plot for stem cells time course. B) Volcano plot illustrating the expression profile of genes in mESCs treated with gRNA targeting miR-10b or NTC vector at Day 0 (log_2(FC) > 1 and adjusted p value < 0.05).

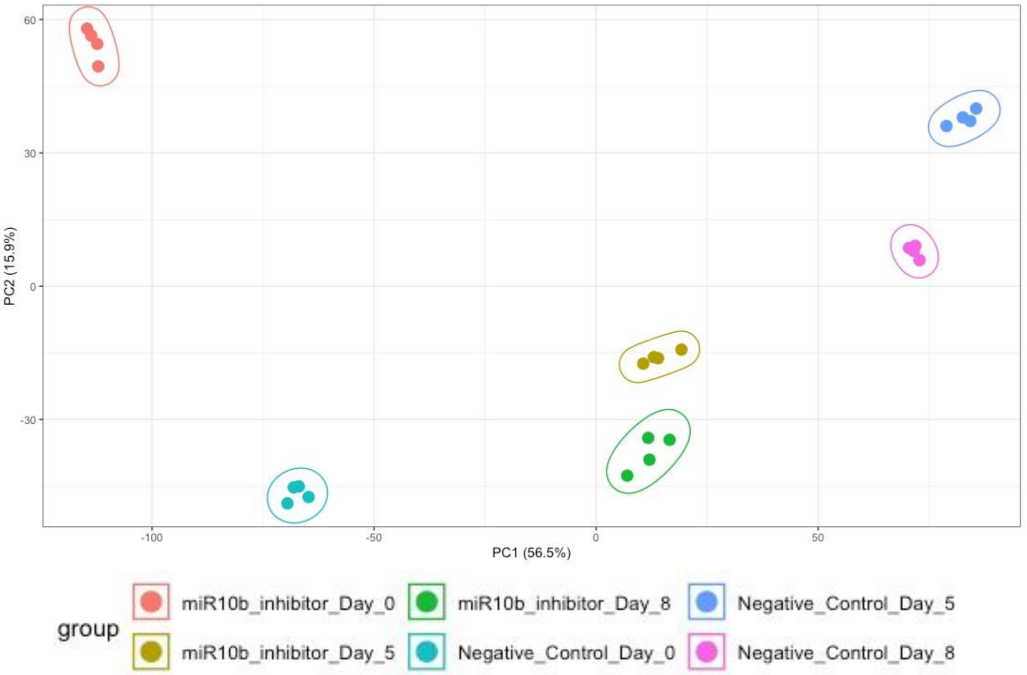

Figure S4. PCA plot for BAT time course.

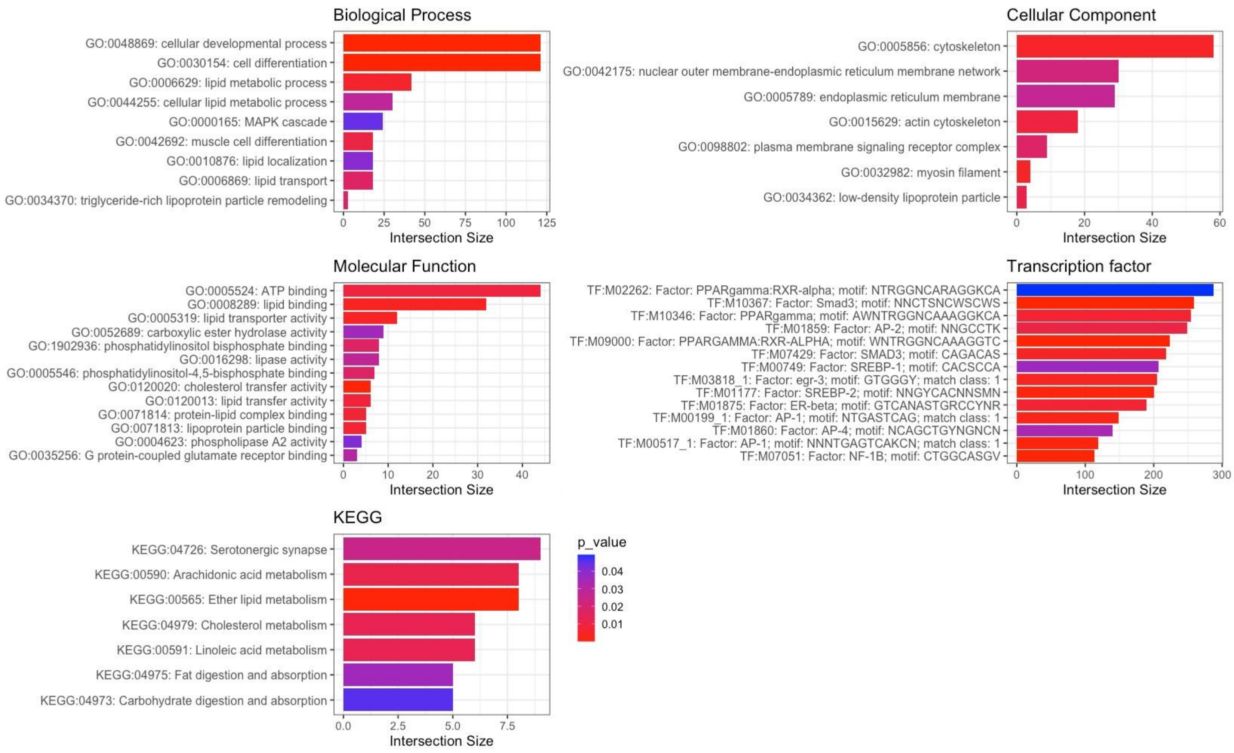

Figure S5. GO enrichment analysis of related DEGs in purple module.

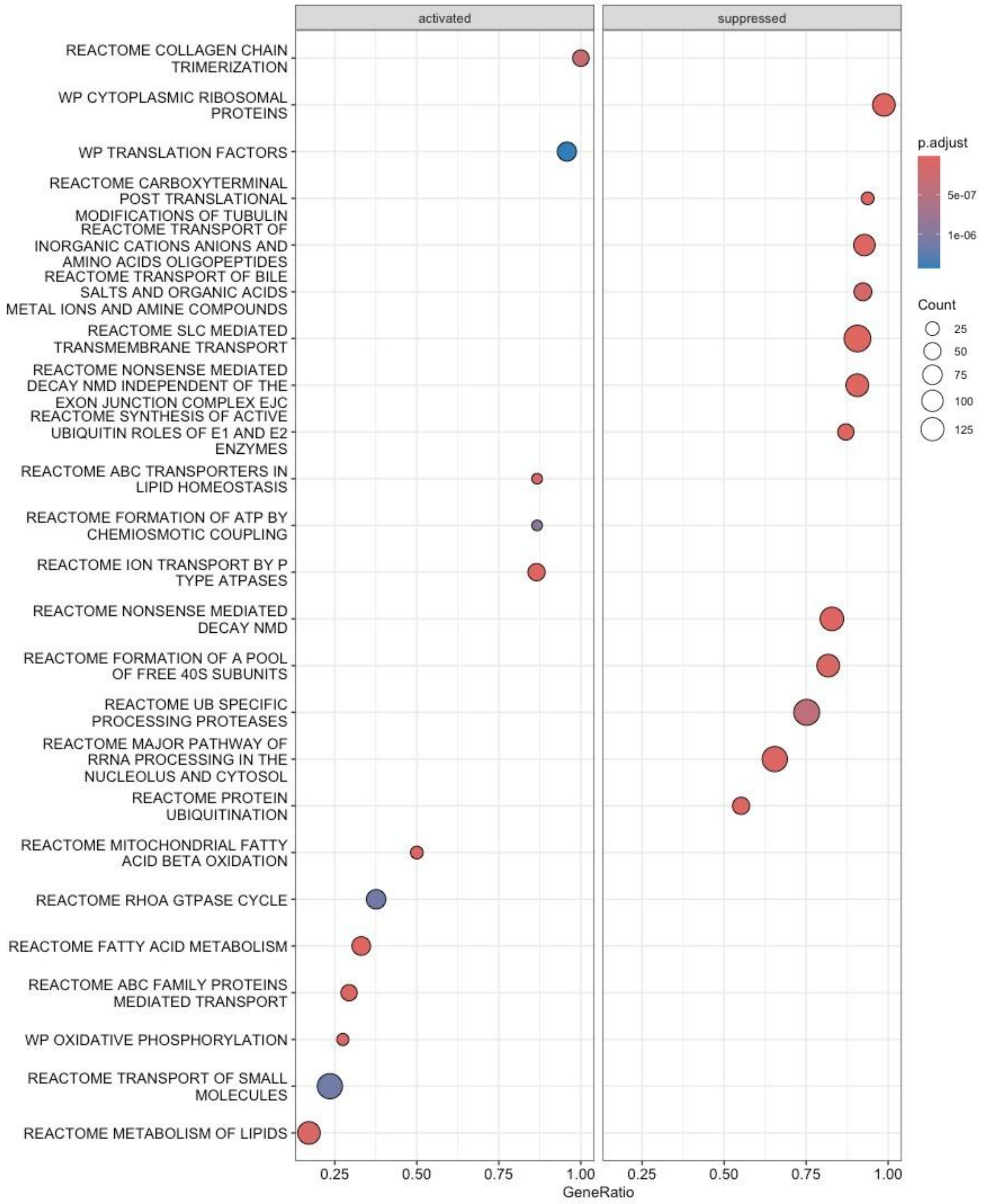

Figure S6. Dot plot illustrating the top 12 enriched biological pathways in the DEGs between brown adipocytes treated with miR-10b inhibitor and negative control at Day

0. The count represents the number of inputted DEGs as a percentage of the total number of genes. The Benjamini p value for each molecular mechanism is shown.

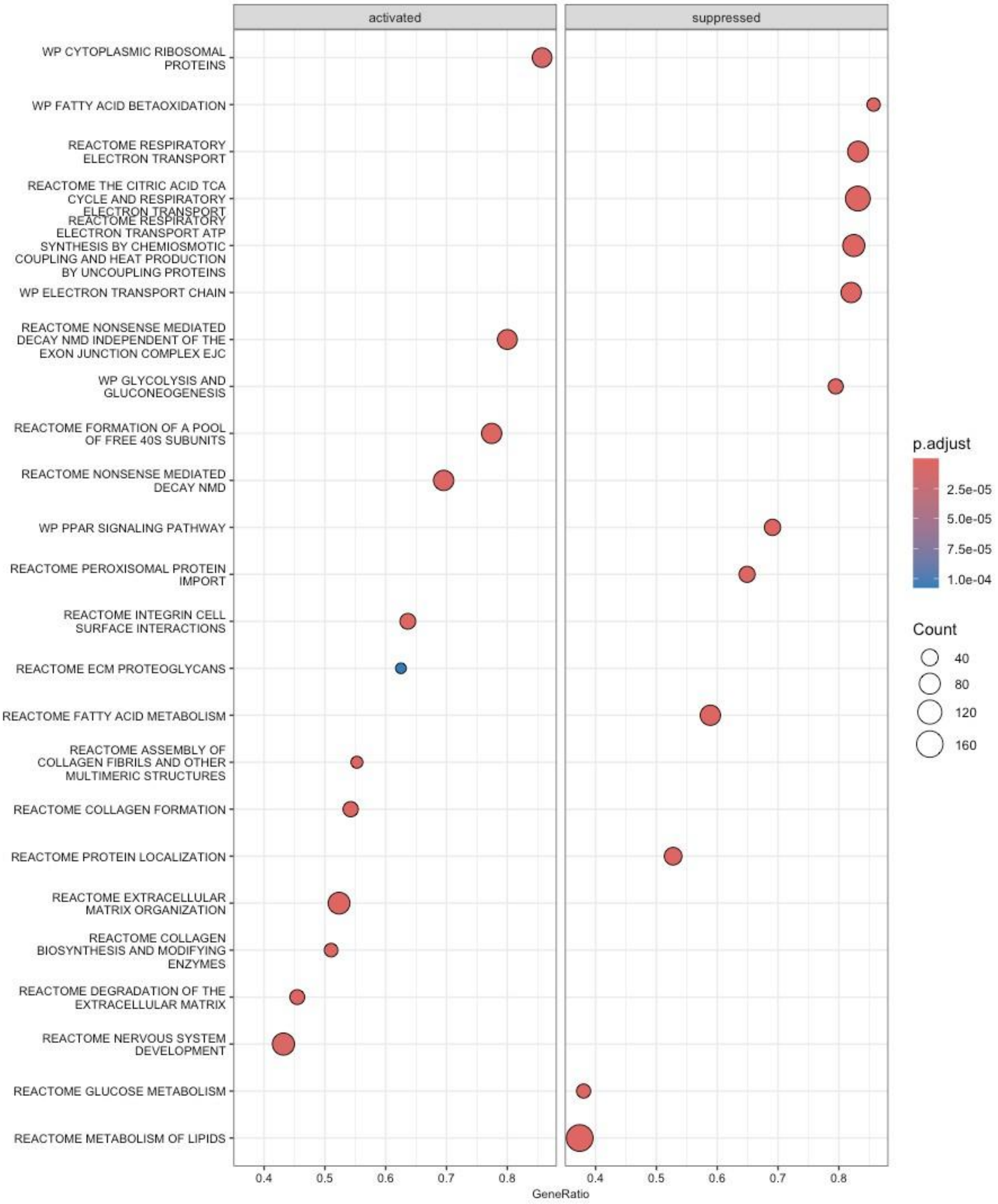

Figure S7. A dot plot displays the top 12 enriched biological pathways among the DEGs in brown adipocytes treated with a miR-10b inhibitor compared to the negative

control at Day 5. Each dot represents the percentage of DEGs within the total number of genes. Additionally, the Benjamini-adjusted p-value for each molecular mechanism is presented.

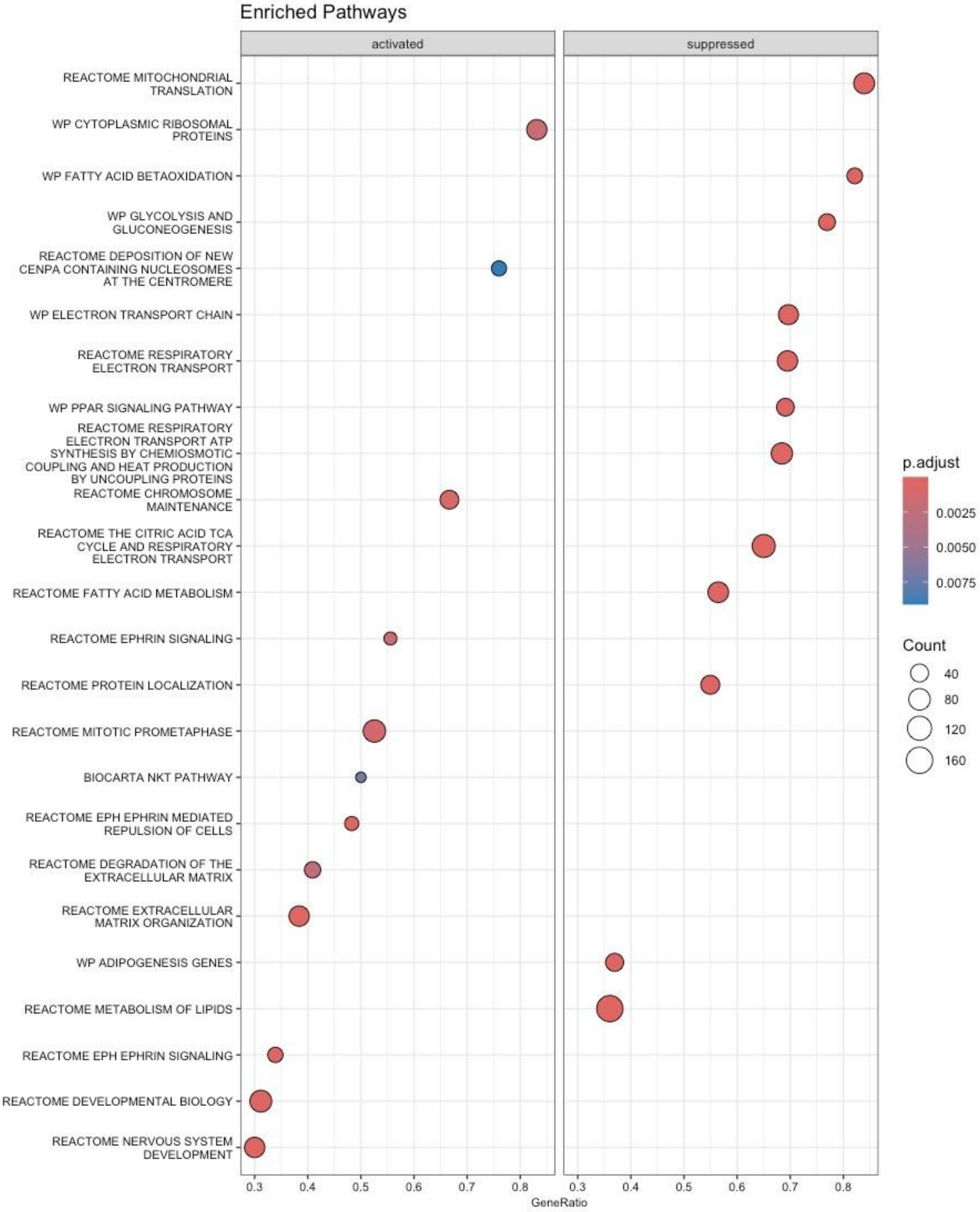

Figure S8. A dot plot is used to depict the top 12 enriched biological pathways among the DEGs in brown adipocytes treated with a miR-10b inhibitor compared to a

negative control at Day 8. The dots represent the percentage of DEGs within each pathway relative to the total number of genes in that category. Additionally, the Benjamini p-value for each pathway is displayed to indicate its statistical significance.

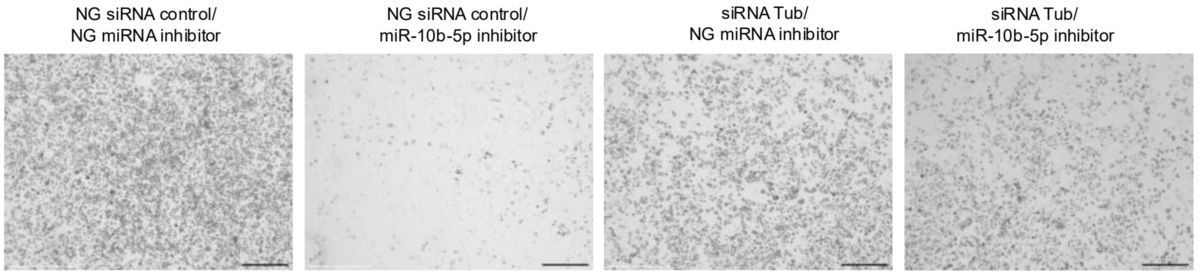

Figure S9. Representative bright-field images depicting the degree of differentiation by visualising the amount of lipid droplet formation on day 6 of differentiation. Magnification 4x. Scale bar 500 µm (black).

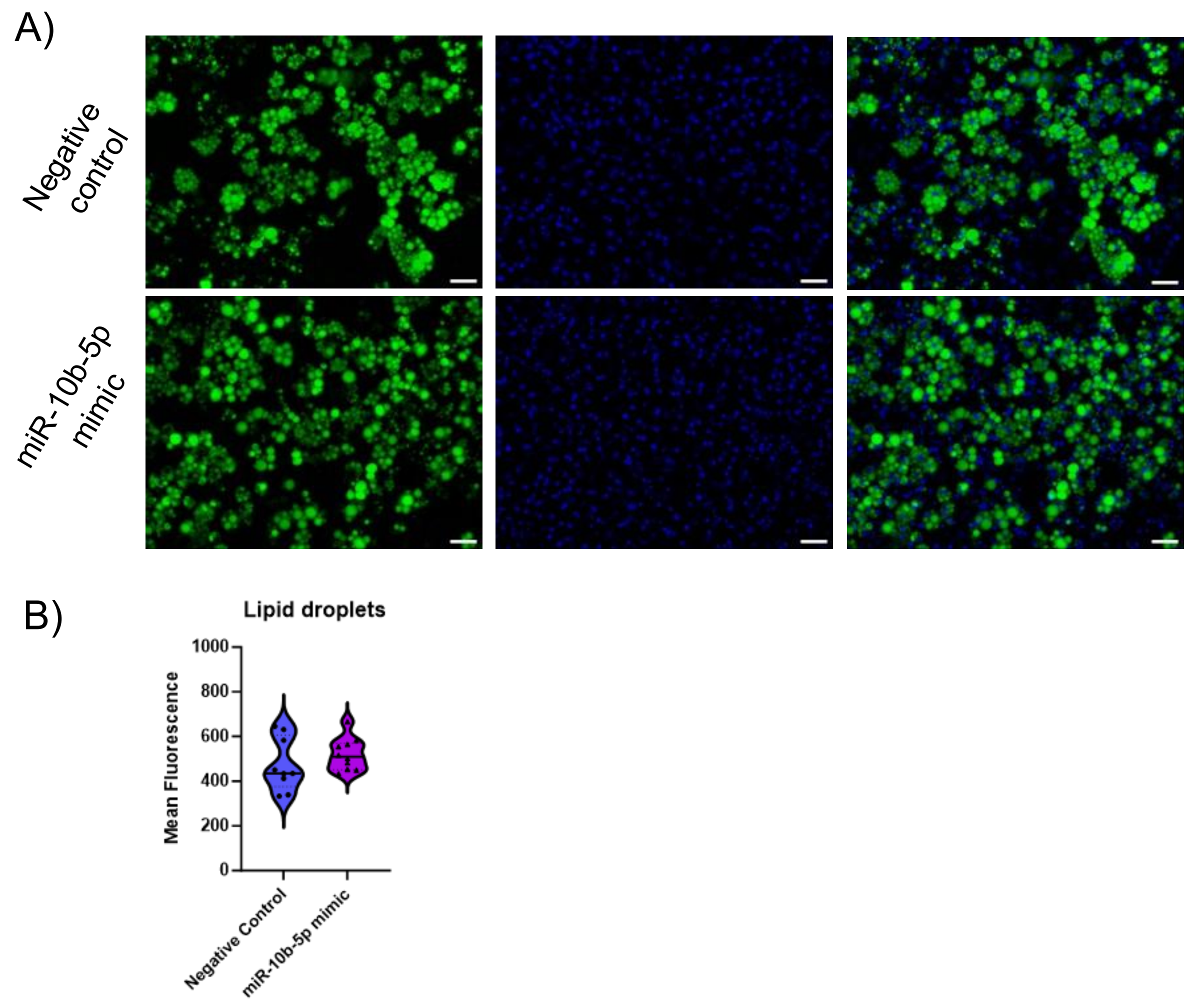

Figure S 10. The effects of elevated miR-10b-5p levels on white adipogenesis.

A) *White preadipocytes were treated with either negative control or miR-10b-5p mimic for 48 hours and differentiated for 8 days, followed by staining of their lipids using a GFP lipid stain and their nucleus with DAPI. Scale bar: 50 B) Lipid droplet quantification.*

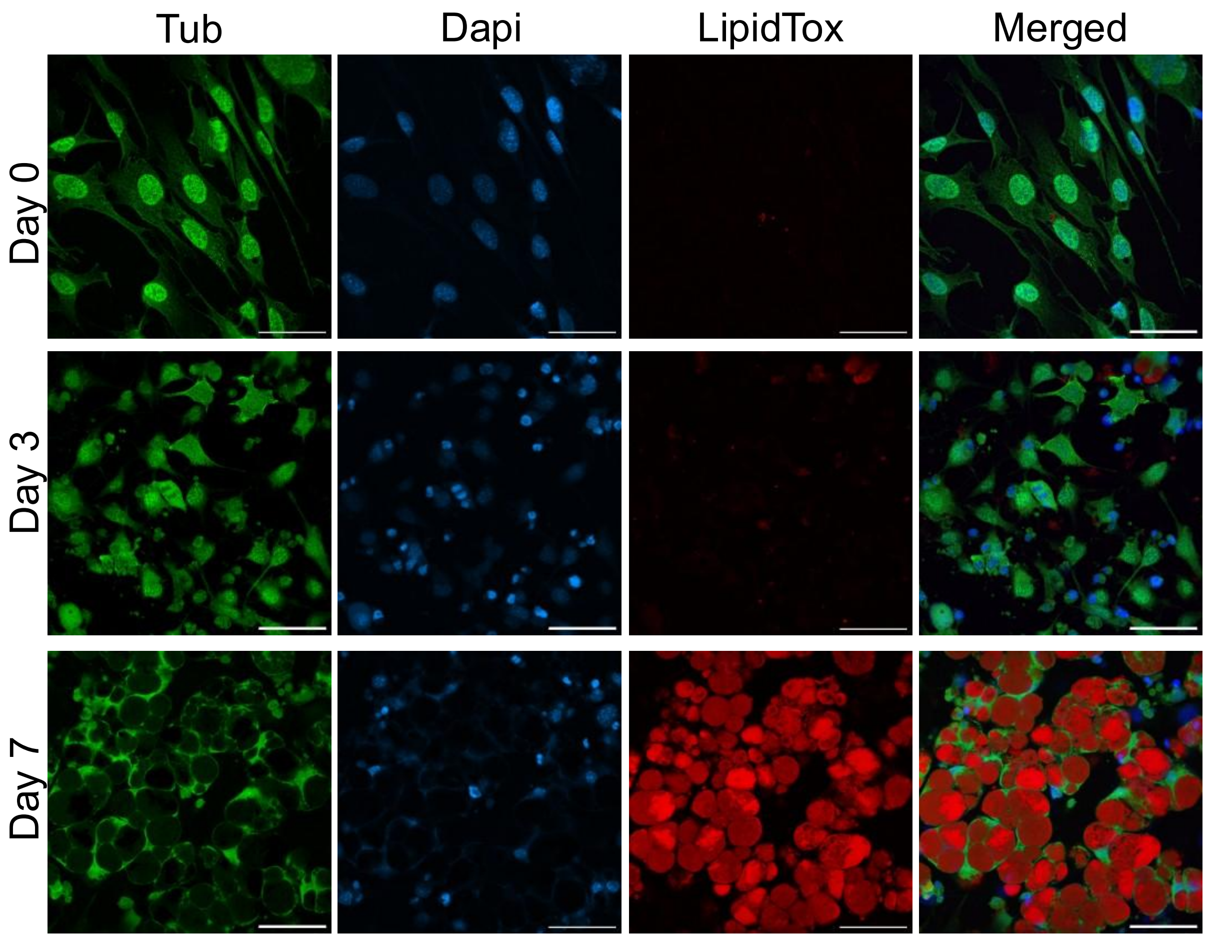

112

113 *Figure S11. Tub immunostaining during WAT differentiation. Scale bar: 50 μm.*

114

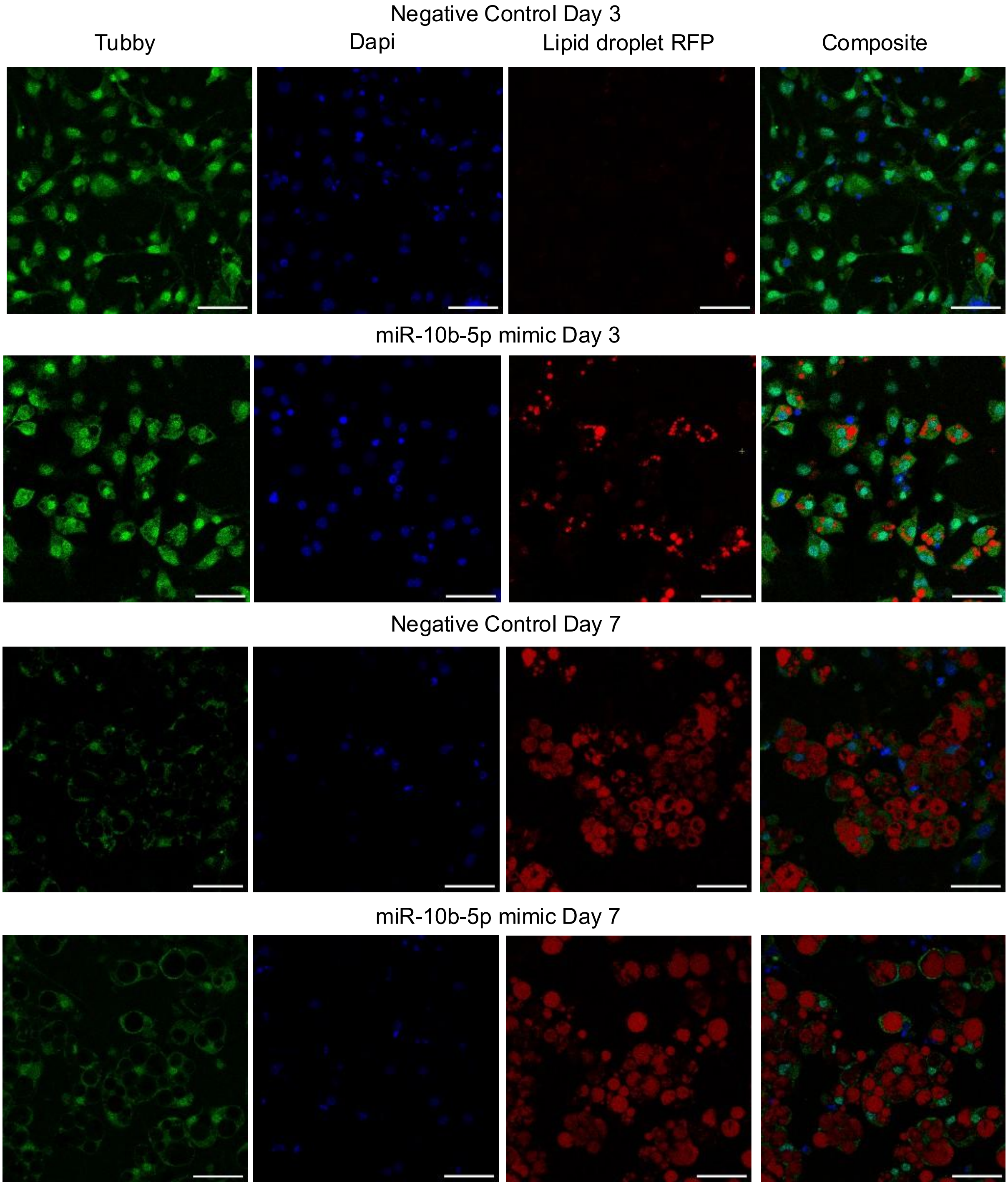

Figure S12. Tubby immunostaining during WAT differentiation in cells treated with a miR-10b mimic compared with negative-control–treated cells. Scale bar: 50 μm.

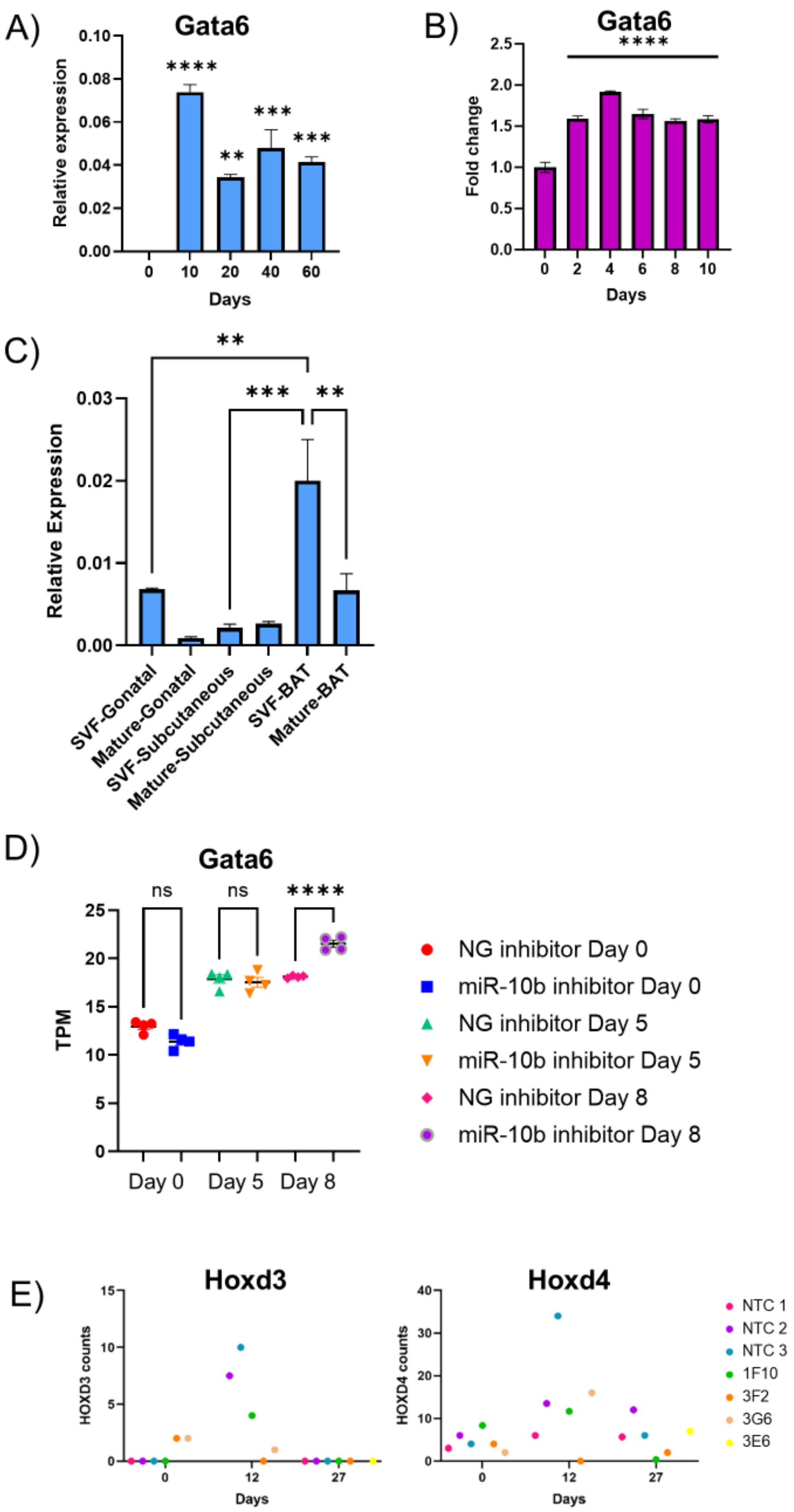
A)
Gata6
Relative expression
0.10
0.08
0.06
0.04
0.02
0.00
****
**
***
***
0
10
20
40
60
Days
B)
Gata6
****
Fold change
2.5
2.0
1.5
1.0
0.5
0.0
0
2
4
6
8
10
Days
C)
**
***
**
Relative Expression
0.03
0.02
0.01
0.00
SVF-Gonatal
Mature-Gonatal
SVF-Subcutaneous
Mature-Subcutaneous
SVF-BAT
Mature-BAT
D)
Gata6
ns
ns
****
TPM
25
20
15
10
5
0
Day 0
Day 5
Day 8
NG inhibitor Day 0
miR-10b inhibitor Day 0
NG inhibitor Day 5
miR-10b inhibitor Day 5
NG inhibitor Day 8
miR-10b inhibitor Day 8
E)
Hoxd3
HOXD3 counts
15
10
5
0
0
12
27
Days
Hoxd4
HOXD4 counts
40
30
20
10
0
0
12
27
Days
NTC 1
NTC 2
NTC 3
1F10
3F2
3G6
3E6

Figure S13. A) GATA6 expression during human ESC differentiation to BA. Data ($n \geq 3$ independent experiments) are presented as mean ± SEM. Statistical significance was determined by two-way ANOVA ($p < 0.05$, **$p < 0.005$, ***$p < 0.0005$, ****$p < 0.0001$) relative to day 0. B) Gata6 expression during mouse BA differentiation. Data ($n \geq 3$) are shown as means + SEM (two-way ANOVA test, ****$p < 0.0001$). C) Tub expression across different adipose tissue depots (two-way ANOVA test, $n \geq 2$). D) TPM levels of Gata6 expression during mouse BA differentiation, as measured by RNA sequencing. Data ($n \geq 3$) are shown as means + SEM (two-way ANOVA test, ns: not significant). E) RNA-seq profiling of mESCs undergoing adipogenic differentiation at days 0, 12, and 27 demonstrates consistently low transcript counts for Hoxd3 and Hoxd4.*
