# Supplementary material for: miR-10b-5p regulates adipocyte lineage commitment and adipogenesis via targeting of Gata6 and Tubby
Source: Cell Commun Signal. 2026 Mar 28;24:276. doi: 10.1186/s12964-026-02834-y (PMC13151333; doi:10.1186/s12964-026-02834-y)
Supplement: Supplementary file 2 — Supplementary Material 2. [file 12964_2026_2834_MOESM2_ESM.pptx]

## Slide 1
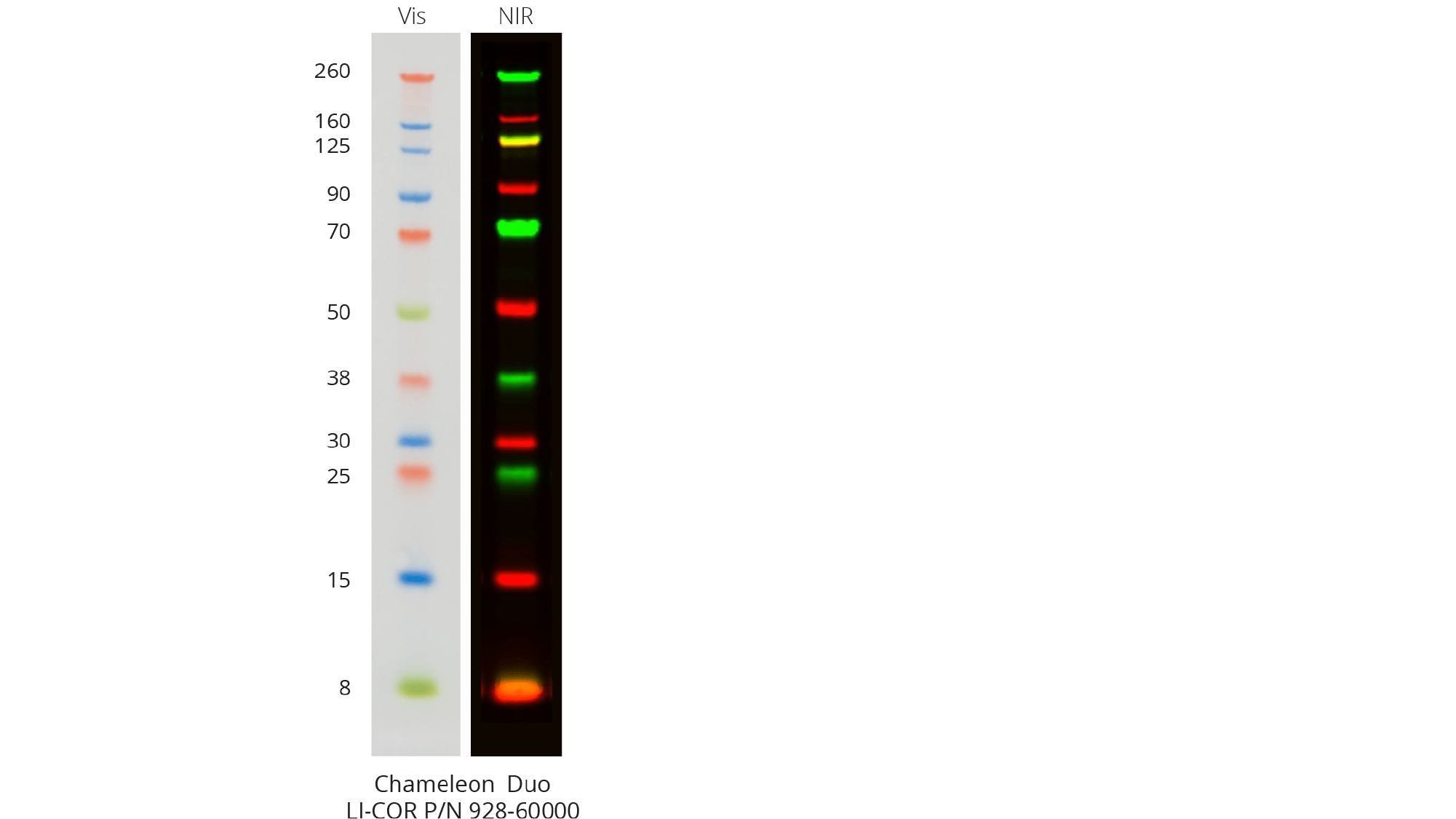

## Slide 2
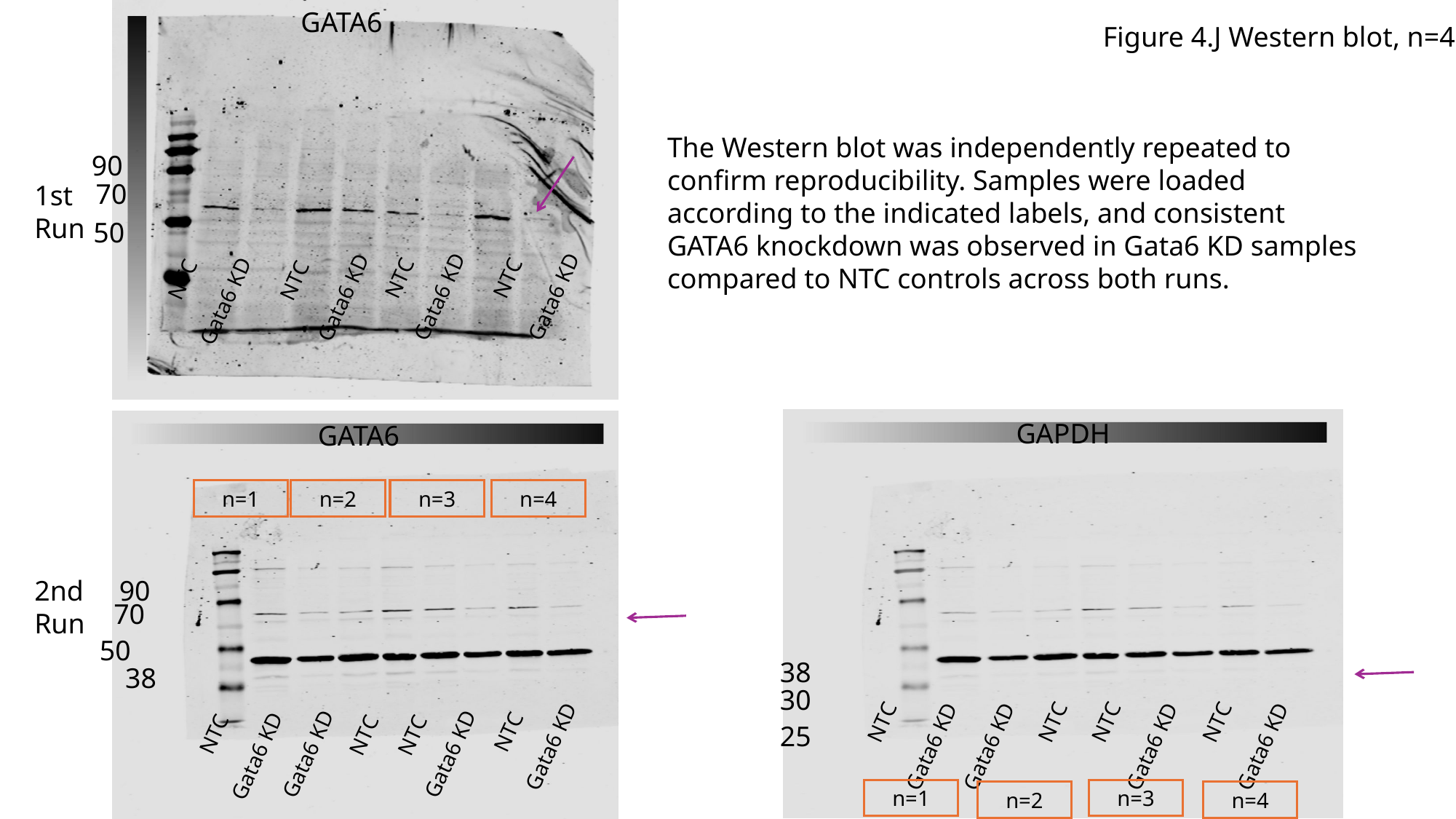

GATA6
Figure 4.J Western blot, n=4
The Western blot was independently repeated to confirm reproducibility. Samples were loaded according to the indicated labels, and consistent GATA6 knockdown was observed in Gata6 KD samples compared to NTC controls across both runs.
90
70
1st
Run
50
NTC
NTC
NTC
NTC
Gata6 KD
Gata6 KD
Gata6 KD
Gata6 KD
GAPDH
GATA6
n=1
n=2
n=3
n=4
90
2nd
Run
70
50
38
38
30
NTC
NTC
NTC
NTC
Gata6 KD
Gata6 KD
Gata6 KD
Gata6 KD
NTC
NTC
NTC
NTC
25
Gata6 KD
Gata6 KD
Gata6 KD
Gata6 KD
n=1
n=3
n=2
n=4

## Slide 3
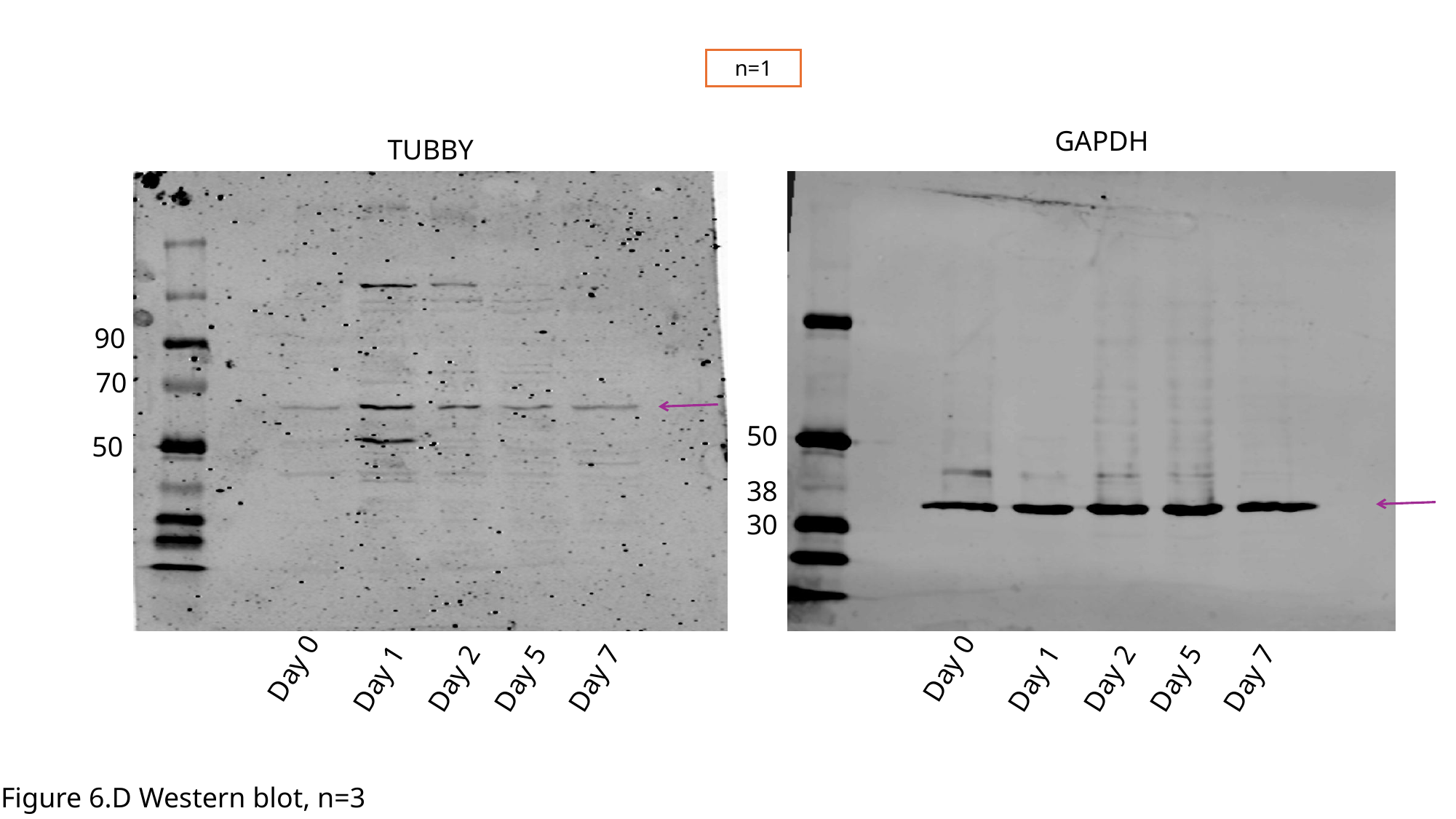

n=1
GAPDH
TUBBY
90
70
50
50
38
30
Day 0
Day 0
Day 5
Day 5
Day 7
Day 7
Day 2
Day 1
Day 2
Day 1
Figure 6.D Western blot, n=3

## Slide 4
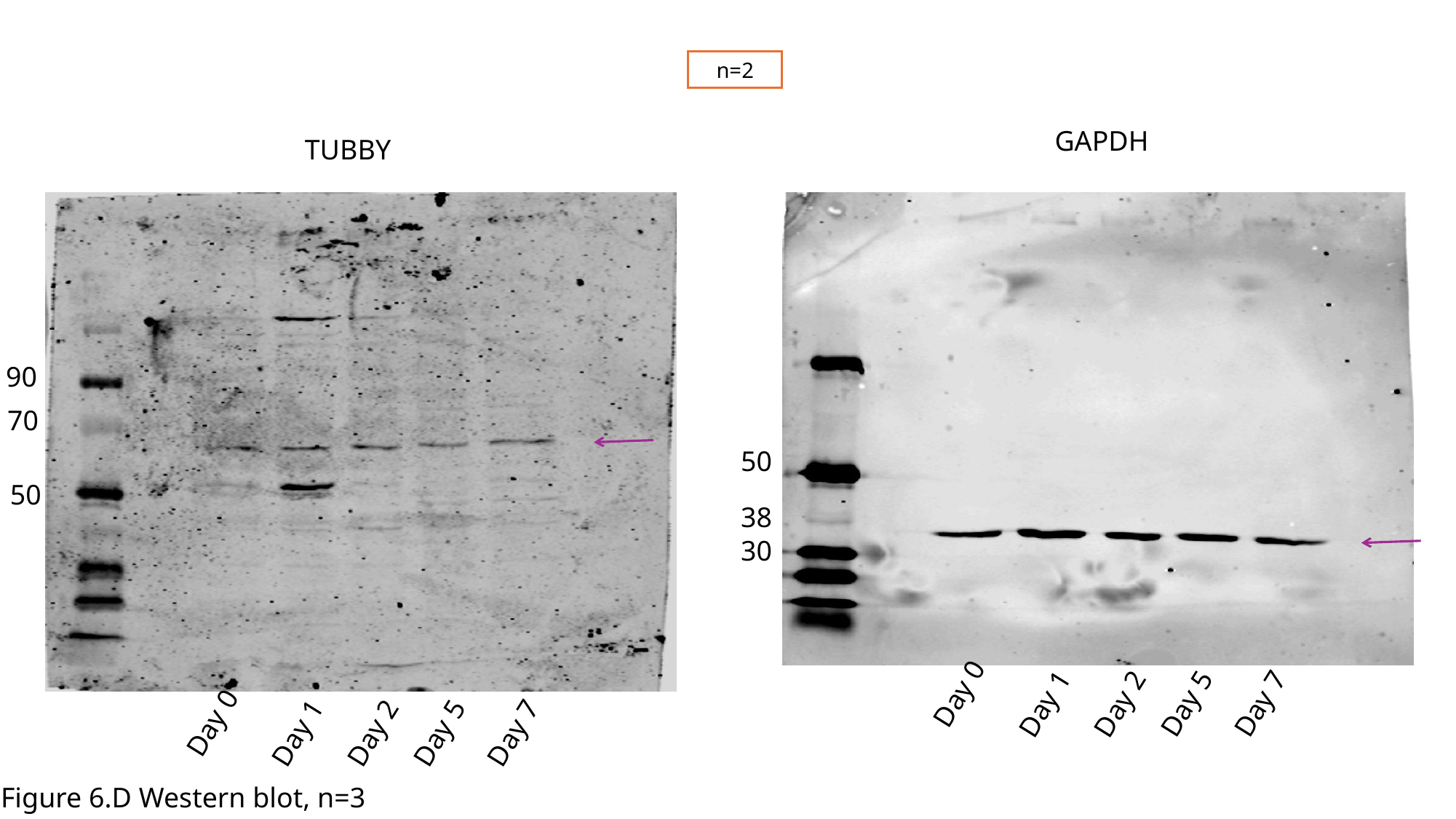

n=2
GAPDH
TUBBY
90
70
50
50
38
30
Day 0
Day 5
Day 7
Day 2
Day 1
Day 0
Day 5
Day 7
Day 2
Day 1
Figure 6.D Western blot, n=3

## Slide 5
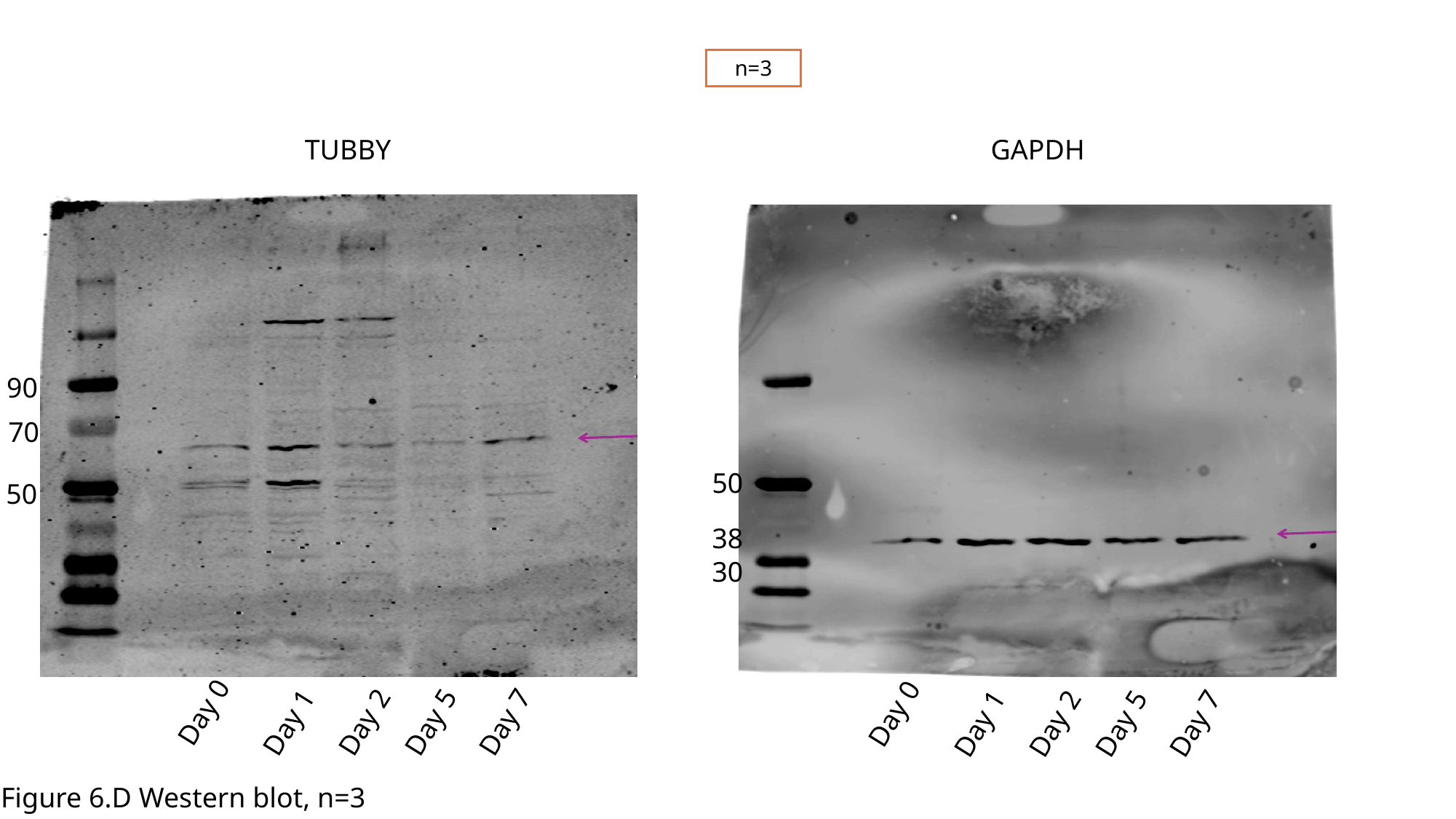

n=3
TUBBY
GAPDH
90
70
50
50
38
30
Day 0
Day 0
Day 5
Day 7
Day 2
Day 1
Day 5
Day 7
Day 2
Day 1
Figure 6.D Western blot, n=3

## Slide 6
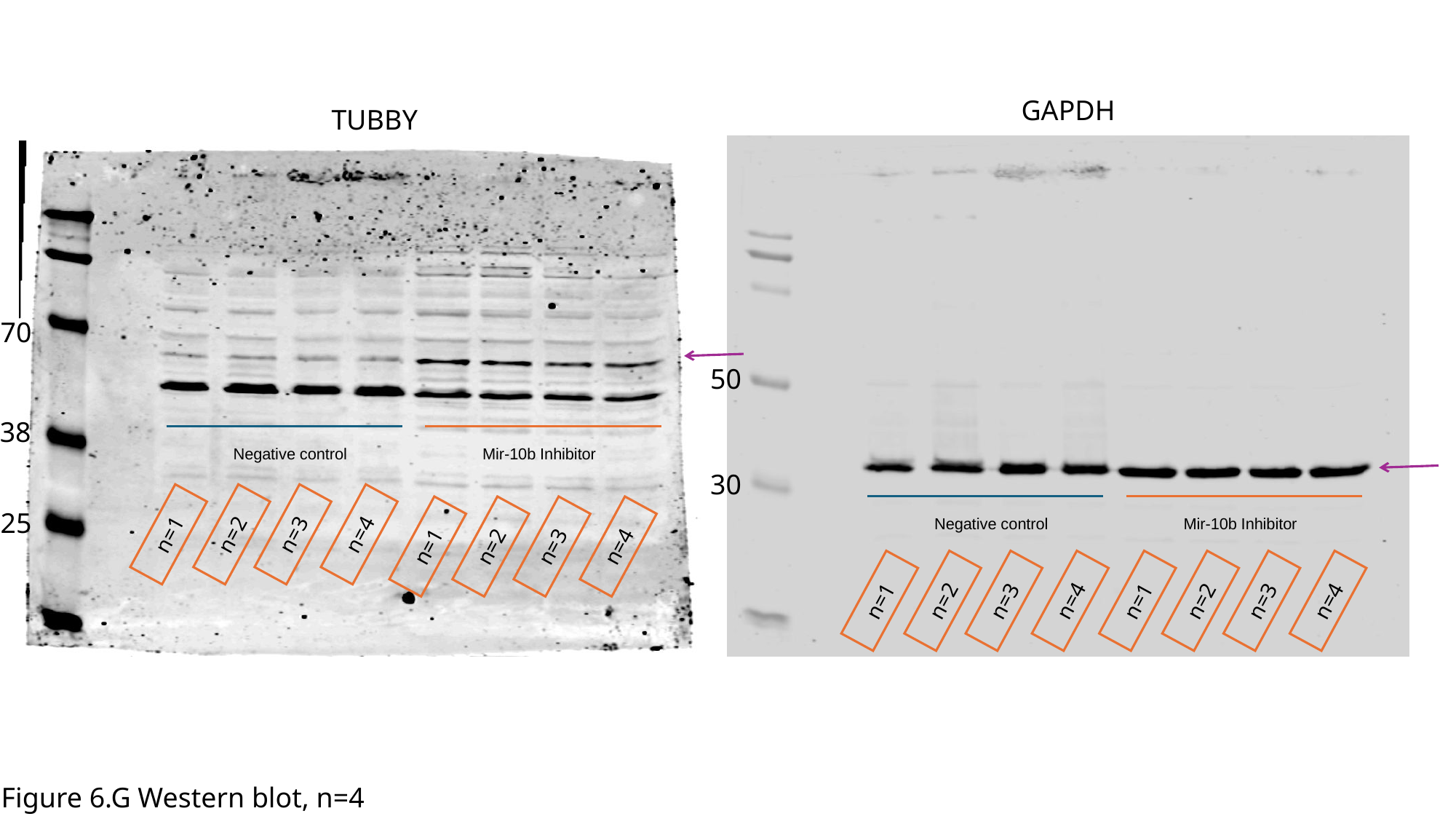

GAPDH
TUBBY
70
50
38
Mir-10b Inhibitor
Negative control
30
25
Mir-10b Inhibitor
Negative control
n=1
n=2
n=3
n=4
n=1
n=2
n=3
n=4
n=1
n=2
n=3
n=4
n=1
n=2
n=3
n=4
Figure 6.G Western blot, n=4

## Slide 7
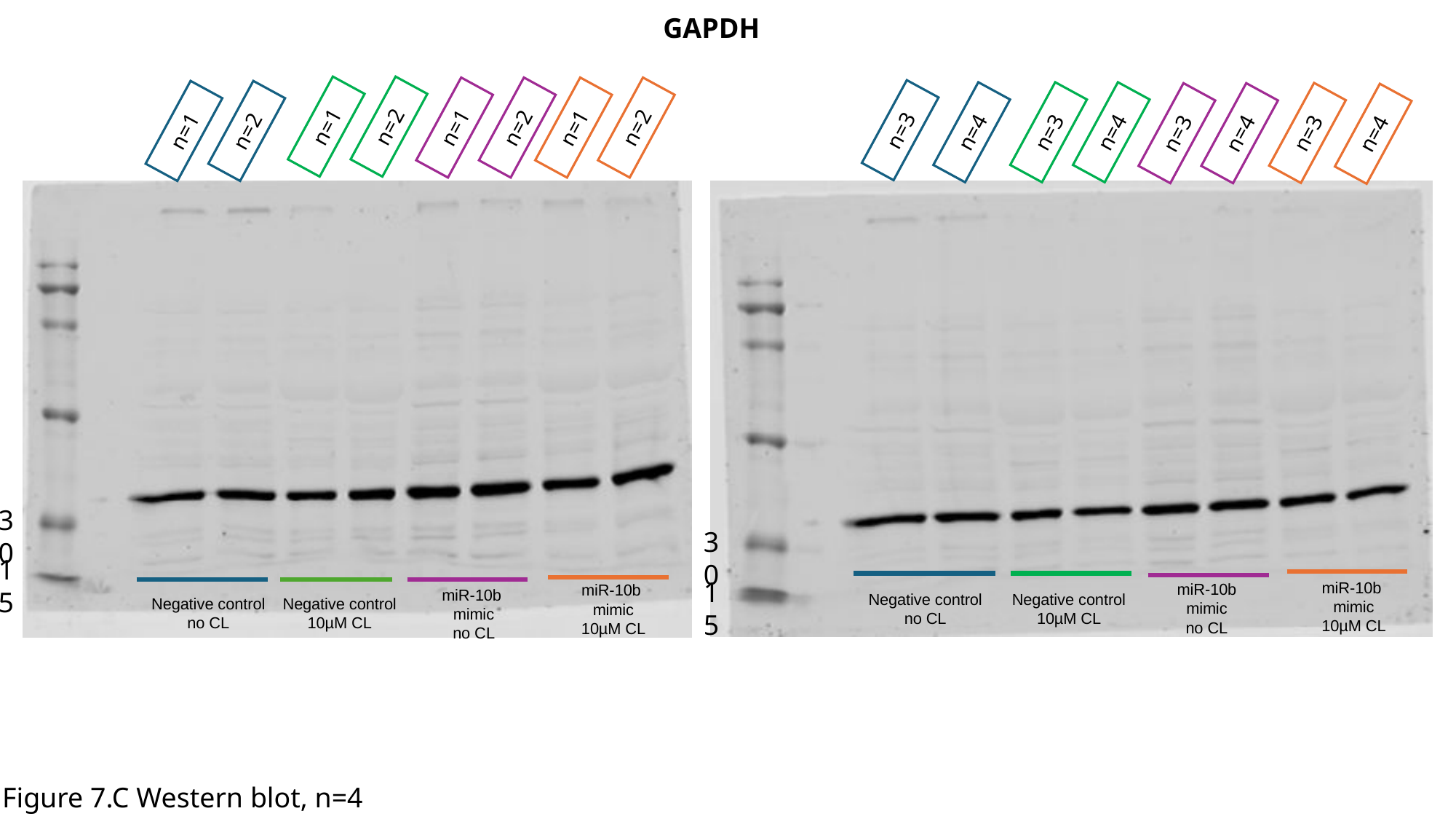

GAPDH
n=1
n=2
n=1
n=2
n=1
n=2
n=3
n=1
n=2
n=4
n=3
n=4
n=3
n=3
n=4
n=4
30
30
15
15
miR-10b
mimic
10µM CL
miR-10b
mimic
no CL
miR-10b
mimic
10µM CL
miR-10b
mimic
no CL
Negative control
no CL
Negative control
10µM CL
Negative control
no CL
Negative control
10µM CL
Figure 7.C Western blot, n=4

## Slide 8
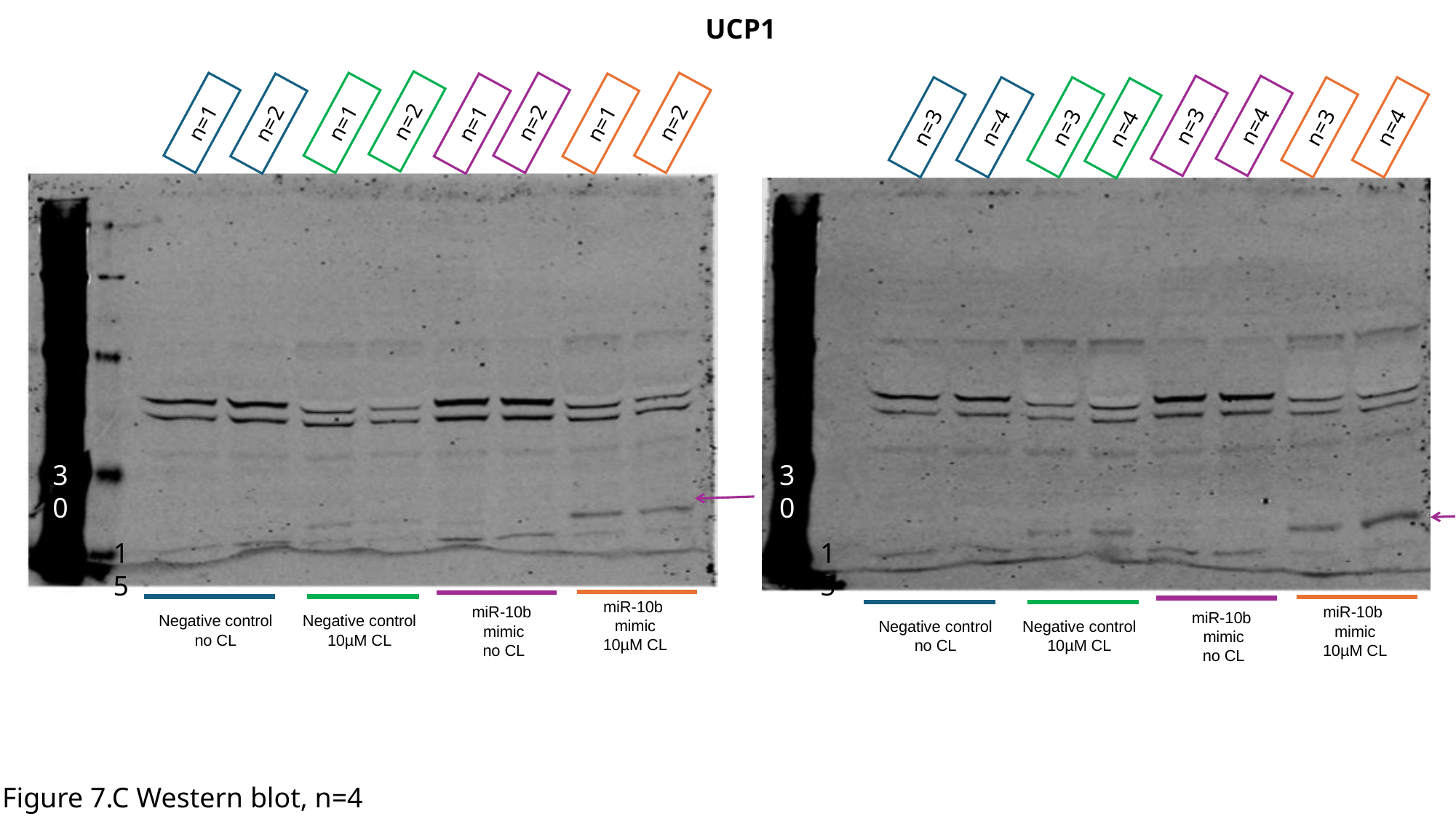

UCP1
n=2
n=2
n=1
n=1
n=2
n=2
n=1
n=1
n=3
n=4
n=3
n=4
n=3
n=3
n=4
n=4
30
30
15
15
miR-10b
mimic
10µM CL
miR-10b
mimic
no CL
miR-10b
mimic
10µM CL
miR-10b
mimic
no CL
Negative control
no CL
Negative control
10µM CL
Negative control
no CL
Negative control
10µM CL
Figure 7.C Western blot, n=4
